# Trend estimation of sub-national level daily smoking prevalence by age and sex in Australia

**DOI:** 10.18332/tid/183804

**Published:** 2024-02-23

**Authors:** Sumonkanti Das, Bernard Baffour, Alice Richardson

**Affiliations:** 1 School of Demography, ANU College of Arts and Social Sciences, The Australian National University, Canberra, Australia; 2 Statistical Support Network, The Australian National University, Canberra, Australia

**Keywords:** hierarchical Bayesian approach, joinpoint regression, national health survey, small area estimation, time-series model

## Abstract

**INTRODUCTION:**

Despite that the smoking prevalence has considerably declined in Australia after successful public health strategies over many decades, smoking is still the leading cause of preventable diseases and death in Australia. These declines have not occurred consistently across all geographical–demographic domains. In order to provide an evidence base for monitoring the trend towards the goal of reducing smoking across all domains in Australia, this study aims to estimate trends of smoking prevalence for small domains cross-classified by seven age groups (18–24, 25–29, 30–39, 40–49, 50–59, 60–69, and ≥70 years), two genders, and eight states and territories over twenty years (2001–2021).

**METHODS:**

Direct estimates of smoking prevalence for the target small domains were calculated from the micro-data of the Australian National Health Surveys conducted in seven rounds during 2001–2021. The obtained direct estimates were then used as input for developing time-series models expressed in a hierarchical Bayesian structure as a form of small-area estimation. The developed models borrow cross-sectional, temporal, and spatial strength in such a way that they can interpolate smoking levels in the non-survey years for all detailed level small domains. Smoothed trends of smoking prevalence for higher aggregation levels are obtained by aggregation of the detailed level trend predictions.

**RESULTS:**

Model-based small area estimators provide consistent and reasonable smoothed trends at both detailed and higher aggregation levels. Results show that the national-level trend exhibits a steeper linear decline over the study period, from 24% in 2001 to 12% in 2021, with a considerable gender difference of around 5% over the period, with males reporting a higher prevalence. Improved model-based estimates at the state level and by age also show steady declines in trends except for the Northern Territory (still above 20%) and older age groups 60–69 and ≥70 years (declining trends remain stable after 2012).

**CONCLUSIONS:**

The findings of the study identify the geographical–demographic groups that had poor improvement over the period 2001–2021, and are still behind the target of achieving lower smoking prevalence. These, in turn, will help health researchers and policymakers deliver targeted programs to the most vulnerable, enabling the nation to meet its health goals in a timely way.

## INTRODUCTION

Both policymakers and researchers highly appreciate official health outcome statistics for smaller areas. These statistics play a vital role in measuring and monitoring the progress of communities as they strive towards adopting healthier lifestyles. It was recently shown that up to two-thirds of deaths in Australian current smokers can be attributed to smoking^1^. While the overall rate of daily smoking has been declining for a number of years, the damage caused by the habit means that up-to-date and accurate estimates of the rate continue to be important.

The prevalence of the daily smoking rate amongst the Australian population as a whole declined to 12%, according to the 2021 National Health Survey^2^. However, there is a high degree of variability in this rate by state, age, and sex. In several Australian studies^3,4^, variability in smoking prevalence has been investigated by industry and occupation. Australia is not alone in needing to track health outcomes across fine-grained population subgroups. For example, in New Zealand (trends in smoking prevalence by sex^5^), Spain (geographical distribution of smoking prevalence by age and sex^6^), and the United States (trends in smoking-related mortality by age and sex^7^), research has also focused on smoking prevalence for sub-populations.

Australia is composed of six states at a subnational level: New South Wales (NSW), Victoria (VIC), Queensland (QLD), South Australia (SA), Western Australia (WA), and Tasmania (TAS). Additionally, there are two territories: the Northern Territory (NT) and the Australian Capital Territory (ACT). The state capital cities are Sydney, Melbourne, Brisbane, Adelaide, Perth, and Hobart, respectively, while Darwin and Canberra are the territorial capital cities. Due to the unique population distribution of Australia as a country, with it being one of the most sparsely populated countries in the world, but also being highly urbanized with a significant proportion (around three-quarters) living in its largest cities, there are important variations in health outcomes – attributable to how the population is distributed around the country. However, typically, most official surveys are not designed to produce consistent estimates of population health-risk factors that fully account for these rural–urban disparities. In fact, remote regions of Australia (covering about 80% of the landmass but with roughly 1% of the national population) are not included in most surveys. Their exclusion does not significantly affect the aggregate estimates that are produced. Nonetheless, there are certain states, such as the Northern Territory, where 20% of its population lives in very remote areas, where obtaining representative statistics at a disaggregated level may be severely impacted^8^.

Nationally representative surveys in Australia, such as the National Health Surveys (NHS)^2^ or the National Drug Strategy and Household Surveys (NDSHS)^9^, do not provide precise statistical information for disaggregated domains (e.g. administrative districts, subdistricts, and cross-classified domains of demographic characteristics). These disaggregated domains (also known as small areas) are not (usually) considered in the survey design and, as a result, sample sizes are not adequate (very small or providing no information) for calculating official statistics with reasonable precision, by the classical design-based direct estimator^10^. In such cases, the small area estimation (SAE) method^10^ is widely used to provide more precise estimates through borrowing strength over space and time. Multilevel time-series models utilizing SAE^11,12^ can be used to estimate improved and reliable trends of smoking prevalence for the cross-classified spatial–demographic domains, for which the direct estimates are unstable and imprecise.

Policymakers are required to monitor the trends of health outcomes as well as the rate of changes over time, which allows them to target interventions to areas of greatest need and monitor the impact of such interventions. Joinpoint trend analysis^13,14^ allows policymakers to accurately interpret the changes over time and to determine if those changes are statistically significant. Doing this allows for the effective identification and exploration of areas where there are disparities in the trends. Joinpoint analysis also accurately identifies when this change occurs and has been used to summarise trends in cancer rates^15^ in the United States, suicide rates in Denmark^16^, and stunting prevalence in Bangladesh^17^, among many other applications.

This study aims to estimate trends of daily smoking prevalence in Australia for cross-classified domains of eight states and territories (NSW, VIC, QLD, SA, WA, TAS, NT, ACT), two sexes (female and male), and seven age groups (18–24, 25–29, 30–34, 40–49, 50–59, 60–69, and ≥70 years) for the period 2001–2021 by employing model-based time-series modeling techniques to the smoking data collected in the national health surveys. The trends, therefore, present daily smoking prevalence from 2001–2021 for these 8×7×2=112 small domains. The model-based annual trend estimates are then used for joinpoint analysis. This study considers binary (male/female) information for the gender variable to make the trends comparable over time. In the discussion that follows, ‘daily smoking prevalence’ is defined by the Australian Bureau of Statistics (ABS)^2^, and this will be subsequently referred to as ‘smoking prevalence’.

## METHODS

The direct estimates of daily smoking prevalence for the cross-classified 112 domains obtained from survey data are used as data inputs for developing multilevel time-series models. The model-based trend estimates at various disaggregation levels are used to develop joinpoint regression models. This section briefly illustrates data sources, direct estimation of daily smoking prevalence, and statistical modeling through multilevel time-series analysis and joinpoint trend analysis in three sub-sections.

### Data sources

The Australian NHS has been conducted at regular intervals since 1989 to collect data on health-related issues, including health-risk factors like tobacco smoking and alcohol consumption. Since 2001, the survey has been administered on a three-year cycle in 2001, 2004–2005, 2007–2008, 2011–2012, 2014–2015, 2017–2018, and 2020–2021^2,8,18-22^. This study concentrates on surveys carried out since 2001, as their designs are approximately comparable over time. In each survey, a sample of private dwellings is selected through a stratified multistage sampling design.

The sample design of the NHS ensures that within each state or territory, each person has an equal chance of selection. This is accomplished by dividing Australia into a number of strata, which are defined by splitting states and territories into geographically homogeneous (usually contiguous) areas. Each stratum consists of a number of Census Collection Districts (CDs), which were selected with probability proportional to size (the number of dwellings) from each stratum. A systematic random sample of dwellings was selected from the selected CDs. Finally, within each selected dwelling, one adult (aged ≥18 years) and one child aged 0–17 years (if available) were randomly selected for inclusion in the survey to collect more information from each respondent. To take account of possible seasonal effects on health characteristics, the data collection was spread uniformly and randomly across an enumeration period of 10–12 months.

In our analysis, we restrict our sample to the adult population aged ≥18 years. In the seven consecutive surveys, 17891, 20780, 15779, 15475, 14560, 16370, and 10116 adults were observed, to calculate the direct estimates of daily smoking prevalence for the considered 112 domains. In all the surveys, the total number of surveyed adults was found to be the lowest in NT (ranging from 111 in 2008 to 1088 in 2017), and consequently, the sampling errors were always found to be higher for the domains for NT. For the domains by age and sex, the total number of participants has always been found to be smaller in the recent 2020–2021 NHS, due to surveying during the COVID-19 period. The proportion of female participants is found to be about 10% higher than that of males in all the surveys. This pattern of sex distribution is also observed for most of the age groups except the 18–24 and ≥70 years age groups. The distribution of the number of participants by age, sex, and state for each survey is given in the Supplementary file.

### Direct estimates

In our analysis, our target response variable is the proportion of people (aged ≥18 years) who are classified as ‘smokers’ according to the NHS, according to their self-reported description of their smoking status at the time of the interview. This smoking prevalence rate is defined as those who report being ‘daily smokers’ in a cross-classified (state–age–sex) domain. The smoking prevalence is then estimated for each of the 112 domains (i.e. the cross-classification of the eight states and territories, two sexes, and seven adult age groups: 18–24, 25–29, 30–39, 40–49, 50–59, 60–69, and ≥70 years), in the seven survey years (so there are 8×2×7×7=784). Consequently, the units of analysis in this study are the 2352 = 112× 21 domains covering the entire 21-year period comprising both survey and non-survey years (i.e. 7 survey years and 14 non-survey years). However, only 767 state–age–sex–year domains covered in survey years are used to fit the models. There are some state–age–sex cross-classified domains with no sample in the survey years, e.g. people aged ≥70 years in the Northern Territory. The domains for the remaining non-survey years are defined as missing in the model development stage to correctly specify the period-to-period evolution of the trend, which helps to predict the target parameters for the non-survey years’ domains based on the developed models.

In practice, survey responses are affected by complex sampling designs and differential non-response propensities, and a failure to fully include these in the prevalence estimates of health outcomes may lead to inaccuracies. We, therefore, use the survey-weighted estimates (and estimated standard errors) of the daily smoking prevalence at the detailed state–age–sex level as inputs for the direct estimates. These weighted estimates are calculated using sampling weights, while their standard errors are estimated using a replication method for the complex sample designs and weighting procedures employed in the NHSs^8^. Replicate weights are produced in all the NHSs under the delete-a-group jackknife method of replication and are given in the micro-data^23^. The estimates and the standard errors based on the replication methods are calculated as:


$$\begin{array}{l} {\hat{y}}_{it}=\sum_{j=1}^{{\text{n}}_{\text{it}}}w_{ijt}y_{ijt}\\ \text{and}\\ \text{var}({\hat{y}}_{it})=R^{-1}(R-1)\sum_{c=1}^{60}\left({\hat{y}}_{it}^{\left(c\right)}-{\hat{y}}_{it}\right) \end{array}$$

where *R* is the number of replicate weights, y_ijt_ and w_ijt_ are the values of target outcome variable (say, 1 for daily smoking and 0 for non-smoking) and the sampling weight, respectively, for the j individual of the i domain in year t. The replicate version of the survey-weighted direct estimate^23^ is *ŷ*_*it*_^(c)^ for each replicate weight *c*=1, 2, …, *R*. In 2001, *R*=30 and in later surveys, *R*=60 (see detailed sampling design in survey reports^2,8,18-22^). The replicate weights are used in the direct estimation to make the estimates nationally representative and make the time series of direct estimates comparable over time. Multilevel time-series models have been described^11,12,17^ and can be developed assuming the direct estimates follow a Gaussian process.

Since the direct estimates and their standard errors are found to be positively correlated with evidence of some heteroskedasticity, a suitable transformation (such as the square-root transformation) is required to reduce both correlation and heteroscedasticity. In such a case, back-transformation is also required to reduce bias. However, this can lead to perplexing trend patterns in the survey years if the standard errors are not smoothed adequately^12^. To avoid these issues, a multilevel model can be developed, assuming the domain-specific sampled number of smokers follows a non-Gaussian process, such as a binomial process^24,25^. Thus, the weighted number of smokers has been used as the outcome variable to account for the survey design in developing the multilevel time-series model^25^. This approach helps to avoid the strong (and untestable) assumption that the estimated sampling error variances can approximate the true error variances^26^.

### Statistical modeling

We integrate two key approaches: 1) employing a standard multilevel model that connects survey data with area-specific information, leveraging strength to address the limited observations in specific small domains and enhancing the effective number of observations used for estimation within those domains; and 2) utilizing time-series models to connect successive surveys, interpolating for non-survey years, ensuring the availability of annual (smoothed) domain-specific estimates. We estimate trends in smoking prevalence at various levels of disaggregation using multilevel time-series models. We fit the models in a hierarchical Bayesian framework using Markov Chain Monte Carlo (MCMC) simulations for their relative efficiency in modeling complex dependence and ability to make out-of-sample predictions in the case of sparseness. Under this framework, the detailed model is developed, firstly to correctly specify the period-to-period evolution of the trend, and secondly to allow the missing domains to be estimated using predicted draws from the MCMC simulations.

After producing reliable annual model-based estimates for the different cross-classified domains, we are interested in estimating the trends and characterizing any changes in the trends over time. We used joinpoint regression analysis to partition the 21-year period into segments where this rate of change is constant, based on transition points delineating these segments. We calculate the average change over these segments covering specific periods to summarize and compare trends over the 21-year period.

A detailed exposition of the statistical modeling, model selection, comparison, and validation is provided in the Supplementary file.

### Multilevel time-series model

To account for the varying time-lags of 3 or 4 years between the subsequent survey years, multilevel time-series models are defined at an annual frequency at the detailed level of 8×7×2=112 domains, which are a cross-classification of seven age groups, two genders, and eight states and territories. Consequently, there are 112×21=2352 domains over the time period of 21 years, and only 767 state–age–sex–year domains have data to fit the multilevel time-series models. The domains for the remaining non-survey years are defined as missing in the model development stage to correctly specify the period-to-period evolution of the trend, which helps to predict the target parameters for the non-survey years’ domains based on the developed multilevel time-series models.

To define the multilevel time-series model for the outcome values, let *ŷ*_dt_ denote the number of smokers (or daily smoking prevalence) for domain *d* and year *t*. The index d ranges from 1 to *M_d_* =112 and *t* from 1 to *T* =21 years. Direct estimates *ŷ*_dt_ are combined in a large vector of dimension *M_d_T* dimension as *ŷ*=(*ŷ*_11_,*ŷ*_21_,…*ŷ*_Md1_,…, *ŷ*_1T_,…, *ŷ*_MdT_)′ to define a hierarchical Bayesian multilevel time-series model for *ŷ* as a general linear additive form:

*ŷ~f(μ, φ) ; g(μ)=η=Xβ+Σ_α_Z^(α)^v^(α)^*

where *f* is a probability distribution depending on the vector of *ŷ* with an optional scale or dispersion parameter *φ* and *g* is a link function that links the mean vector to the linear predictor *η*, and *X* is an *M*×*p* design matrix for a p-vector of fixed effects *β*, and the *Z^(α)^* are *M*×*q(α)* design matrices for *q(α)*-dimensional random effect vectors *v(α)*. If *ŷ* consists of count values, we assume *f* is a binomial distribution with mean vector ***μ*** and logistic link function as *g*(***μ***)=*log*{***μ***(1-***μ***)^-1^}; if *f* is a normal distribution, then the link function *g(****μ****)* will be an identity function. The term summed over (α) indicates that several possible random effects terms at different levels (e.g. local level and smooth trends at state/territory levels) can be added to the model.

The vector *β* of fixed effects is assigned a very weakly informative normal prior *p(β)*=*N* (0,100*I*). The second term on the right-hand side of *η* consists of a sum of contributions to the linear predictor by random effects or varying coefficient terms. The random effect vectors *v^(α)^* for different α are assumed to be independent, but the components within a vector *v^(α)^* are possibly correlated to accommodate temporal, spatial, or cross-sectional correlation. Spatial and temporal variations are modeled through intrinsic conditional autoregressive and random walk models, respectively. The details about the formation of various random effects components are published^11,12^.

The models are run in R software^27^ using the package *mcmcsae*^28^. For the comparison of models using the same input data, the Widely Applicable Information Criterion or Watanabe-Akaike Information Criterion (WAIC) and the Deviance Information Criterion (DIC) are used. The leave-one-out cross-validation information criterion (LOOIC) and expected log predictive density (ELPD) are also calculated for model comparison using the R package *loo*^29^. The details of these model information criteria are published^30^. A number of competitive multilevel time-series models are developed, and then a final model shown in the Results section is selected based on the model performance. The final multilevel time-series model is developed using 1000 burn-in and 10000 iterations, of which the draws of every fifth iteration are stored, and consequently, 3×2000 = 6000 draws are used to compute estimates and standard errors. Longer simulations of the selected model provide Gelman-Rubin potential scale reduction factors (known as R-hat) <1.1 and sufficient effective numbers of independent draws for all model parameters and model predictions. The relevant posterior predictive checks are also done to look for any systematic discrepancies between the real and the MCMC simulated data^31^.

Further, we maintain internal consistency by ensuring that the trends at the aggregate levels (national, state/territory, age group, and sex) are consistent with the corresponding direct estimates at the survey years. This comparison will assess the bias of the model-based estimates at the higher aggregation level, where the direct estimates are assumed to be consistent.

### Joinpoint trend analysis

Joinpoint trend analysis^13^ is a valuable tool for making inferences about changes in trends over time. We conduct a joinpoint trend analysis to examine how the changes in smoking prevalence have progressed over time in Australia. Doing this shows that the declines in smoking prevalence have not changed at a constant rate over the entire period. In particular, we see that there are segments of the period that are characterized by slighter gradual decreases, while others exhibit much sharper declines.

Joinpoint trend models are important in our situation because they allow us to identify specific change points (i.e. where the rates of change in the trend change). Annual percentage change (APC) for each segment and average annual percentage change (AAPC) for the considered time period is computed, with 95% confidence intervals (CIs), to show whether these changes are statistically significant. The annual time-series data of daily smoking prevalence obtained from the model-based estimator are utilized for the joinpoint analysis and are conducted using the Joinpoint Regression Software^14^. In particular, as for our situation when the trend is not constant over time over the entire 21-year period, we can characterize this non-linearity in the trend by using the annual percent change from the segmented analysis^13^.

The values of APC and AAPCs for the trend estimates of smoking prevalence are calculated at both aggregated (national, sub-national, age, and sex) and disaggregated domains (cross-classification of age, sex, states, and territories). For a particular domain (say, 18–24 years female from NSW), the values of APCs are calculated first, and then an AAPC value is calculated as the weighted average of the APC values. We report selected results here, but more detailed results and mathematical derivations of the joinpoint analysis are provided in the Supplementary file.

## RESULTS

This section is sub-divided into three sub-sections to present: 1) the development of a multilevel time-series model for the smoking prevalence at the detailed (state–age–sex–year) level; 2) the trends of smoking prevalence obtained from the developed model; and 3) the findings from the joinpoint analysis of model-based trend estimates of smoking prevalence during 2001–2021.

### The developed model

In developing the time-series models for daily smoking prevalence, sex, age group, state, and territories are considered time-invariant variables, while the standardized year (Year.std) variable is the time-variant variable for the fixed effects components. Among the two-dimensional interactions of time-invariant variables, the interaction of sex and age class has a significant contribution in explaining the variation of smoking prevalence. The linear time-trend of smoking prevalence is modeled by incorporating a standardized year variable as the fixed effects component. In addition, random intercepts and slopes of time trends varying over the detailed (state–age–sex) level domains are incorporated as one random effects component. This component captures both cross-sectional and temporal strengths among the considered cross-classified domains (state–age–sex–year). To account for temporal variability by age, sex, state, and their interactions, random walk models of both first-order and second-order have been examined. However, only the first-order random walk (RW1) model at the sex-by-age level domains is found to have a significant contribution to the model. The RW1 trends are specified at the sex-by-age level with a scalar variance structure, which indicates that the variation in trends is assumed to be fixed for all the sex-by-age domains. This simpler specification of the variance structure is a more parsimonious fit to the data.

Since the models are developed through MCMC simulation, the means and standard deviations over the MCMC draws are used as trend estimates and standard error estimates, respectively, at the most detailed level. The trend estimates of daily smoking prevalence at higher aggregation levels such as country, state and territories, age, sex, and their two-dimensional domains are computed by aggregating the MCMC simulation results at the state–age–sex–year level. Aggregations at higher levels are the weighted average of the estimated detailed level smoking prevalence, using the total number of adults aged ≥18 years of the estimated population in Australia. These domain-specific estimated populations are extracted from the quarterly population estimates provided by the Australian Bureau of Statistics^32^ under Estimated Resident Population (ERP) statistics. To select the best model, firstly, the developed models are compared based on model performance criteria (such as WAIC, DIC, LOOIC, and ELPD) as well as posterior predictive checks. These results are also validated by comparison to other existing national surveys, such as the National Drug Strategy Household Survey, and the trend estimates over time are examined for consistency.

The linear predictor of the finally selected model can be written, element-wise for a domain *d* and year *t*, as:


$$\begin{array}{l} g(\mu_{dt})=log\left[\frac{\mu_{dt}}{1-\mu_{dt}}\right]=\beta \prime x_{dt}+v_{d}+z_{t}v_{d}^{\left(yr\right)}+u_{dt}^{\left(sex-age\right)}\\ \left(\begin{array}{c} v_{d}\\ v_{d}^{\left(yr\right)} \end{array}\right)∼N\left[\left(\begin{array}{c} 0\\ 0 \end{array}\right),\left(\begin{array}{cc} \sigma_{I}^{2} & 0\\ 0 & \sigma_{S}^{2} \end{array}\right)\right]\\ u_{dt}^{\left(sex-age\right)}-u_{d(t-1)}^{\left(sex-age\right)}∼N(0,\sigma_{RW1}^{2}) \end{array}$$

where *v_d_* is random intercept varying by the detailed state–age–sex level domains, *z_t_* is the standardized value for year *t*, *ν_d_^(yr)^* is the random slope varying over the state–age–sex level domains, and $u_{dt}^{\left(sex-age\right)}$ is the sex-age level temporal random effects. This temporal term is assumed to follow a random walk (RW1) to capture the unsystematic short-term fluctuations and has the benefit of imposing a certain level of smoothness over time. The fixed effects component *β' x_dt_* consists of the interaction of sex and age groups.

The estimates of the fixed and random effects parameters are shown in Table 1. The final model includes fixed effects parameters to capture the age-specific differences in smoking prevalence, and we add a linear trend term to account for the year-on-year change. We also include fixed effects at the state and territory levels. An interaction term that allows the age effects to vary by sex is also included. In regard to the random effects, we include: 1) a random walk term to capture the long-term direction of the trend; and 2) random intercept and random slope terms varying over the detailed cross-classified domains are included recognizing that there are similarities between prevalence in domains that are not fully captured by the fixed effects. This final model has the advantage of ensuring that the estimates use information from similar domains for improved reliability in the estimation of the underlying smoking prevalence rates without relying solely on the observed sparse data. As can be seen in Table 1, these components are found to be statistically significant.

**Table 1:** Estimated values of fixed effects and random effects parameters along with the corresponding t-values and R-hat values, for the developed multilevel time-series model† of daily smoking prevalence in Australia, based on the Australian Nation Health Survey years 2001, 2005, 2008, 2012, 2015, 2018, and 2021

*Parameters*	*Mean*	*t-value*	*R-hat^§^*
**Fixed effects**			
**Intercept**	-2.03	-34.79*	1.00
**Year.std**	-0.23	-5.91*	1.00
**Sex: Male**	0.24	3.80*	1.00
**Age** (years)			
25–29	0.20	2.95	1.00
30–39	0.15	2.55	1.00
40–49	0.19	3.18	1.00
50–59	0.07	1.13	1.00
60–69	-0.35	-5.47	1.00
≥70	-1.21	-16.77	1.00
**State**			
NSW	0.26	6.31*	1.00
VIC	0.25	6.00*	1.00
QLD	0.38	9.53*	1.00
SA	0.31	7.37*	1.00
WA	0.22	5.03*	1.00
TAS	0.52	11.55*	1.00
NT	0.65	11.91*	1.00
**Males-by-age** (years)			
25–29	0.19	2.21*	1.00
30–39	0.20	2.57*	1.00
40–49	0.06	0.78	1.00
50–59	0.04	0.54	1.00
60–69	-0.04	-0.47	1.00
≥70	0.03	0.30	1.00
**Random effects**			
$\sigma_{I}^{2}$	0.04	2.27*	1.02
$\sigma_{S}^{2}$	0.04	2.28*	1.01
$\sigma_{\mathrm{RW}1}^{2}$	0.07	6.88*	1.01

* |t|>1.96 indicates statistical significance.

§ R-hat<1.1 confirms convergence and efficiency diagnostics for Markov Chains.

† Direct estimates of the domain-specific number of smokers obtained from the surveys are used in the time-series model development for the detailed 112 cross-classified domains of seven age groups (18-24, 25-29, 30-39, 40-49, 50-59, 60-69 and ≥70 years), two sex (male and female) groups, six states (NSW, VIC, QLD, SA, and WA) and two territories (NT and ACT).

### Trends of smoking prevalence

Before proceeding with the results and drawing any inferences, it is important to ensure that the model-based estimates from the developed multilevel time-series model are appropriate, provide reasonable results, and substantially improve upon the direct estimates. To provide some indication of the improvement provided by small area estimation when compared with the direct estimates, we compute the coefficient of variation (CV) for each of the state–age–sex domains. The CV provides a measure of relative errors and gives an indication of the precision of the model-based estimates when contrasted with the direct estimates. As per the Australian Institute of Health and Welfare (AIHW) standards, official statistics with a relative standard error of 25% to 50% should be used with caution^9^. We, therefore, use a cut-off of 25%, and subsequently, CVs of greater than 25% indicate that small area estimation is required because the direct estimates are not very accurate and show unacceptable levels of imprecision^6,33^.

**Figure 1:**
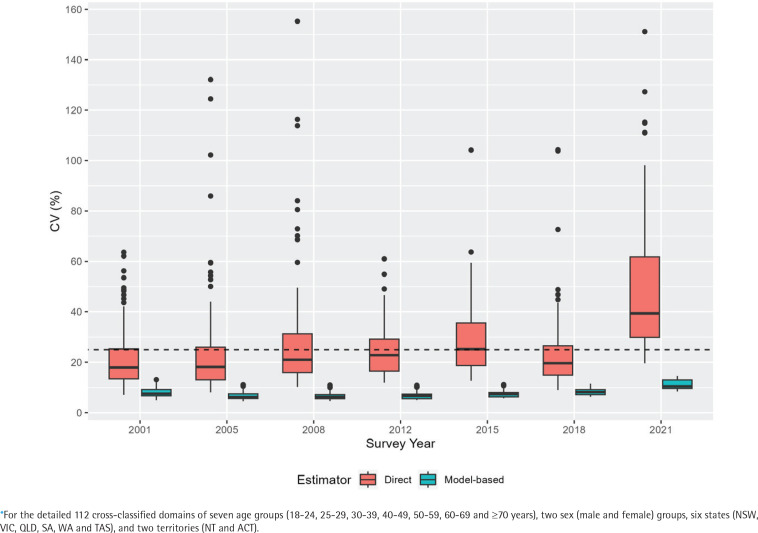
Distribution of the coefficients of variation (CV, %) of estimated daily smoking prevalence in Australia, calculated by the direct (red filled box) and multilevel time-series model-based (sky-blue filled box) estimators. Direct estimates obtained from the Australian Nation Health Surveys 2001, 2005, 2008, 2012, 2015, 2018, and 2021 are used in the time-series model development* to obtain model-based estimates. The dashed horizontal line at 25 percent indicates the acceptable precision

**Figure 2:**
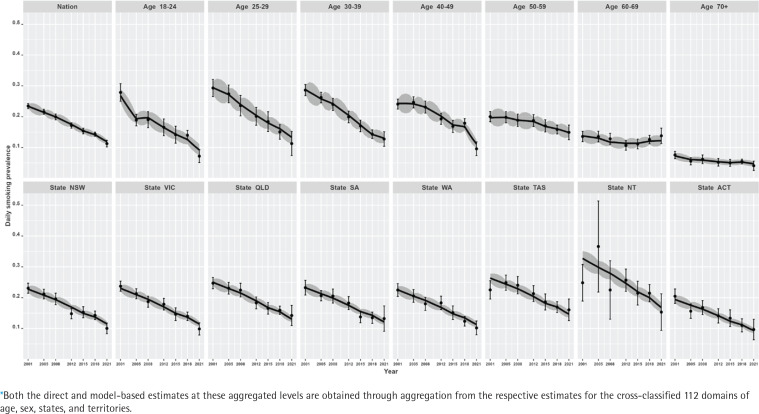
Trends of daily smoking prevalence in Australia during 2001–2021 for the nation, seven age groups, six states, and two territories (NT and ACT) aggregated levels*, estimated by the direct (error-bar line) and model-based (solid lines) estimators with their 95% confidence bands

**Figure 3:**
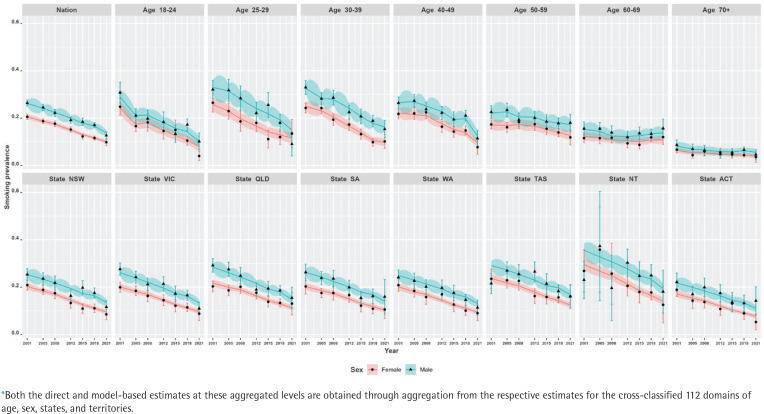
Trends of daily smoking prevalence in Australia during 2001–2021, by sex at the nation, age group, state, and territory (NT and ACT) aggregated levels* estimated by the direct (error-bar line) and model-based (solid lines) estimators with their 95% confidence bands

Figure 1 illustrates the relative standard errors (represented as CVs) for both model-based and design-based estimates. The visual representation clearly demonstrates that the model-based trend estimates are more reliable, offering a better indication of the changes in smoking prevalence in Australia. On average, the CVs of the model-based estimates are at most half the size of those of the direct estimates, suggesting greater stability and precision in the model-based estimates compared to the direct estimates. Also, in a substantial number of survey years, the CVs of the direct estimates are much larger than the 25% threshold. However, this is not the case for the model-based estimates, which report average CVs of 10%, with a maximum of 17% in all the state–age–sex domains across all years. The comparison of model-based trend estimates with those of design-based estimates shown in Figures 2 and 3 indicate that the model-based estimates are unbiased and consistent at higher aggregation levels (such as national, division, age, and their cross-classification by sex), at which levels direct estimates are reliable. Therefore, the multilevel time-series model developed in the form of small area estimation substantially improves the precision and reliability of the domain-specific trend estimates in smoking prevalence.

Our results show that at the national level, there has been a steep decline in smoking prevalence over the 21-year period (top-left plot in Figure 2). The prevalence at the beginning of the period was 24%, and there was a reported reduction by half (i.e. 12%) by the end of the period under observation in 2021. However, we also observe that there are substantial differences in this reduction when examining by sex (top-left plot in Figure 3). This difference by gender is around 5%, with males reporting higher levels of smoking than females. In addition, while these downward trends in smoking prevalence are seen when examining the changes by age, there are also remarkable differences in the age-specific trends (top-right five plots in Figure 2). Further, at a state level, these results show that there are similar declines – apart from NT, where the direct estimates are considerably unstable (shown by wide confidence intervals), although the model-based estimates provide smoother trends (bottom panel plots in Figure 2). This is due to the fact that the model-based estimators borrow strength from similar domains to overcome the lack of observations in smaller states/territories and increase the effective sample size to obtain better accuracy in the estimated trends. When the state-level trends are examined by sex in Figure 3 (bottom panel plots), the declining pattern appears similar, with considerable differences by gender in all states/territories. However, the direct estimates show that the difference by gender is the lowest in the recent 2021 survey, particularly in the three large states (NSW, VIC, and QLD), which is also observed in the model-based estimates. Put differently, there are differences between males and females in regard to smoking prevalence, but these differences were much wider in earlier periods than in more recent times.

Like the national level, linear declining trends have been observed for all the states and territories, as shown in Figure 2. It can be seen that for the larger states (i.e. NSW, VIC, and QLD), both the direct and model-based estimators provide practically consistent trend estimates. However, where we do start to see differences between the direct and model-based estimators is in the smaller states and territories (specifically in TAS and NT). For these smaller states and territories, the confidence intervals are comparatively much wider for the direct estimates, though model-based estimates show greater accuracy (i.e. tighter confidence bands). Noticeably, in particular for NT – as a result of changes in the sampling design over time^2,8,21,22^ – the estimates have wider confidence bands at the beginning survey periods when compared to the later periods due to the availability of more observations in the later surveys.

In addition, we observe that there is a non-linear pattern in the age-specific trends (Figure 2) as well as their cross-classification by sex (Figure 3). Therefore, to account for this, we examined different parameters in the model and found that including a temporal age-specific component (specified as a first-order random walk) was statistically significant. Sex- and state-specific temporal components were also examined, but these were found to be non-significant. In other words, after including the age-specific non-linear trends, the remaining sex- and state-specific trends were assumed to decline in a linear manner.

Similarly, there are differences in the declines by age. Smoking prevalence declines non-linearly and most dramatically for the younger age groups (18–24 to 40–49 years), for which smoking prevalence is higher, while the trends remain flat for the older age groups, for which smoking prevalence is lower. For the oldest age group, ≥70 years, this remains stable at around 5% after 2012. Non-linear trends are more explicit for the 25–29 and 30–39 years age groups, for which the smoking prevalence halves over the 21-year period. In contrast, the rate of decline in smoking prevalence is much slower for the older age groups (especially for the oldest age group, ≥70 years). As expected, the model-based estimates have wider confidence bands for the older age groups compared to the younger age groups, due to the availability of more information in the survey data.

At the state/territory level, variations in declining trends are also observed (Figure 2). We see that over the whole period, the highest smoking prevalence was recorded in NT, while the lowest prevalence was observed in ACT. Overall, examining the trends shows better improvement in terms of the rate of decline in NT. Notwithstanding this, the smoking prevalence still remains relatively high when compared with other Australian states and territories. For the remaining states, however, similar rates of decline are also observed. In the model selection, this is the reason why a linear time trend with a random intercept and slope at the detailed level is found statistically significant instead of a much more complex relationship with time.

The direct estimates at the detailed (state–age–sex) level are highly volatile, as expected, particularly in TAS and NT for the older age groups (Supplementary file Figure S.1). As a result, the error bars of direct estimates are not plotted for visibility purposes. The comparison of the model-based trends with direct estimates at the detailed level confirms that the trends follow the trend of the direct estimator but have the added advantage of providing smoother and more reliable estimates with greater precision. However, for the 25–29 age group (males and females), we do observe stark differences in the detailed level trends in the direct and model-based estimates. For this age group, the direct estimates for males have been reduced dramatically in the survey year 2021 (and have even lower prevalence rates when compared to females). The developed time-series model captures this pattern with more reliable trend estimates.

### Annual percentage changes in the trends of smoking prevalence

**Figure 4:**
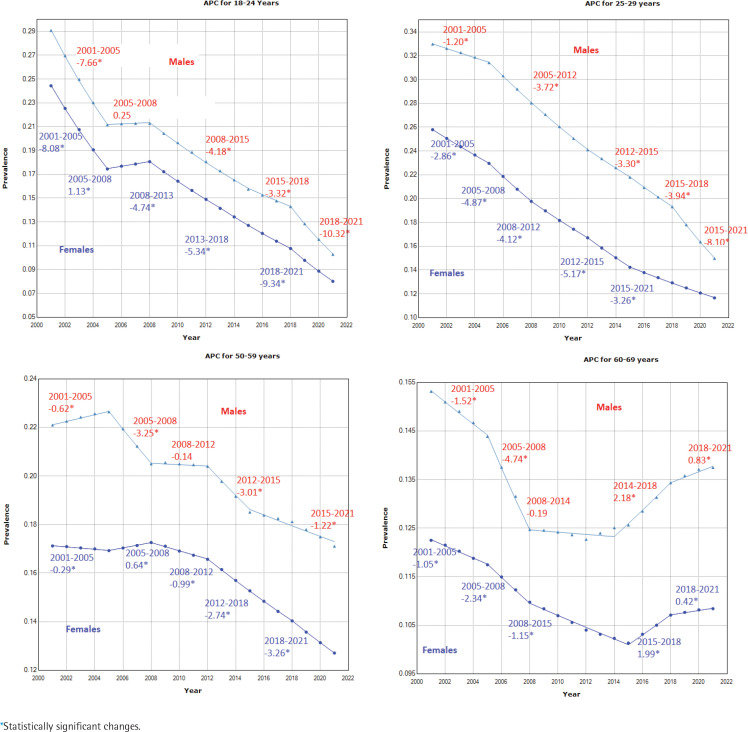
Annual percentage change (APC) in daily smoking prevalence by sex for different age groups, in Australia during 2001–2021. The model-based estimates of daily smoking prevalence for the considered domains during the reference period are used to develop the joinpoint regression models. The APC value obtained from the joinpoint regression model is reported in each segment

The joinpoint trend analysis revealed that there were significant reductions in annual levels of smoking prevalence. At a national level, the average annual percentage change was -3.30 (95% CI: -3.40, -3.20) over the 21-year period (Table 2). The estimated joinpoint model suggests the highest annual percentage change has occurred in the 2018 to 2021 period, which is due to a considerable decline in smoking prevalence among male adults (APC: -6.1 and -4.8 for males and females, respectively). See Supplementary file Figure S.2 for more evidence. These rates of annual decline are more striking when considered by sex and age, with females aged 18–24 years having an AAPC of almost five times (i.e. AAPC = -5.40) larger when compared with males aged 60–69 years (i.e. AAPC = -0.69), (Table 2). For example, we can observe that for males and females aged 18–24 years, there is a very steep decline in 2018–2021 with an APC of -10.32 and -9.38, respectively (Figure 4). In contrast, for older ages, the opposite is true, and the smoking prevalence is rising: for males aged 60–69 years, there is an increase in APC of 0.83, while a smaller but still significant APC of 0.42 is reported for females of the same age, in the 2018–2021 period (Figure 4).

**Table 2:** Average annual percentage change (AAPC) in the daily smoking prevalence in Australia at the national and state level, and according to age, sex, and sex-by-age, 2001–2021

	*Joinpoints*	*AAPC^a^*	*LL^b^*	*UL^c^*	*Test statistic*	*p*
**Geographical level**						
**Country**	4	-3.30	-3.40	-3.30	-109.50	<0.001
**State**						
NSW	4	-3.40	-3.40	-3.30	-74.60	<0.001
VIC	4	-3.40	-3.50	-3.40	-91.50	<0.001
QLD	4	-3.20	-3.20	-3.20	-189.10	<0.001
SA	4	-3.20	-3.30	-3.10	-88.70	<0.001
WA	4	-3.40	-3.40	-3.40	-153.20	<0.001
TAS	4	-2.90	-3.00	-2.80	-62.10	<0.001
NT	4	-3.30	-3.30	-3.20	-170.20	<0.001
ACT	4	-3.60	-3.70	-3.50	-121.00	<0.001
**Demographics**						
**Age** (years)						
18–24	3	-5.20	-5.30	-5.10	-71.30	<0.001
25–29	3	-3.90	-4.00	-3.80	-76.00	<0.001
30–39	4	-3.90	-4.00	-3.80	-82.30	<0.001
40–49	4	-3.80	-4.20	-3.50	-21.00	<0.001
50–59	4	-1.30	-1.60	-1.10	-9.10	<0.001
60–69	4	-0.60	-0.80	-0.40	-6.60	<0.001
≥70	4	-2.10	-2.40	-1.80	-14.40	<0.001
**Sex**						
Female	4	-3.50	-3.60	-3.50	-91.90	<0.001
Male	4	-3.10	-3.20	-3.10	-73.90	<0.001
**Sex-by-age** (years)						
**18–24**						
Female	3	-5.40	-5.50	-5.40	-162.90	<0.001
Male	3	-5.10	-5.20	-4.90	-65.60	<0.001
**25–29**						
Female	4	-3.90	-3.90	-3.80	-126.70	<0.001
Male	4	-3.90	-4.00	-3.80	-78.70	<0.001
**30–39**						
Female	4	-2.80	-2.90	-2.80	-90.50	<0.001
Male	4	-3.40	-3.50	-3.30	-68.20	<0.001
**40–49**						
Female	4	-4.00	-4.10	-3.90	-77.50	<0.001
Male	4	-3.60	-3.80	-3.50	-56.20	<0.001
**50–59**						
Female	4	-1.50	-1.50	-1.50	-118.30	<0.001
Male	4	-1.20	-1.60	-0.80	-5.80	<0.001
**60–69**						
Female	4	-0.60	-0.80	-0.40	-7.40	<0.001
Male	4	-0.50	-0.80	-0.30	-4.30	<0.001
**≥70**						
Female	4	-2.40	-2.60	-2.30	-32.00	<0.001
Male	4	-1.90	-2.20	-1.60	-12.20	<0.001

a AAPC: average annual percentage change.

b LL: lower limit of 95% confidence interval for AAPC.

c UL: upper limit of 95% confidence interval for AAPC. The model-based estimates of daily smoking prevalence for the considered domains during the reference period are used to develop the joinpoint regression models.

The results of APCs for different aggregated domains provided in Table 2 and the APC values shown in Figure 4 for four age groups by sex, indicate that the annual percent change varies significantly over the entire period, and this change is not constant over age, sex, or state/territory level. These show that for both males and females, there are roughly four joinpoint segments from 2000–2005, 2005–2012, 2012–2015, and 2015–2021, characterized by different rates of decline, although there is variability in the lengths and number of joinpoint segments for each of the male and female series for each age group. Considerable differences in APC values by sex are found, and these are more pronounced for the 25–29 years (Figure 4) and 40–49 years (Supplementary file Figure S.3) age groups, especially during the 2018–2021 period. Further, disaggregating by state and territory shows much greater differences in the APC values for the state–age–sex domains. In summary, whilst there is a large amount of nuance in the results presented, it is possible to say that the most striking declines in smoking over the twenty-year period have been seen: in males compared to females; in those aged 18–24 years compared to the older age groups; and in NT compared to the other states and territories. The Supplementary file includes joinpoint regression models and corresponding APC values for various periods across all disaggregated state-age-sex categories.

## DISCUSSION

Smoking prevalence has decreased remarkably in Australia in recent decades. In 2021, roughly one in ten (12%) Australians aged ≥18 years smoked tobacco daily, and this was down from almost a quarter (24%) in 2001. This decline is even steeper when considering its peak in the 1960s when almost three out of every five (59%) adults smoked^34^. However, smoking continues to be the leading risk factor that contributes to the disease burden and deaths in Australia (responsible for almost 10% of all deaths)^9^.

While similar declines in smoking prevalence have been reported in other high-income countries over time, Australia continues to lead the world in reducing the prevalence of smoking amongst its adult population – these decreases are mainly driven by younger generations not taking up smoking^35^. Although these figures are impressive, they can mask localized differences since the smoking prevalence is spatially diverse and distributed unevenly throughout the population^9^.

In fully understanding the patterns and trends in smoking prevalence, there is the need for complete and accurate data at a very detailed level of local geography to identify inequalities so that context-dependent health policy can be planned for improved monitoring and surveillance. Population-based surveys, used for monitoring and surveillance of health indicators such as smoking prevalence, are typically large enough to provide national and state trends but do not have the necessary power to generate detailed risk profiles at a sub-national level^10^. For health-risk factors and outcomes that do have remarkable variation in prevalence amongst the population, for example, smoking, it is imperative that accurate statistics are available by the cross-classification of sex, age, and geography. While it might not be feasible to increase the sample size to generate reliable estimates for various subgroups of the population, small area estimation can be used to provide more reliable granular level estimates by ‘borrowing’ power from other data with more comprehensive coverage and, therefore, artificially increasing the survey sample size. This allows us to obtain a more precise picture of the distribution and localized differences in the prevalence of smoking.

In our study, data from seven National Health Surveys carried out between 2001 and 2021 were analyzed to examine the trends in smoking prevalence. We used a small area modeling approach for obtaining reliable trend estimates of smoking prevalence, incorporating time-series trends and focusing on sub-populations not covered by the survey design, especially in sparsely populated regions of Australia. Providing precise estimates of smoking prevalence sub-nationally is specifically important in identifying geographical differences in order to implement health policy and targeted localized interventions for specific populations. This detailed small area-level information is tremendously important in understanding the factors behind the uneven declining trends in smoking prevalence amongst different sub-populations and geographical communities, and ensuring that there is proper planning and adequate resource allocation at the community level.

The joinpoint analysis of the trend estimates shows that the highest annual percentage change has occurred over the period 2018–2021, particularly for the younger male age groups. The declining pattern in smoking prevalence by sex and age found in the two recent 2016 and 2019 NDSHS surveys support the consistency of the trend estimates obtained in this study^9^. These studies, taken together, show that smoking prevalence has declined more for male adults than for females. Further, the declining rate in smoking prevalence for males aged 25–29 years (from 19.3% in 2016 to 12.7% in 2019) is found to be considerably higher than for females of the same age (from 12.2% in 2016 to 10.1% in 2019) (see Figure 2.2 in the 2019 NDSHS report^9^). This declining pattern is also consistent in this study using the NHS data. In summary, the estimated change in the smoking prevalence obtained from this study is comparable to the results of other studies, though the sampling design and time of the survey may be different.

### Limitations

This study has some limitations related to: 1) the sampling design of the seven surveys; and 2) the utilization of time-invariant covariates in the modeling. To ensure comparability in the surveys over time, it is not possible to change the sampling design to cope with specific situations – for instance, accounting for the likely impact of the COVID-19 pandemic on health outcomes in the NHS 2020–2021 survey. As such, the time-series models do not specifically account for this. Consideration of sampling weights in the estimation of direct estimates (and also the sampling design in variance calculations) is assumed to be sufficient for the comparability of the estimates from different surveys. Since the questionnaire items related to smoking are kept similar in all the surveys, any impacts of the sampling design are assumed to be minimal. However, the assumption that the sampling weights can compensate for changes in smoking prevalence over time in non-responding (young) people might be too strong. Additionally, only time-invariant variables (e.g. sex, age group, and state and territories, as well as their interactions) have been used as covariates in the time-series model. Domain-specific census-based contextual variables (say, the proportion of higher educated adults) can be added, but these time-variant variables will have the same values for at least five years due to the periodicity of the Australian censuses, which occur in a five-year cycle and have been carried out in 2001, 2006, 2011, 2016 and 2021. Finally, when looking at sub-national patterns, smoking rates increase with socioeconomic disadvantage^9^. Additionally, Indigenous Australians are almost three times more likely to smoke when compared to non-Indigenous Australians^36^, acknowledging the fact that smoking rates vary between regions and communities. Due to confidentiality issues (random perturbations are added to small cells to prevent identifiability but can lead to discrepancies for complex cross-classified data), we are unable to have access to data at lower than the state/territory level. However, our study findings can assist health researchers and policymakers in implementing programs tailored to the most vulnerable populations, facilitating the achievement of their health objectives in a timely manner.

## CONCLUSIONS

This study provides model-based trend estimates of the daily smoking prevalence of Australian adults aged ≥18 years by sex, age group, and geographical location, for the last two decades; it illustrates the change patterns in the smoking prevalence at various disaggregation levels by calculating the associated percentage change at different segments of the time-period using joinpoint regression analysis. We find that the performance of the model-based trend estimates outperforms the corresponding design-based direct estimates, in particular in sparse domains (i.e. younger ages and smaller states/territories). The joinpoint analysis shows that there are significant inequalities within and between the disaggregated hierarchies. The trend predictions by sex, along with their joinpoint analysis, indicate that the male–female gap in smoking prevalence has declined in more recent times, particularly among the younger age groups, due to a higher percentage change among male adults. Taken together, this study makes a useful contribution to designing policies that can be targeted at specific sub-groups to reduce smoking prevalence.

## Supplementary Material



## Data Availability

The data are extracted from Australian Bureau of Statistics (ABS) through proper registration. We are not permitted to share the micro-data. The aggregated statistics used in the model development can be shared on reasonable request.

## References

[1] Banks E, Joshy G, Weber MF, et al. Tobacco smoking and all-cause mortality in a large Australian cohort study: findings from a mature epidemic with current low smoking prevalence. BMC Med. 2015;13:38. doi:10.1186/s12916-015-0281-z 25857449 PMC4339244

[2] Australian Bureau of Statistics. National Health Survey methodology. ABS; 2022. Accessed January 22, 2024. https://www.abs.gov.au/methodologies/national-health-survey-methodology/2022

[3] Roche A, McEntee A, Kim S, Chapman J. Changing patterns and prevalence of daily tobacco smoking among Australian workers: 2007-2016. Aust N Z J Public Health. 2021;45(3):290-298. doi:10.1111/1753-6405.13126 34028952

[4] McEntee A, Kim S, Harrison N, Chapman J, Roche A. Patterns and prevalence of daily tobacco smoking in Australia by industry and occupation: 2007-2016. Nicotine Tob Res. 2021;23(12):2047-2055. doi:10.1093/ntr/ntab126 34129034

[5] Hay D. The rise and fall of smoking in New Zealand. J R Coll Physicians Lond. 1993;27(3):315-319. 8377166 PMC5396762

[6] Santiago-Pérez MI, López-Vizcaíno E, Pérez-Ríos M, et al. Small-area models to assess the geographical distribution of tobacco consumption by sex and age in Spain. Tob Induc Dis. 2023;21:63. doi:10.18332/tid/162379 37215189 PMC10194049

[7] Thun MJ, Carter BD, Feskanich D, et al. 50-year trends in smoking-related mortality in the United States. N Engl J Med. 2013;368(4):351-364. doi:10.1056/NEJMsa1211127 23343064 PMC3632080

[8] Australian Bureau of Statistics. National Health Survey: First results. ABS; 2017. Accessed January 22, 2024. https://www.abs.gov.au/statistics/health/health-conditions-and-risks/national-health-survey/2017-18

[9] Australian Institute of Health and Welfare. National Drug Strategy Household Survey 2019. AIHW; 2020. doi:10.25816/e42p-a447

[10] Rao JNK, Molina I. Small Area Estimation. Wiley-Interscience; 2015. doi:10.1002/9781118735855

[11] Boonstra HJ, van den Brakel J, Das S. Multilevel time series modelling of mobility trends in the Netherlands for Small Domains. Journal of the Royal Statistical Society Series A: Statistics in Society. 2021;184(3):985–1007. doi:10.1111/rssa.12700

[12] Das S, van den Brakel J, Boonstra HJ, Haslett S. Multilevel time series modelling of antenatal care coverage in Bangladesh at disaggregated administrative levels. Statistics Canada. 2022;48(2):401–437. Accessed January 22, 2024. http://www.statcan.gc.ca/pub/12-001-x/2022002/article/00010-eng.htm

[13] Clegg LX, Hankey BF, Tiwari R, Feuer EJ, Edwards BK. Estimating average annual per cent change in trend analysis. Stat Med. 2009;28(29):3670-3682. doi:10.1002/sim.3733 19856324 PMC2843083

[14] National Cancer Institute. Joinpoint Trend Analysis Software. Version 4.9.1.0. 2022.

[15] Rahib L, Wehner MR, Matrisian LM, Nead KT. Estimated projection of US cancer incidence and death to 2040. JAMA Netw Open. 2021;4(4):e214708. doi:10.1001/jamanetworkopen.2021.4708 33825840 PMC8027914

[16] Dyvesether SM, Nordentoft M, Forman JL, Erlangsen A. Joinpoint regression analysis of suicides in Denmark during 1980-2015. Dan Med J. 2018;65(4):A5477. 29619927

[17] Das S, Baffour B, Richardson A. Trends in chronic childhood undernutrition in Bangladesh for small domains. Popul Stud (Camb). 2023:30:1-19. doi:10.1080/00324728.2023.2239772 37647268

[18] Australian Bureau of Statistics. 4364.0-National Health Survey: Summary of Results, 2001. ABS; 2002. Accessed January 22, 2024. https://www.ausstats.abs.gov.au/ausstats/subscriber.nsf/0/90A3222FAD5E3563CA256C5D0001FD-9D/$File/43640_2001.pdf

[19] Australian Bureau of Statistics. 4364.0-National Health Survey: Summary of Results, 2004-05. ABS; 2006. Accessed January 22, 2024. https://www.ausstats.abs.gov.au/ausstats/subscriber.nsf/0/3B1917236618A-042CA25711F00185526/$File/43640_2004-05.pdf

[20] Australian Bureau of Statistics. 4364.0-National Health Survey: Summary of Results, 2007-2008. ABS; 2009. Accessed January 22, 2024. https://www.abs.gov.au/ausstats/abs@.nsf/Latestproducts/4364.0Main%20Features42007-2008%20(Reissue)

[21] Australian Bureau of Statistics. 4364.0.55.001-Australian Health Survey: First Results, 2011-12. ABS; 2012. Accessed January 22, 2024. https://www.ausstats.abs.gov.au/ausstats/subscriber.nsf/0/1680ECA402368CCFCA257A-C90015AA4E/$File/4364.0.55.001.pdf

[22] Australian Bureau of Statistics. 4364.0.55.001-National Health Survey: First Results, 2014-15. ABS; 2015. Accessed January 22, 2024. https://www.ausstats.abs.gov.au/ausstats/subscriber.nsf/0/CDA852A349B4CEE6CA257F150009F-C53/$File/national%20health%20survey%20first%20results,%202014-15.pdf

[23] Australian Bureau of Statistics. 4363.0.55.001-National Health Survey: Users’ Guide Electronic, 2007-08. ABS; 2008. Accessed January 22, 2024. https://www.ausstats.abs.gov.au/ausstats/subscriber.nsf/0/CC0FB5A08570984E-CA25762E0017CF2B/$File/4363055001_2007-08.pdf

[24] Liu B, Lahiri P, Kalton G. Hierarchical Bayes modeling of survey-weighted small area proportions. Statistics Canada. 2014;40(1). Accessed January 22, 2024. http://www.asasrms.org/Proceedings/y2007/Files/JSM2007-000547.pdf

[25] Chandra H, Chambers R, Salvati N. Small area estimation of survey weighted counts under aggregated level spatial model. Statistics Canada. 2019;45(1):31–59. Accessed January 22, 2024. https://www150.statcan.gc.ca/n1/pub/12-001-x/2019001/article/00006-eng.htm

[26] Ghosh M. Small area estimation: Its evolution in five decades. Statistics in Transition New Series. 2020;21(4):1–22. doi:10.21307/stattrans-2020-022

[27] R Core Team. R: A Language and Environment for Statistical Computing. R Foundation for Statistical Computing; 2015. Accessed January 22, 2024. https://www.gbif.org/tool/81287/r-a-language-and-environment-for-statistical-computing

[28] Boonstra HJ. mcmcsae: MCMC Small Area Estimation. R package version 0.7.6. 2023. Accessed January 22, 2024. https://cran.r-project.org/package=mcmcsae

[29] Vehtari A, Gabry J, Magnusson M et al. loo: Efficient Leave-One-Out Cross-Validation and WAIC for Bayesian Models. R package version 2.5.1. 2022. Accessed January 22, 2024. https://cran.r-project.org/package=loo

[30] Vehtari A, Gelman A, Gabry J. Practical Bayesian model evaluation using leave-one-out cross-validation and WAIC. Statistics and Computing 2017;27(5):1413–1432. doi:10.1007/s11222-016-9696-4

[31] Gabry J, Simpson D, Vehtari A, Betancourt M, Gelman A. Visualization in Bayesian work-flow. Journal of the Royal Statistical Society Series A: Statistics in Society. 2019;182(2):389–402. doi:10.1111/rssa.12378

[32] Australian Bureau of Statistics. National, state and territory population. ABS; 2023. Accessed January 22, 2024. https://www.abs.gov.au/statistics/people/population/national-state-and-territory-population/latest-release

[33] Guerra-Tort C, López-Vizcaíno E, Santiago-Pérez MI, et al. Validation of a small-area model for estimation of smoking prevalence at a subnational level. Tob Induc Dis. 2023;21:112. doi:10.18332/tid/169683 37664442 PMC10472341

[34] Smith DR, Leggat PA. The historical decline of tobacco smoking among Australian physicians: 1964-1997. Tob Induc Dis. 2008;4(1):13. doi:10.1186/1617-9625-4-13 19114012 PMC2646683

[35] Gartner C, Perusco A, Puljević C, Morphett K, Hefler M. International progress toward a commercial tobacco endgame is an opportunity for advancing tobacco control in Australia. Aust N Z J Public Health. 2023;47(2):100029. doi:10.1016/j.anzjph.2023.100029 36963120

[36] Australian Bureau of Statistics. National Aboriginal and Torres Strait Islander Health Survey. ABS; 2019. Accessed January 22, 2024. https://www.abs.gov.au/statistics/people/aboriginal-and-torres-strait-islander-peoples/national-aboriginal-and-torres-strait-islander-health-survey/latest-release

