# Supplementary material for: Trend estimation of sub-national level daily smoking prevalence by age and sex in Australia
Source: Tob Induc Dis. 2024 Feb 23;22:10.18332/tid/183804. doi: 10.18332/tid/183804 (PMC10885685; doi:10.18332/tid/183804)
Supplement: Supplementary file 1 [file TID-22-45-s1.pdf]

Supplementary File
“Trend estimation of sub-national level daily smoking prevalence by age and sex in Australia”

January 22, 2024

In this supplementary file, we provide detailed descriptions of the methodology and modeling approach, and detailed results of trend estimation of smoking prevalence at different levels of disaggregation as well as the joinpoint regression analysis. Also included are the specific model assessment and validation tests to ascertain the goodness of fit and check the reliability of predictions from the model-based estimation of the prevalence of smoking in Australia. This information is provided to aid researchers interested in additional details necessary for replicating our work. In Section S.1, we discuss the approach used to obtain design-based direct estimates of smoking prevalence from the seven round of National Health Surveys micro-data. In S.2 we provide detailed information about the model development, trend prediction, and model assessment through diagnostics of model parameters and posterior predictive check. The estimated trends of smoking prevalence at various disaggregation levels are shown in Section S.3. In Section S.4, we detail on joinpoint regression analysis of the model-based trend estimates and the relevant results. Finally, we mentioned about which software and R packages are used in this study to conduct the analysis with aim of acknowledging the corresponding institutes and researchers for their valuable scientific contributions. Results are given in detail in this Supplementary file to complement the findings given in the main manuscript.

S.1 Input Estimates

In order to provide details on the model inputs and model development, we define $\hat{y}_{ijkt}$ to be the estimated proportion of adult smokers belonging to age-group i ($i = 1, \ldots, 7$) and sex-group k ($k = 1, 2$) in state and territories j ($j = 1, \ldots, 8$), in year t ($t = 1, \ldots, 21$). The number of sampled adults in domain ijk in survey year t is denoted by n_{ijkt} and the estimated sampling variance of $\hat{y}_{ijkt}$ as $var(\hat{y}_{ijkt})$. The direct estimates, $\hat{y}_{ijkt}$, of daily smoking prevalence and their estimated variances, $var(\hat{y}_{ijkt})$ are computed from the micro-data of seven National Health Surveys (NHS) conducted during 2001-2021 [18, 19, 20, 21, 22, 8, 2]. The survey years are indexed in this study as 2001, 2005, 2008, 2012, 2015, 2018, 2021. For the non-survey years, these inputs are considered as missing. This implies that at the detailed level there are 112 domains which are cross-classification of 8 state and territories and 7 age-groups (18-24, 25-29,

30-39, 40-49, 50-59, 60-69 and 70^+ years), and 2 sex (female and male) groups. So, there are a total of 2352 ($= 21 \times 112$) domains over the entire time period 2001-2021 but with 784 observed and the remaining 1568 are missing.

For a particular survey (say, $t = 2021$, the survey-weighted direct estimates of the daily smoking prevalence at the detailed cross-classified domains are calculated using sampling weights, while their standard errors are estimated using “replication methods” for the complex sample designs and weighting procedures employed in the NHS surveys. Replicate weights are produced in the NHS under the delete-a-group jackknife method of replication [?] and are given in the micro-data. The estimates and the standard errors based on the replication methods are calculated as below:

$$\hat{y}_d = \sum_{l=1}^{n_d} w_{dl} y_{dl} \tag{1}$$

and

$$var(\hat{y}_d) = \frac{59}{60} \sum_{r=1}^{60} (\hat{y}_d^{(r)} - \hat{y}_d)^2 \tag{2}$$

Here, y_{dl} and w_{dl} are the value of target outcome variable (say, 1 for daily smoking and 0 for non-smoking) and the sampling weight for the lth individual of dth $d = 1, \ldots 112$ domain respectively. The replicate version of the survey-weighted direct estimate is $\hat{y}_d^{(r)}, r = 1, \ldots 60$, which is calculated as (1) using r^{th} replicate weights [23]. The number of replicate weights was 30 for the NHS 2001 [18].

The basic microdata (unit record data) of the various NHSs are extracted from the Australian Bureau of Statistics (ABS) [?] except for the 2020-21 round. The microdata of NHS 2020-21 have been accessed through ABS DataLab [?] online platform.

Distributions of the domain-specific sample size, direct estimates of smoking and the corresponding standard errors shown in Table S.1.1 indicate how these statistics are sparse. The number of observed smoker (weighted) can be obtained by multiplying the estimate with the corresponding sample size.

Statistic	Year	Min	Q1	Q2	Mean	Q3	Max	NA
	2001	11.0	81.0	154.5	165.7	220.5	458.0	4
	2004-05	3.0	115.0	177.0	187.2	274.0	442.0	1
	2007-08	1.0	90.8	128.0	140.9	203.0	327.0	-
n	2011-12	24.0	90.8	127.0	138.2	185.5	276.0	-
	2014-15	21.0	78.0	125.5	130.0	180.2	273.0	-
	2017-18	31.0	86.2	125.0	146.2	205.0	383.0	-
	2020-21	10.0	40.0	96.0	90.3	123.2	226.0	-
	2001	3.2	14.6	23.2	22.1	27.7	47.9	4
	2004-05	0.0	14.0	20.4	20.6	27.3	66.7	1
	2007-08	0.0	10.6	19.2	18.3	23.9	50.9	-
est $\times$ 100	2011-12	3.0	11.9	17.6	17.1	21.5	44.0	-
	2014-15	2.4	9.8	14.3	14.8	19.4	33.6	-
	2017-18	2.4	9.9	13.7	14.1	18.5	38.8	-
	2020-21	0.0	5.6	11.2	11.3	15.3	32.3	-
	2001	1.1	2.7	3.5	4.3	4.4	19.6	4
	2004-05	0.0	2.4	3.0	5.1	3.8	47.4	1
	2007-08	0.0	2.6	3.3	4.5	4.6	30.9	-
se $\times$ 100	2011-12	1.3	2.7	3.4	3.7	4.6	8.7	-
	2014-15	1.3	2.6	3.4	3.8	4.6	9.5	-
	2017-18	0.7	2.0	2.6	2.9	3.6	7.4	-
	2020-21	0.0	2.8	4.0	4.7	5.7	18.1	-

Table S.1.1: Distribution of the number of observed (n), the direct estimate of smoking (est) and the corresponding standard error (se) for the domains cross-classified by 8 state and territories, two sexes and 7 age-groups in Australia estimated from micro-data of National Health Surveys 2001, 2004-05,2007-08, 2011-12, 2014-15, 2017-18, 2020-21.

Variable	NHS	2001	2004-05	2007-08	2011-12	2014-15	2017-18	2020-21
Sex	Female	9749	11218	8341	8228	7907	8794	5482
	Male	8142	9562	7438	7247	6653	7576	4634
Age	18-24	1628	3092	1363	1226	1125	1174	523
	25-29	1624	1478	1256	1299	1139	1145	618
	30-39	3871	3875	3084	2883	2693	2898	1791
	40-49	3741	3892	3057	2987	2666	2829	1814
	50-59	2686	3252	2596	2638	2486	2751	1781
	60-69	1956	2404	2221	2300	2286	2679	1890
	70^+	2385	2787	2202	2142	2165	2894	1699
State	NSW	3952	4264	3034	2722	2464	3270	2039
	VIC	3713	3584	2594	2500	2522	2611	1515
	QLD	3142	3359	2410	2444	2348	3362	1336
	SA	2092	3676	2440	1954	1900	1658	1228
	WA	2204	2258	1851	2135	1828	1655	1332
	TAS	1166	2071	1514	1464	1482	1605	1210
	NT	235	119	111	964	753	1088	457
	ACT	1387	1449	1825	1292	1263	1121	999
Sex & Age	Female 18-24	869	1569	679	600	565	612	281
	Female 25-29	909	797	656	721	607	591	329
	Female 30-39	2104	2090	1670	1549	1530	1572	981
	Female 40-49	1990	2033	1552	1510	1442	1492	1001
	Female 50-59	1373	1717	1327	1389	1327	1459	981
	Female 60-69	1068	1269	1137	1236	1229	1406	1036
	Female 70^+	1436	1743	1320	1223	1207	1662	873
	Male 18-24	759	1523	684	626	560	562	242
	Male 25-29	715	681	600	578	532	554	289
	Male 30-39	1767	1785	1414	1334	1163	1326	810
	Male 40-49	1751	1859	1505	1477	1224	1337	813
	Male 50-59	1313	1535	1269	1249	1159	1292	800
	Male 60-69	888	1135	1084	1064	1057	1273	854
	Male 70^+	949	1044	882	919	958	1232	826
Sex & State	Female NSW	2133	2327	1630	1450	1367	1791	1141
	Female VIC	2098	1987	1353	1317	1366	1372	820
	Female QLD	1690	1786	1258	1289	1289	1796	731
	Female SA	1120	1957	1300	1040	1013	881	654
	Female WA	1171	1212	958	1122	999	870	707
	Female TAS	657	1117	778	798	818	874	654
	Female NT	134	54	65	508	384	565	235
	Female ACT	746	778	999	704	671	645	540
	Male NSW	1819	1937	1404	1272	1097	1479	898
	Male VIC	1615	1597	1241	1183	1156	1239	695
	Male QLD	1452	1573	1152	1155	1059	1566	605
	Male SA	972	1719	1140	914	887	777	574
	Male WA	1033	1046	893	1013	829	785	625
	Male TAS	509	954	736	666	664	731	556
	Male NT	101	65	46	456	369	523	222
	Male ACT	641	671	826	588	592	476	459
	Total	17891	20780	15779	15475	14560	16370	10116

Table S.1.2: The number of participants by age, sex, state and territories in the National Health Surveys 2001, 2004-05,2007-08, 2011-12, 2014-15, 2017-18, 2020-21.

Variable	Levels	Survey	2001	2005	2008	2012	2015	2018	2021
Age	18-24	Female	53.4	50.7	49.8	48.9	50.2	52.1	53.7
		Male	46.6	49.3	50.2	51.1	49.8	47.9	46.3
	25-29	Female	56.0	53.9	52.2	55.5	53.3	51.6	53.2
		Male	44.0	46.1	47.8	44.5	46.7	48.4	46.8
	30-39	Female	54.4	53.9	54.2	53.7	56.8	54.2	54.8
		Male	45.6	46.1	45.8	46.3	43.2	45.8	45.2
	40-49	Female	53.2	52.2	50.8	50.6	54.1	52.7	55.2
		Male	46.8	47.8	49.2	49.4	45.9	47.3	44.8
	50-59	Female	51.1	52.8	51.1	52.7	53.4	53.0	55.1
		Male	48.9	47.2	48.9	47.3	46.6	47.0	44.9
	60-69	Female	54.6	52.8	51.2	53.7	53.8	52.5	54.8
		Male	45.4	47.2	48.8	46.3	46.2	47.5	45.2
	70^+	Female	60.2	62.5	59.9	57.1	55.8	57.4	51.4
		Male	39.8	37.5	40.1	42.9	44.2	42.6	48.6
State	NSW	Female	54.0	54.6	53.7	53.3	55.5	54.8	56.0
		Male	46.0	45.4	46.3	46.7	44.5	45.2	44.0
	VIC	Female	56.5	55.4	52.2	52.7	54.2	52.5	54.1
		Male	43.5	44.6	47.8	47.3	45.8	47.5	45.9
	QLD	Female	53.8	53.2	52.2	52.7	54.9	53.4	54.7
		Male	46.2	46.8	47.8	47.3	45.1	46.6	45.3
	SA	Female	53.5	53.2	53.3	53.2	53.3	53.1	53.3
		Male	46.5	46.8	46.7	46.8	46.7	46.9	46.7
	WA	Female	53.1	53.7	51.8	52.6	54.6	52.6	53.1
		Male	46.9	46.3	48.2	47.4	45.4	47.4	46.9
	TAS	Female	57.0	45.4	58.6	52.7	51.0	51.9	51.4
		Male	43.0	54.6	41.4	47.3	49.0	48.1	48.6
	NT	Female	56.3	53.9	51.4	54.5	55.2	54.5	54.0
		Male	43.7	46.1	48.6	45.5	44.8	45.5	46.0
	ACT	Female	53.8	53.7	54.7	54.5	53.1	57.5	54.1
		Male	46.2	46.3	45.3	45.5	46.9	42.5	45.9
Overall		Female	54.5	54.0	52.9	53.2	54.3	53.7	54.2
		Male	45.5	46.0	47.1	46.8	45.7	46.3	45.8

Table S.1.3: Sex distribution by age and state in the National Health Surveys 2001, 2004-05, 2007-08, 2011-12, 2014-15, 2017-18, 2020-21.

S.2 Model development, trend prediction and model assessment

To define the multilevel time-series model for the outcome values, let $\hat{y}_{dt}$ denote the number of smokers (or daily smoking prevalence) for domain d and year t. The index d runs over 1 to $M_d = 112$, and t over 1 to $T = 21$ years. Direct estimates $\hat{y}_{dt}$ are combined in a large vector of dimension $M = M_d T$ as $\hat{\boldsymbol{y}} = (\hat{y}_{11}, \ldots \hat{y}_{M_{d1}}, \ldots \hat{y}_{1T}, \ldots \hat{y}_{M_{dT}})'$ to define a hierarchical Bayesian multilevel time-series model for $\hat{\boldsymbol{y}}$ as the general linear additive form

$$\hat{\boldsymbol{y}} \sim f(\boldsymbol{\mu}, \boldsymbol{\phi}) \quad ; \quad g(\boldsymbol{\mu}) = \boldsymbol{\eta} = \boldsymbol{X\beta} + \sum_{\alpha} \boldsymbol{Z}^{(\alpha)} \boldsymbol{v}^{(\alpha)} \,, \tag{3}$$

where $f(.)$ is a probability distribution depending on the vector of $\hat{\boldsymbol{y}}$ with an optional scale or dispersion parameter ϕ and $g(.)$ is a link function that links the mean vector to the linear predictor $\boldsymbol{\eta}$, $\boldsymbol{X}$ is a $M \times p$ design matrix for a p-vector of fixed effects $\boldsymbol{\beta}$, and the $\boldsymbol{Z}^{(\alpha)}$ are $M \times q^{(\alpha)}$ design matrices for $q^{(\alpha)}$-dimensional random effect vectors $\boldsymbol{v}^{(\alpha)}$. If $\hat{\boldsymbol{y}}$ consists of count values, we assume $f(.)$ is a binomial distribution with mean vector $\boldsymbol{\mu}$ and logistic link function as $g(\boldsymbol{\mu}) = log\frac{\boldsymbol{\mu}}{(1-\boldsymbol{\mu})}$; if $f(.)$ is a normal distribution then the link function $g(.)$ will be an identity function. The term summed over α indicates that several possible random effects terms at different levels (e.g., local level and smooth trends at state/territory levels) can be added in the model. More detail on these random effects terms can be found in [11, 12, 17].

A vector of random effects under a particular $\boldsymbol{v}^{(\alpha)}$ component can be assumed to be distributed as

$$\boldsymbol{v} \sim \mathcal{N}(0, \boldsymbol{A} \otimes \boldsymbol{V}) \,, \tag{4}$$

where $\boldsymbol{V}$ and $\boldsymbol{A}$ are respectively $m \times m$ and $l \times l$ covariance matrices, and the Kronecker product of $\boldsymbol{A}$ with $\boldsymbol{V}$ $(\boldsymbol{A} \otimes \boldsymbol{V})$ indicates the total length of $\boldsymbol{v}$ is $q = ml$. As for example, assuming age level effects $(d = 7)$ varying over time $(l = 21)$ consists of $q = 147$ random effects [?]. The covariance matrix $\boldsymbol{A}$ describes the covariance structure among the levels of the factor variable, and is assumed to be known. Instead of covariance matrices, precision matrices $\boldsymbol{Q_A} = \boldsymbol{A}^{-1}$ are actually used, because of computational efficiency [?]. The covariance matrix $\boldsymbol{V}$ for the d varying effects can be parameterized in three different forms: (i) an unstructured, i.e. fully parameterized covariance matrix, (ii) a diagonal matrix with unequal diagonal elements, and (iii) a diagonal matrix with equal diagonal elements. The standard deviation parameters are assumed to follow the scaled-inverse Wishart and half-Cauchy priors when unstructured [?] and diagonal [?] covariance structure are assumed respectively.

To examine the convergence of the Markov Chain Monte Carlo (MCMC) simulation, we use trace and autocorrelation plots as well as the Gelman-Rubin potential scale reduction factor [?], which diagnoses the mixing of the chains. These plots are shown in sub-section S.2.3. Finally, the MTS models are developed using 1000 burn-in and subsequently 10000 iterations of which the draws of every fifth iteration are stored and consequently, $3 \times 2000 = 6000$ draws are used to compute estimates and standard errors. Longer simulations of the selected model provide Gelman-Rubin potential scale reduction factor [?] below 1.05 and sufficient effective numbers

of independent draws for all model parameters and model predictions.

S.2.1 Model development

The main outcome variable of interest is the proportion of adult smokers in Australia over time and geo-demographic domains. However, the time-series model in this study has been developed using the observed number of adult smokers for the considered domains as the response variable, which is assumed to follow a binomial distribution with the estimated proportion of smokers as the mean and the number of observed adults as the number of cases for a particular domain. For developing the time-series model, a standardized form of the integer *year* variable denoted as *year.s* has been used in the model as fixed effect. This helps to model linear time trend of the outcome variable. The domain classification variables *State* (state and territories: ACT as the reference), *age* (age-group: 18-24 as the reference) and *sex* (female as the reference) as well as their interaction effects are examined as fixed effects component. The finally selected model suggests to keep both main and interaction effects of age and sex as the fixed effects component. Two types of fixed effects components (FE_1 and FE_2) expressed as in (5) have been examined in the model development stage.

$$\begin{aligned} FE_1 =& 1 + year.s + State + Age + Sex + Age \times Sex \\ FE_2 =& 1 + year.s + State + Age + Sex \end{aligned} \tag{5}$$

Here the term $Age \times Sex$ indicates interaction effects of age and sex. Comparison of FE_1 and FE_2 helps to examine whether interaction effects of age and sex are importnat or not.

We include random effects components to capture the unobserved heterogeneity in smoking over time and geo-demographic domains. We borrow strength across similar domains in different ways to produce reliable estimates of the underlying rate of smoking, rather than using the observed sparse data. To borrow strength over cross-sectional domains, the matrix V in (4) can be structured either as a full covariance matrix or a diagonal matrix with equal or unequal elements. A full covariance structure allows the modeling of the correlation among the different random effects, implying that heterogeneity changes over domains. To borrow temporal strength, we require an appropriate correlation structure. Since we have repeated observations over time, we can model the underlying time-series structure through a random walk (RW) which imposes a certain level of smoothness in the data over time, assuming that future behaviour is independent of the past history, but does not make any distributional assumptions.

After extensive examination of various random effects components at various disaggregation levels, two random effects components *RIS* and *RW_1* shown in Table S.2.1 are found to have significant contributions in developing the time-series model. The component *RIS* assumes random intercept and random slope varying over the $M_d = 112$ detailed level cross-classified domains of state, age and sex. It is assumed that random intercept and slope terms have unequal (diagonal) variance components, denoted as σ_I^2 and σ_S^2 respectively. Since the intercept and slopes vary over the geo-demographic domains, they capture the variability due to demographic characteristics as well as their geographic location.

The examination of the (predicted) trend components at various aggregation levels (such as by age and sex) suggest that the variability over time is considerable only for the age-sex cross-classified domains. Two temporal components shown in Table S.2.1 denoted by *RW1_1* and

RW1_1 are examined. Both these $RW1$ components help to model local-level trends (i.e., the current value depends more on it's immediate time point value), but in a slightly different way. The first component *RW1_1* is simpler than the second one, though both have same number of parameters and random effects. The former one assumes age-sex specific trends varying over the levels of $year$, while the second one assume only age-specific trends varying over the level of $year * sex$. Since the former one consider trends at more dis-aggregated level than the later one, the first one accounts higher variability and hence provide higher standard error of the model-based estimates.

We have also examined spatial random effects for the state and territories through a conditional autoregressive (CAR) model [**?**]. These CAR models make stronger distributional assumptions about the relationship of smoking prevalence in different domains. For details of specification of these spatial random effect terms in spatio-temporal models, please see [**? ? ?**]. Spatial random effects components recognise that we can borrow spatial strength (over state and territories) because neighbouring geographic areas are more likely to share similar characteristics, through spatial dependence. Since there are only 8 geographic domains, such spatial-effects components are found to have negligible contribution after the RIS component, unfortunately. For this reason, state and territory level spatial effects are not considered in the final model selection.

Model Component	Formula **V**	Variance Structure	Factor **A**	Parameters	Number of Effects $(m \times l)$
RIS	$1 + year.s$	diagonal	$State * Age * Sex$	2	$2 \times 112 = 224$
RW1_1	$Age * Sex$	scalar	RW1(year)	1	$(7 \times 2) \times 21 = 294$
RW1_2	Age	scalar	$RW1(year) * Sex$	1	$7 \times (21 \times 2) = 294$

Table S.2.1: Summary of the random effect components for the considered multilevel time-series model for smoking. The second and third columns refer to the varying effects with covariance matrix V in (4), whereas the fourth column refers to the factor variable associated with A in (4). The last two columns contain the total number of parameters and random effects for each random effects component.

A number of MTS models of the form (3) are developed using combinations of fixed effects components (5) and random effects components shown in Table S.2.1. A model denoted by MTS-1 shown in Table S.2.2 was first developed by considering random intercept and slope terms *RIS* and a random walk *RW1_1* which bring (a) cross-sectional, spatial (unstructured) strength and (b) temporal strength among the detailed level state-age-sex domains. To understand the benefit of the interaction effects of age and sex, a new model MTS-2 has been fitted using fixed effects (with interactions) FE_2 instead of FE_1. Since age-level variability is considerably higher (nosier) than at state and sex level, only the age-specific trend is expected to work better to obtain smoothed trend estimates. So, next model MTS-3 is fitted by replacing *RW1_1* by *RW1_2* in model MTS-2, to provide more stable age-sex estimates.

The comparison of the fitted models in terms of information criteria (DIC, WAIC1 and WAIC2) indicate that MTS-1 and MTS-3 perform similarly - MTS-1 has fewer number of parameters. On the other hand, the comparison of MTS-1 and MTS-2 confirms the necessity of including interaction effects of age and sex in MTS-1 model. The differences between MTS-1 and MTS-3 are due to the specification of the random walk terms $RW1_1$ and $RW1_2$ in addition to

Model	FE	RE	DIC	WAIC1	WAIC2	LOOIC	ELPD	parameters
MTS-1	FE_1	RIS+RW1_1	4469	4458	4489	4495	-2247	$22+3$ $=25$
MTS-2	FE_2	RIS+RW1_1	4473	4461	4492	4500	-2250	$16+3$ $=19$
MTS-3	FE_2	RIS+RW1_2	4469	4455	4490	4498	-2249	$16+3$ $=19$

Table S.2.2: Fitted multilevel time-series models with a same FE component but various RE components along with the information criteria (DIC and WAIC values) and number estimated (fixed and random effects) parameters.

Model	MTS-1		MTS-2		MTS-3	
	Mean	t-value	Mean	t-value	Mean	t-value
Fixed effects parameters						
(Intercept)	-2.03	-34.79	-2.07	-41.45	-2.07	-41.51
Sex: Male	0.24	3.80	0.32	12.72	0.32	14.15
Age: 25-29	0.20	2.95	0.29	5.93	0.30	6.16
Age: 30-39	0.15	2.55	0.25	5.36	0.25	5.74
Age: 40-49	0.19	3.18	0.22	4.75	0.22	5.09
Age: 50-59	0.07	1.13	0.09	1.97	0.09	2.03
Age: 60-69	-0.35	-5.47	-0.37	-7.61	-0.37	-7.99
Age: 70+	-1.21	-16.77	-1.20	-21.81	-1.19	-22.97
State: NSW	0.26	6.31	0.26	6.07	0.26	6.21
State: NT	0.65	11.91	0.65	11.15	0.65	11.45
State: QLD	0.38	9.53	0.38	9.01	0.38	9.01
State: SA	0.31	7.37	0.31	6.95	0.31	6.94
State: TAS	0.52	11.55	0.52	11.34	0.52	11.13
State: VIC	0.25	6.00	0.25	5.89	0.25	5.86
State: WA	0.22	5.03	0.22	4.86	0.22	4.94
year.std	-0.23	-5.91	-0.23	-5.93	-0.22	-6.40
Sex: Male × Age: 25-29	0.19	2.21				
Sex: Male × Age: 30-39	0.20	2.57				
Sex: Male × Age: 40-49	0.06	0.78				
Sex: Male × Age: 50-59	0.04	0.54				
Sex: Male × Age: 60-69	-0.04	-0.47				
Sex: Male × Age: 70+	0.03	0.30				
Random effects parameters						
$\hat{\sigma}_I^2$	0.04	2.27	0.05	3.33	0.05	2.89
$\hat{\sigma}_S^2$	0.04	2.88	0.05	2.99	0.05	2.93
$\hat{\sigma}_{RW1}^2$	0.07	6.88	0.07	7.26	0.09	7.80

Table S.2.3: Estimated fixed effects and random effects (i.e., regression coefficients and variance components) parameters along with their t-values in the developed multilevel time-series models MTS-1, MTS-2 and MTS-3

the regression coefficients related to the interaction effects of age and sex. Due to having extra parameters, the MTS-1 model is expected to provide higher variability for the target parameters (smoking prevalence) than for MTS-3 model (which is more parsimonious). So, the final model is selected based by examining how the trend predictions at the aggregated level perform. These comparison are discussed in the following sub-sections.

S.2.2 Trend Prediction

After developing the model, the trend estimates of smoking prevalence at various aggregation levels were computed based on the MCMC simulation results. The simulation vectors of linear predictions based on the fitted model are formed as

$$\boldsymbol{\eta}^{(s)} = \boldsymbol{X}\hat{\boldsymbol{\beta}}^{(s)} + \sum_{\alpha} \boldsymbol{Z}^{(\alpha)}\boldsymbol{v}^{(\alpha,s)}\,, \tag{6}$$

where superscript (s) indexes the simulated MCMC draws for the detailed level state-age-sex domains for all years and so each $\boldsymbol{\eta}^{(s)}$ is of dimension M. The means and the standard deviations over the MCMC draws are used as trend estimates and standard error estimates respectively at the most detailed level. The trends at the most detailed level (state-age-sex) were then aggregated to obtain trends at the national, state, age, sex, state-sex, and age-sex levels. Aggregations at higher level are the weighted average of the estimated detailed level smoking prevalence using the total number of adults aged 18 years and above for the target domains obtained from the quarterly population estimates calculated by the Australian Bureau of Statistics under Estimated Resident Population (ERP) program [32].

The national level trend obtained from the three model-based estimators are plotted in Figure S.2.1 along with the survey design-based estimator. Model estimators behave very similar with some exception at the end of the time period. The MTS-3 estimator provides slightly better trend as per the direct estimates in 2018 and 2021. This estimator also provide the lowest confidence band over the whole time period, while MTS-1 and MTS-2 estimators provide higher confidence band in the non-survey years.

The sex-level trends shown in Figure S.2.2 indicate the differences in the three model-based estimators are mainly in the male trend estimates during 2018-2021. When we look at the confidence band, this is also clear that there are no considerable difference among the MTS estimators for the female trends, while MTS-1 and MTS-2 provides higher confidence for the male trends over the whole time period.

The age-level trends shown in Figure S.2.3 indicate that the considerable differences are among younger age-groups 18-24 and 25-29 years. For these two age-groups, the MTS-1 model-based trends provide slightly higher estimates in the 2021 survey year compared to others. The differences in the confidence band are not considerable among the model-based estimators.

The state-level trends shown in Figure S.2.4 do not show considerable differences among the three estimators except the MTS-1 model-based estimator shows slightly higher confidence band.

The differences among the three estimators are more apparent when we examine the trends by age and sex. Figure S.2.5 shows that MTS-3 model-based estimator follows the trend of direct estimates during 2018-2021. In 2020-21 NHS, the direct estimate for 25-29 years male adults is found considerably smaller than the females. The MTS-3 model follows this trend and shows there was no change in the trends during 2018-2021. But the MTS-1 and MTS-2 model-based estimators seem to provide reasonable trends for the female 25-29 years group by avoiding the pattern of direct estimates. There is evidence that for that period, the NHS sample did not

adequately capture the declining trends in smoking prevalence (particularly amongst young people). While weighting can ameliorate for this, it cannot compensate for changing behaviour of non-responding males. For this reason, it would appear that there is higher prevalence of smoking among young females (when compared with young males) - however this is not true [?]. The MTS-1 and MTS-2 models depend more on the age-sex level trends, while MTS-3 model depends only on the age-level trend and so MTS-3 fails to capture the difference in male-female overall trends. Since MTS-3 model does not account the sex-wise difference in the trends, it provides lower confidence band compared to the MTS-1 and MTS-2 model-based estimator. In addition, for the 18-24 and 40-49 years group,s the MTS-1 and MTS-2 model also provide different trends when compared to the MTS-3 model, especially in the 2018-2021 period. As such, the MTS-1 and MTS-2, are preferred.

At the detailed level, the differences among the three model-based estimators are more explicit for the 25-29 years when we look at them by state in Figure S.2.7. Here we can see that model MTS-1 performs better than the other models. For the other age-groups shown in Figures S.2.13-S.2.8, the differences are not considerable, however MTS-3 model follows direct estimates even at the detailed level (due to its inability to follow the age-sex overall trends). In this sense, MTS-1 model seems more robust than the MTS-3 model due to being less influenced by the inconsistencies in the direct estimates.

Based on the above discussion, on the one hand, the estimator based on MTS-3 model seems better in terms of accuracy of the estimates but this estimator provides unrealistic trends for male and female trends (especially during 2018-2021) due to having tendency to follow the direct estimates. While, on the other hand, MTS-1 and MTS-2 model-based estimators provide reasonable trend estimates for all the considered aggregated levels but with slightly higher confidence bands due to accounting for additional variation in the trends of male adults. Since MTS-1 model is better than MTS-2 in terms of performance criteria (DIC, and WAIC), the estimator based on MTS-1 model has been considered for the trend estimates of smoking prevalence at the detailed state-age-sex level in Australia. For the results presented in the paper, we select MTS-1 for inferences about the trend estimation of sub-national smoking prevalence in Australia.

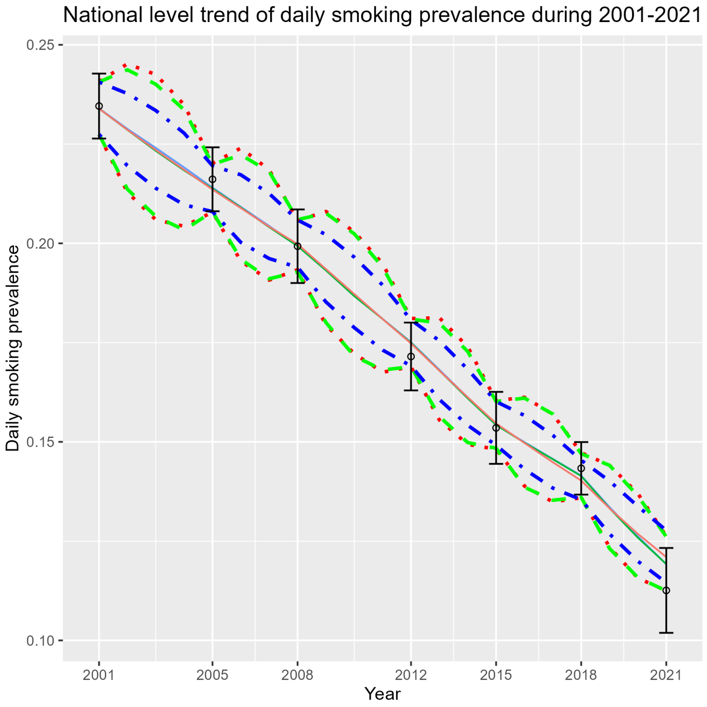

Figure S.2.1: National level trends in daily smoking among adults aged 18 years and above in Australia during 2001-2021 estimated by DIR estimator (black error-bar line), MTS-1 model (red line), MTS-2 model (green line) and MTS-3 model (blue line) with 95 per cent credible band.

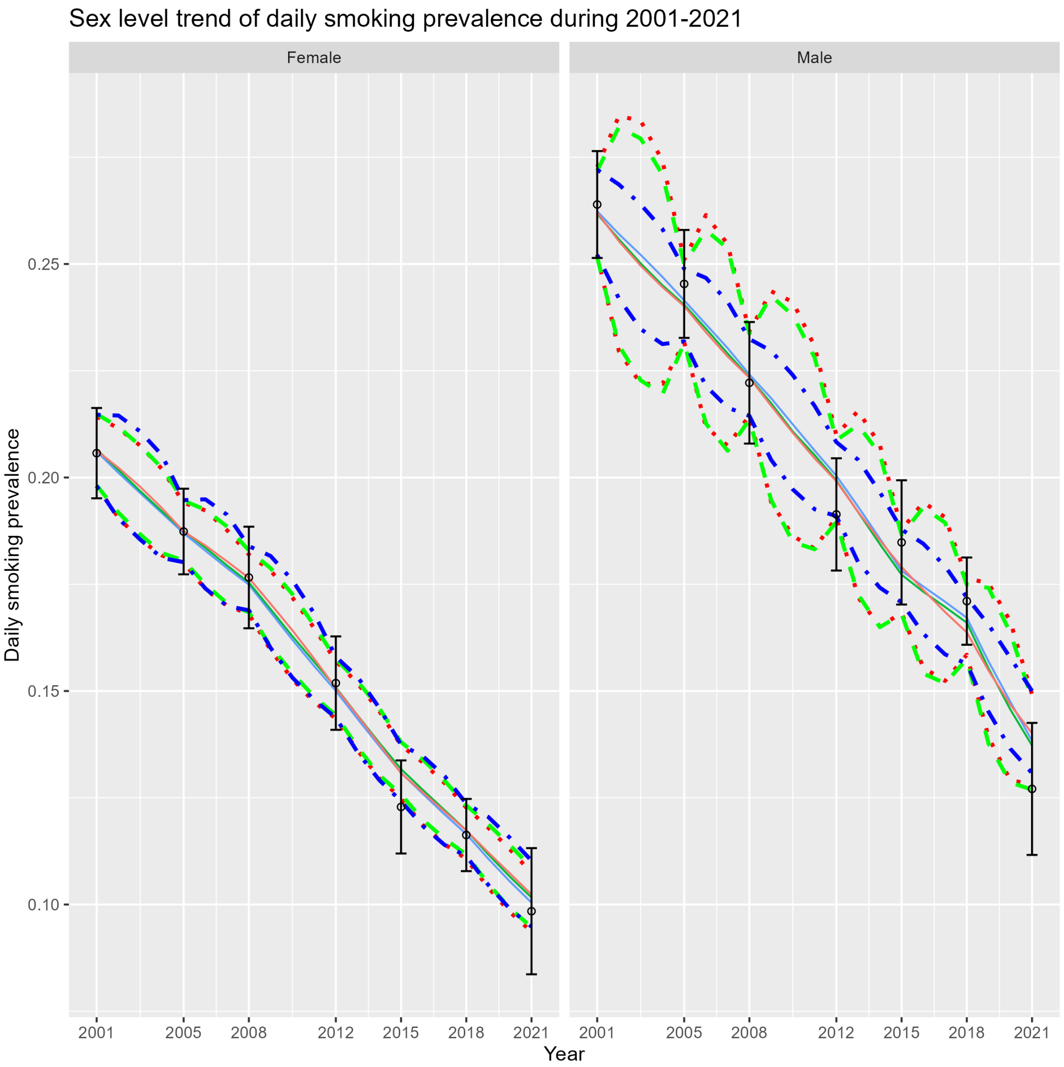

Figure S.2.2: Sex level trends in daily smoking among adults aged 18 years and above in Australia during 2001-2021 estimated by DIR estimator (black error-bar line), MTS-1 model (red line), MTS-2 model (green line) and MTS-3 model (blue line) with 95 per cent credible band.

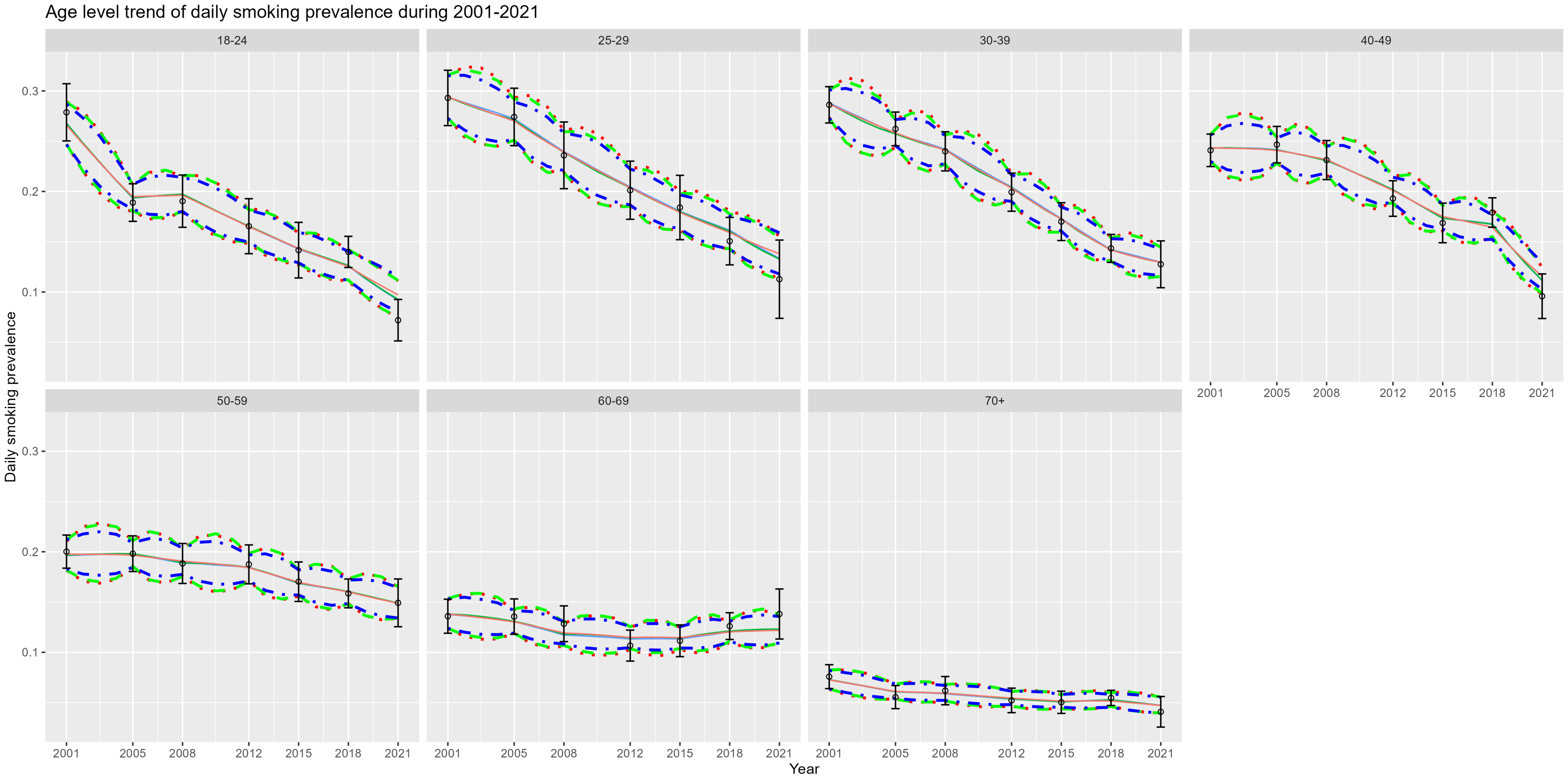

Figure S.2.3: Age level trends in daily smoking among adults aged 18 years and above in Australia during 2001-2021 estimated by DIR estimator (black error-bar line), MTS-1 model (red line), MTS-2 model (green line) and MTS-3 model (blue line) with 95 per cent credible band.

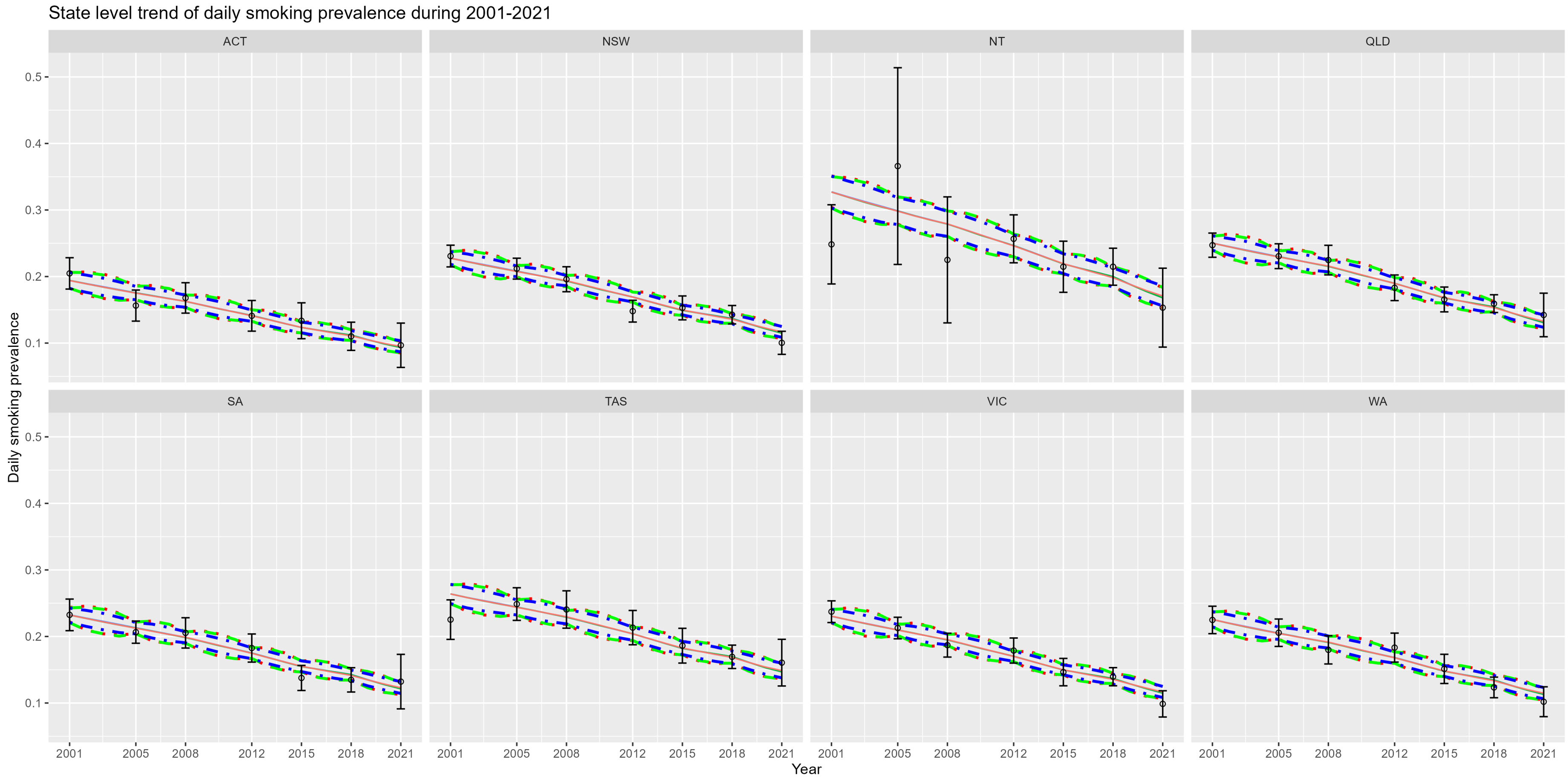

Figure S.2.4: State level trends in daily smoking among adults aged 18 years and above in Australia during 2001-2021 estimated by DIR estimator (black error-bar line), MTS-1 model (red line), MTS-2 model (green line) and MTS-3 model (blue line) with 95 per cent credible band.

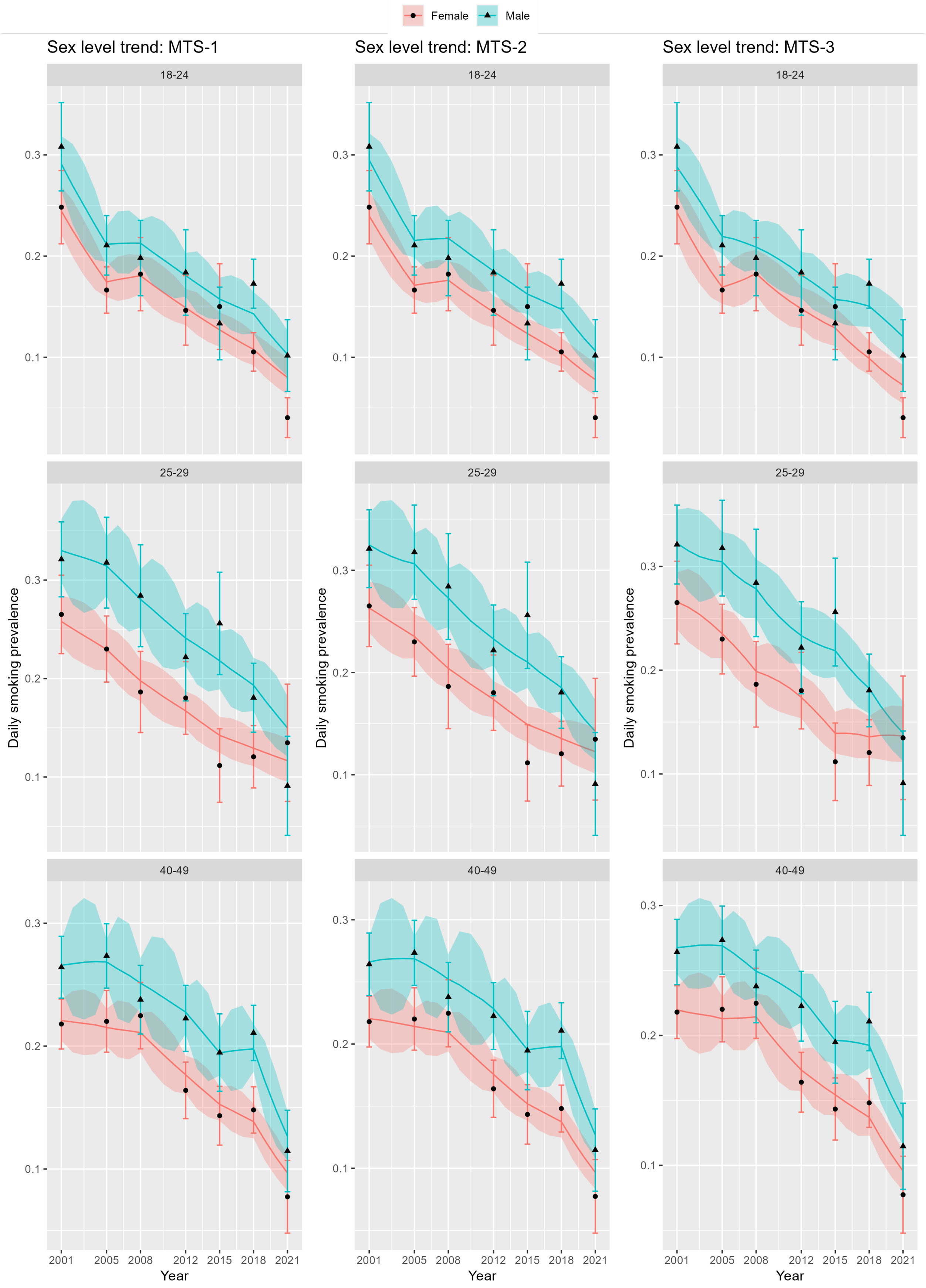

Figure S.2.5: Age-sex level trends in daily smoking among adults aged 18-24, 25-29, and 40-49 years in Australia during 2001-2021 estimated by DIR estimator (black error-bar line), and three model-based (MTS-1, MTS-2 and MTS-3) estimators with 95 per cent credible band.

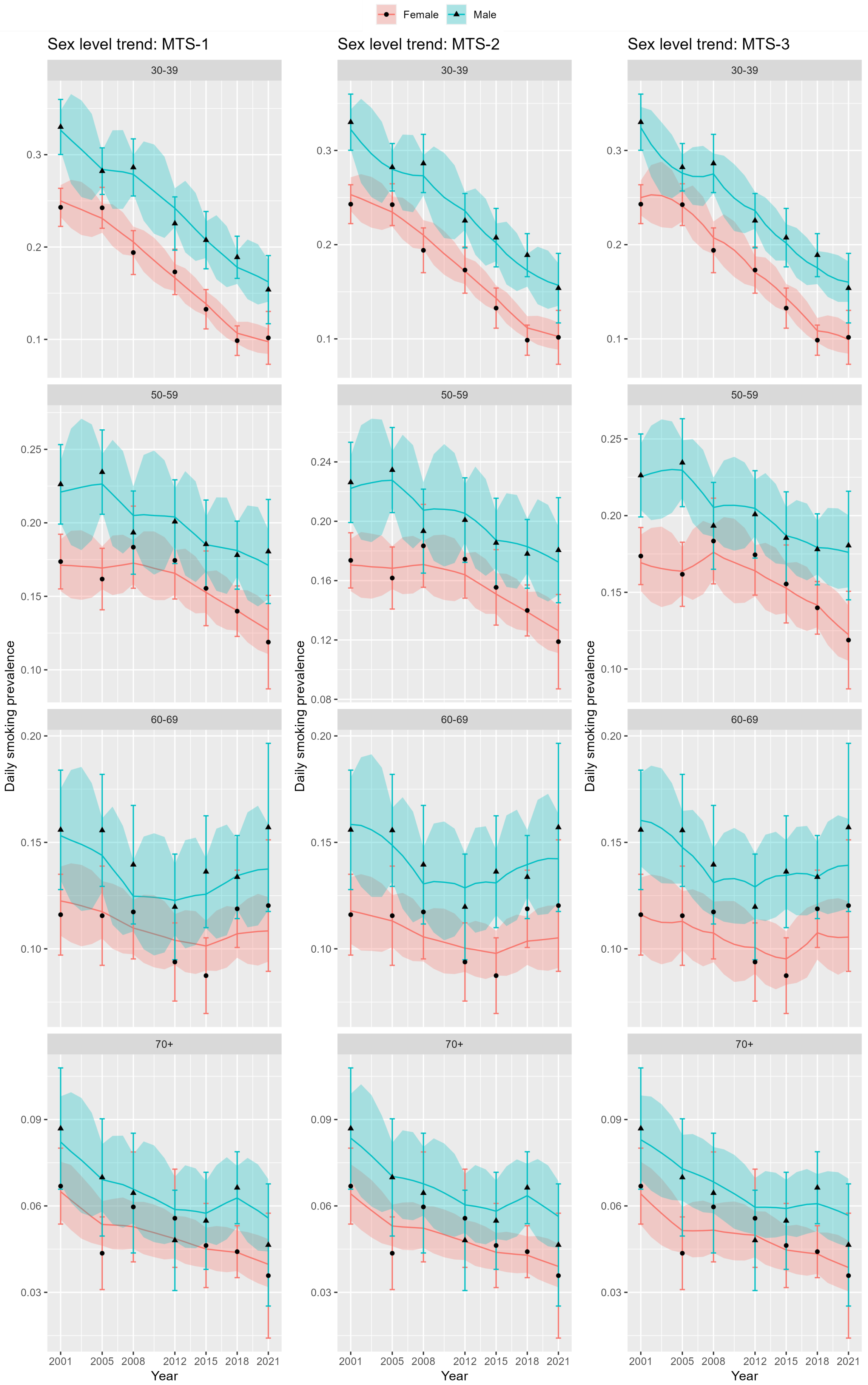

Figure S.2.6: Age-sex level trends in daily smoking among adults aged 30-39, 50-59, 60-69, and 70^{+} years in Australia during 2001-2021 estimated by DIR estimator (black error-bar line), and three model-based (MTS-1, MTS-2 and MTS-3) estimators with 95 per cent credible band.

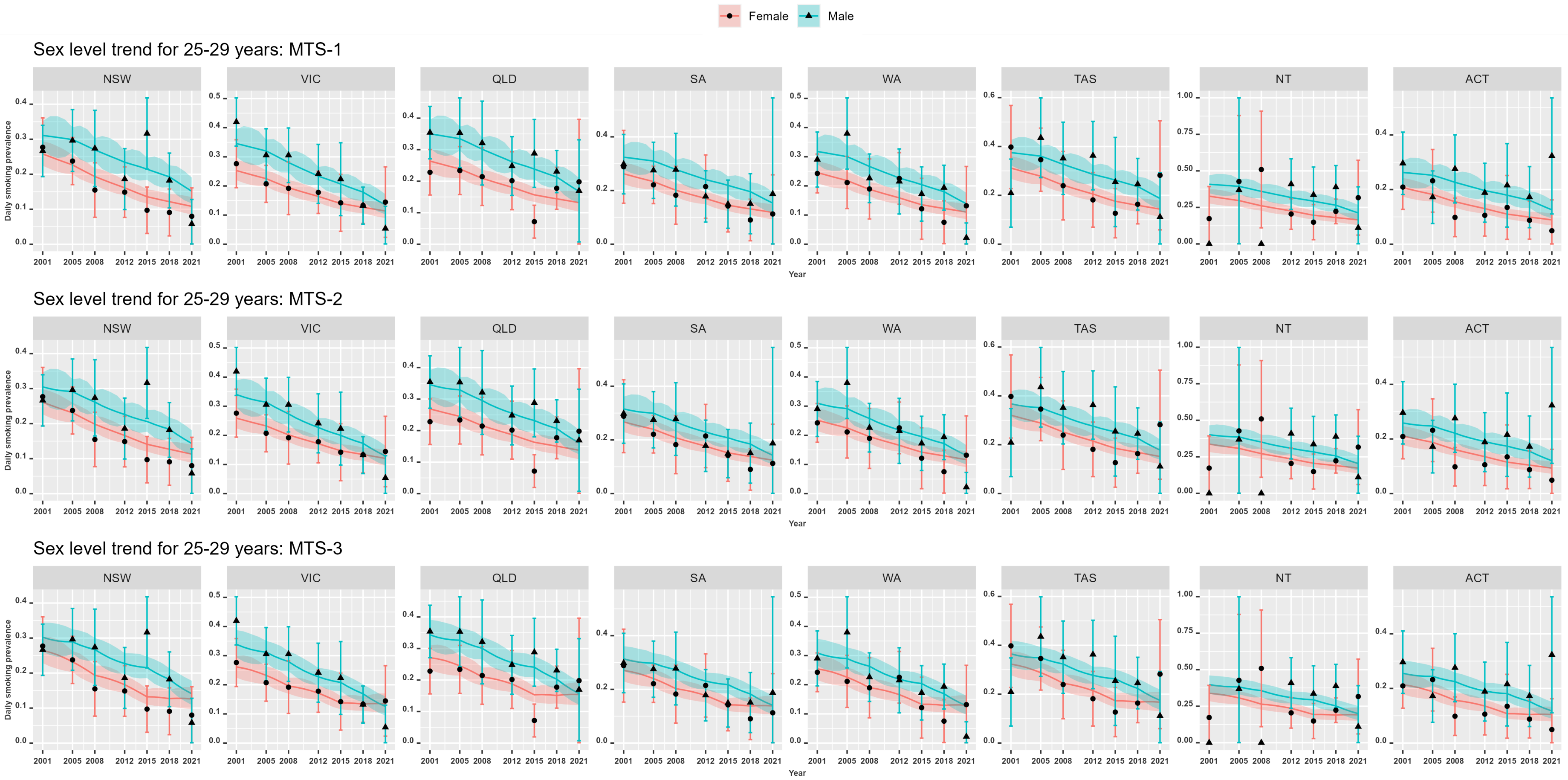

Figure S.2.7: Sex-by-state level trends in daily smoking among adults aged 25-29 years in Australia during 2001-2021 estimated by DIR estimator (black error-bar line), and three model-based (MTS-1, MTS-2 and MTS-3) estimators with 95 per cent credible band.

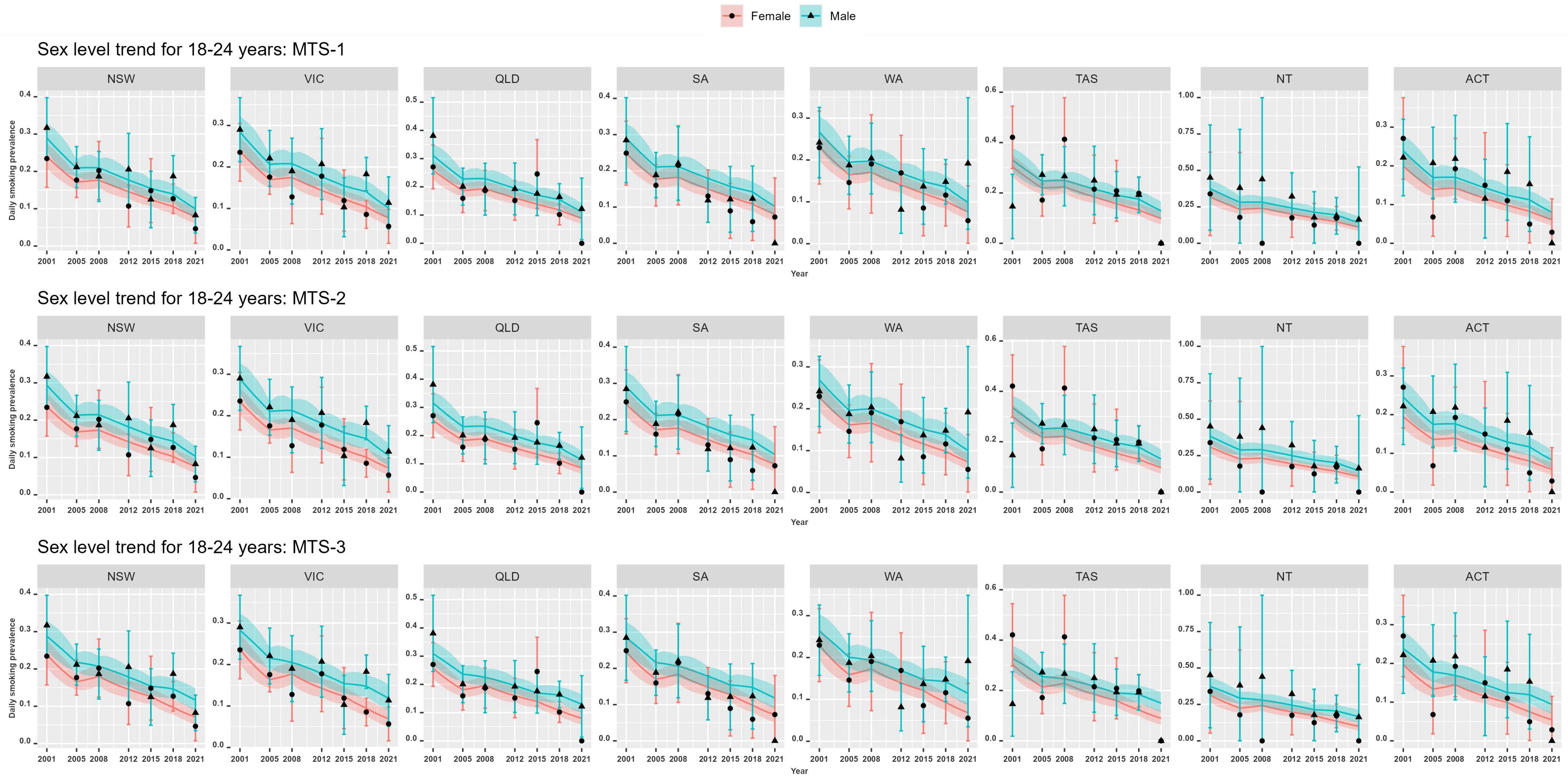

Figure S.2.8: Sex-by-state level trends in daily smoking among adults aged 18-24 years in Australia during 2001-2021 estimated by DIR estimator (black error-bar line), and three model-based (MTS-1, MTS-2 and MTS-3) estimators with 95 per cent credible band.

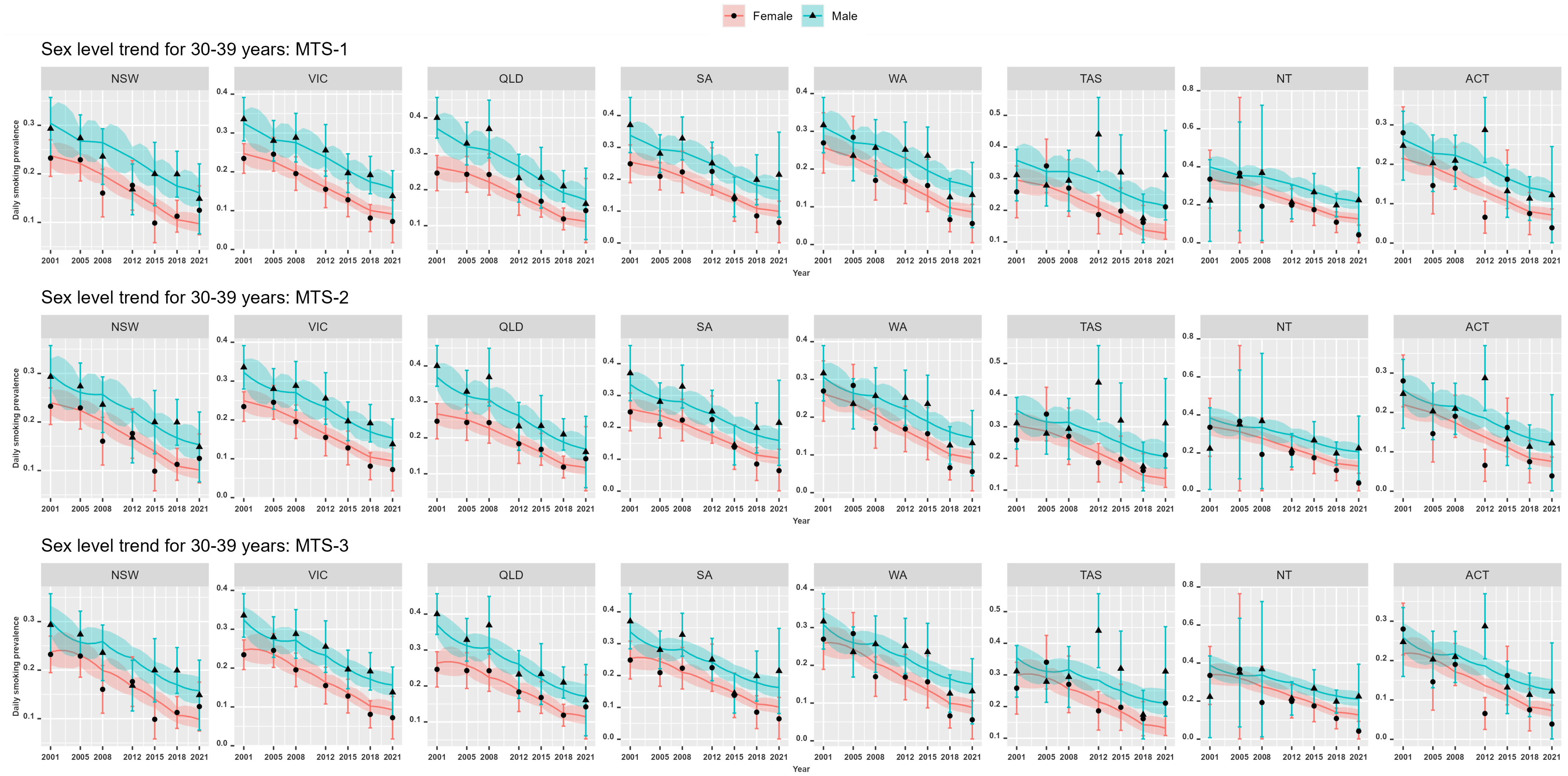

Figure S.2.9: Sex-by-state level trends in daily smoking among adults aged 30-39 years in Australia during 2001-2021 estimated by DIR estimator (black error-bar line), and three model-based (MTS-1, MTS-2 and MTS-3) estimators with 95 per cent credible band.

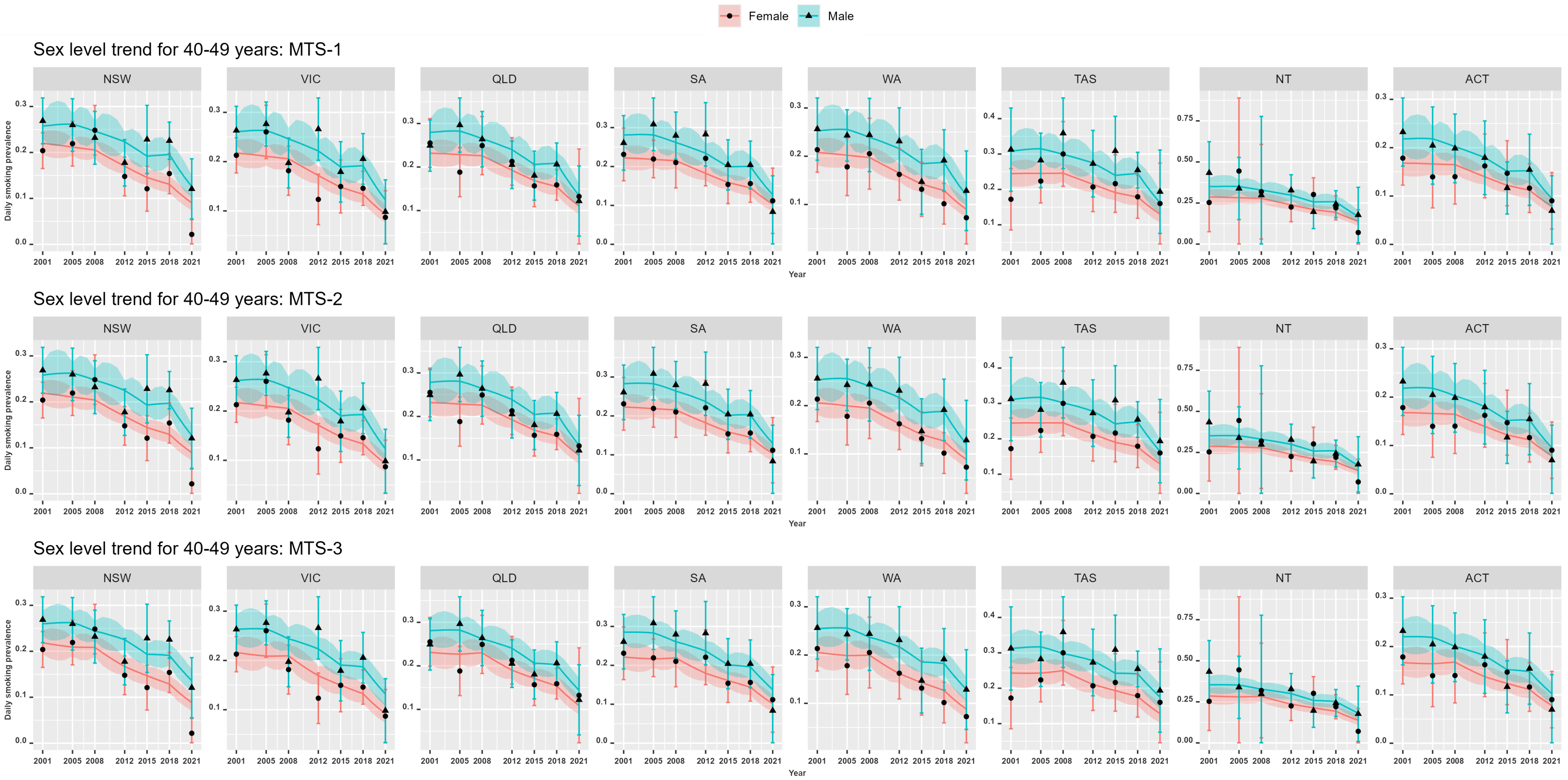

Figure S.2.10: Sex-by-state level trends in daily smoking among adults aged 40-49 years in Australia during 2001-2021 estimated by DIR estimator (black error-bar line), and three model-based (MTS-1, MTS-2 and MTS-3) estimators with 95 per cent credible band.

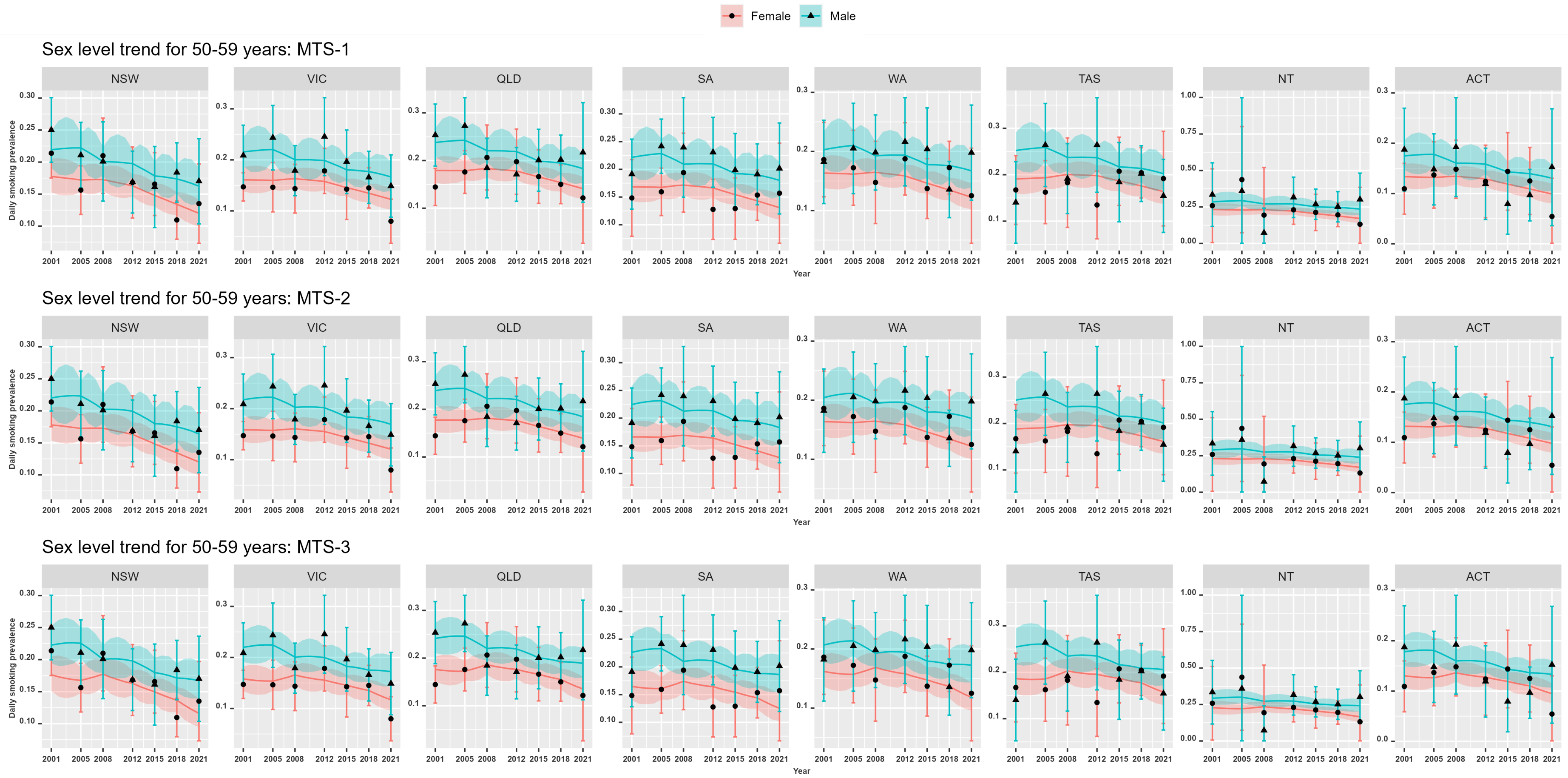

Figure S.2.11: Sex-by-state level trends in daily smoking among adults aged 50-59 years in Australia during 2001-2021 estimated by DIR estimator (black error-bar line), and three model-based (MTS-1, MTS-2 and MTS-3) estimators with 95 per cent credible band.

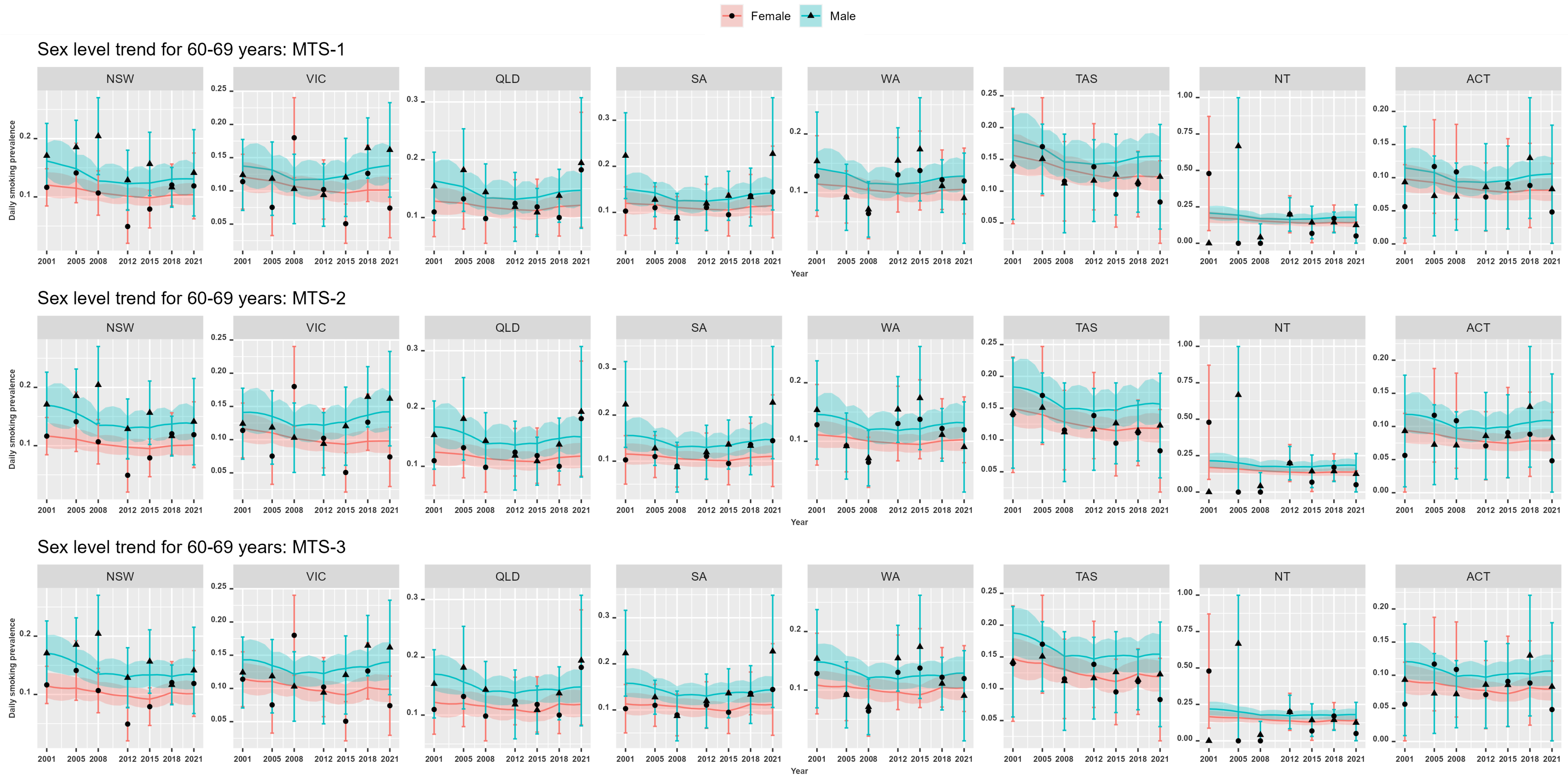

Figure S.2.12: Sex-by-state level trends in daily smoking among adults aged 60-69 years in Australia during 2001-2021 estimated by DIR estimator (black error-bar line), and three model-based (MTS-1, MTS-2 and MTS-3) estimators with 95 per cent credible band.

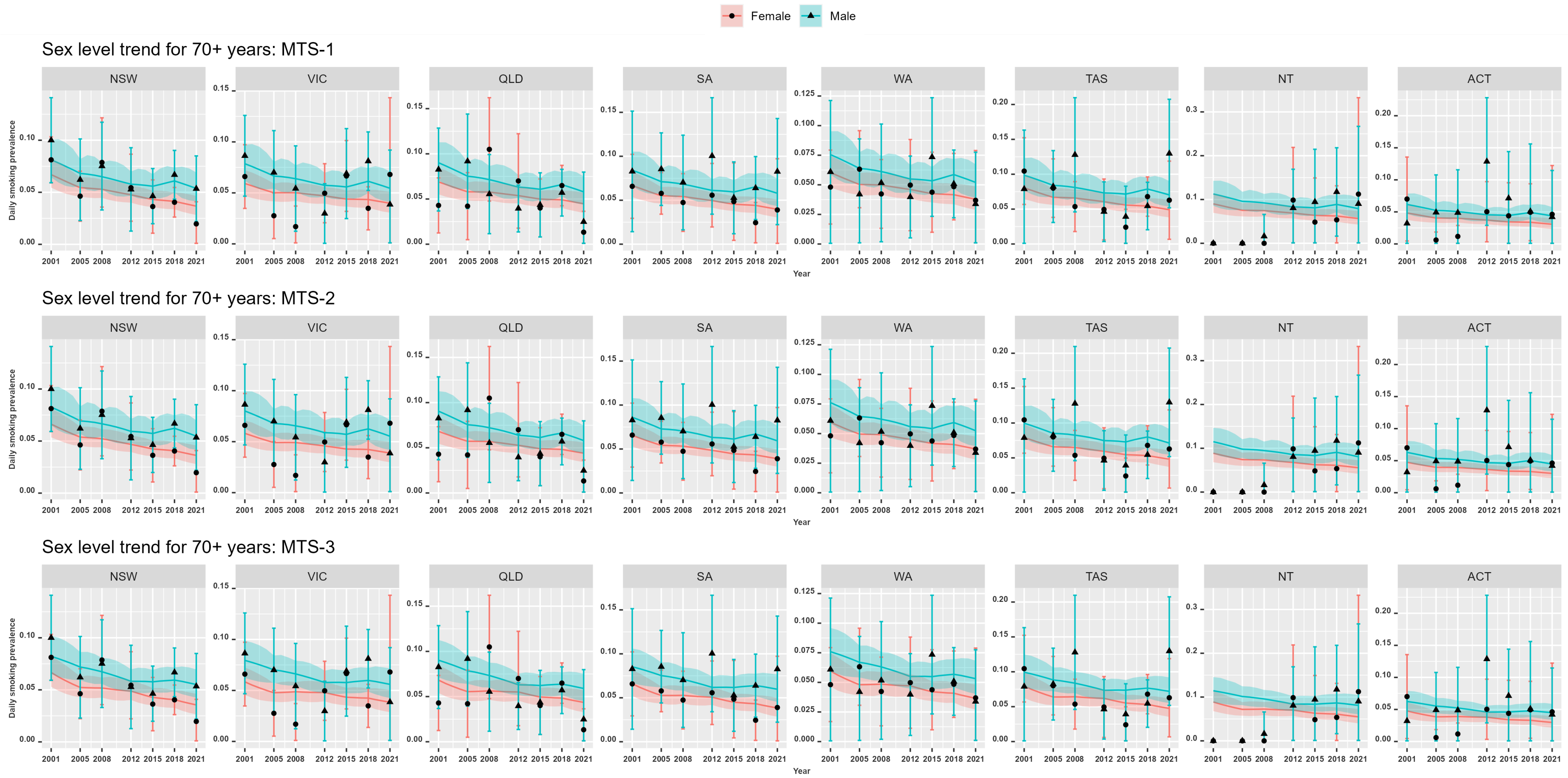

Figure S.2.13: Sex-by-state level trends in daily smoking among adults aged 70^{+} years in Australia during 2001-2021 estimated by DIR estimator (black error-bar line), and three model-based (MTS-1, MTS-2 and MTS-3) estimators with 95 per cent credible band.

S.2.3 Diagnostics of MCMC Draws

Diagnostics of MCMC draws are examined for the final selected model MTS-1 parameters. The convergence of the MCMC simulation for the model parameters is assessed through trace and autocorrelation plots as well as the Gelman–Rubin potential scale reduction factor, [? ?] known as $\hat{R}$, which diagnoses the mixing of the chains. The $\hat{R}$ statistic measures the ratio of the average variance of draws within each chain to the variance of the pooled draws across chains. If all chains are at equilibrium, these will be the same and $\hat{R}$ will be one, while if the chains have not converged to a common distribution, $\hat{R}$ will be greater than one [?]. The recommended maximum value of $\hat{R}$ is 1.1 [?]. The effective sample size (n-eff) is an estimate of the number of independent draws from the posterior distribution of the estimand of interest. If there is autocorrelation, effective sample size (n-eff) is usually smaller than the total number of sample size (i.e., total number of iteration). So the larger n-eff, as well as the larger the ratio of n-eff to total number of sample size, the better model performance [?]. Table S.2.4 shows the values of $\hat{R}$, n-eff and n-eff ratio to total number of sample size for the regression and variance parameters under the considered model. The trace plot of the corresponding 22 regression coefficients shown in Figures S.2.14, and S.2.15 also indicate better convergence of simulated values. The trace plot of standard deviation parameters shown in Figure S.2.16 also confirms the same.

We examined whether the autocorrelation coefficients are positive (which means the chain tends to stay in the same area between iterations) or they tend to drop quickly to zero with increasing lag. The autocorrelation function (ACF) plot for the regression coefficients shown in Figures S.2.17 and S.2.18 and for the standard deviation parameters shown in Figure S.2.19 confirm that ACF values go down to zero very quickly.

The posterior uncertainty intervals known as “credible intervals” are also plotted to see their distributions. The posterior uncertainty intervals for the regression coefficients and the standard deviation parameters are shown in Figure S.2.20 and S.2.21 respectively. Both Figures also confirm the significance of the parameters.

Name	R-hat	n_eff	n_eff_ratio
Intercept	1.00	6000.00	1.00
Sex: Male	1.00	5898.48	0.98
Age: 25-29	1.00	6000.00	1.00
Age: 30-39	1.00	6000.00	1.00
Age: 40-49	1.00	6000.00	1.00
Age: 50-59	1.00	6000.00	1.00
Age: 60-69	1.00	5762.49	0.96
Age: 70+	1.00	5106.47	0.85
State: NSW	1.00	5687.73	0.95
State: NT	1.00	5961.55	0.99
State: QLD	1.00	5677.40	0.95
State: SA	1.00	5720.93	0.95
State: TAS	1.00	5882.64	0.98
State:VIC	1.00	5781.01	0.96
State: WA	1.00	5893.08	0.98
year.s	1.00	5734.28	0.96
Sex: Male X Age: 25-29	1.00	6000.00	1.00
Sex: Male X Age: 30-39	1.00	6000.00	1.00
Sex: Male X Age: 40-49	1.00	6000.00	1.00
Sex: Male X Age: 50-59	1.00	5945.65	0.99
Sex: Male X Age: 60-69	1.00	5700.93	0.95
Sex: Male X Age: 70+	1.00	5012.85	0.84
$\hat{\sigma}^2_I$	1.00	2250.00	0.37
$\hat{\sigma}^2_S$	1.00	2596.01	0.43
$\hat{\sigma}^2_{RW1}$	1.00	2446.17	0.41

Table S.2.4: R-hat, n-eff, and n-eff ratio of the regression and variance parameters under model MTS-1.

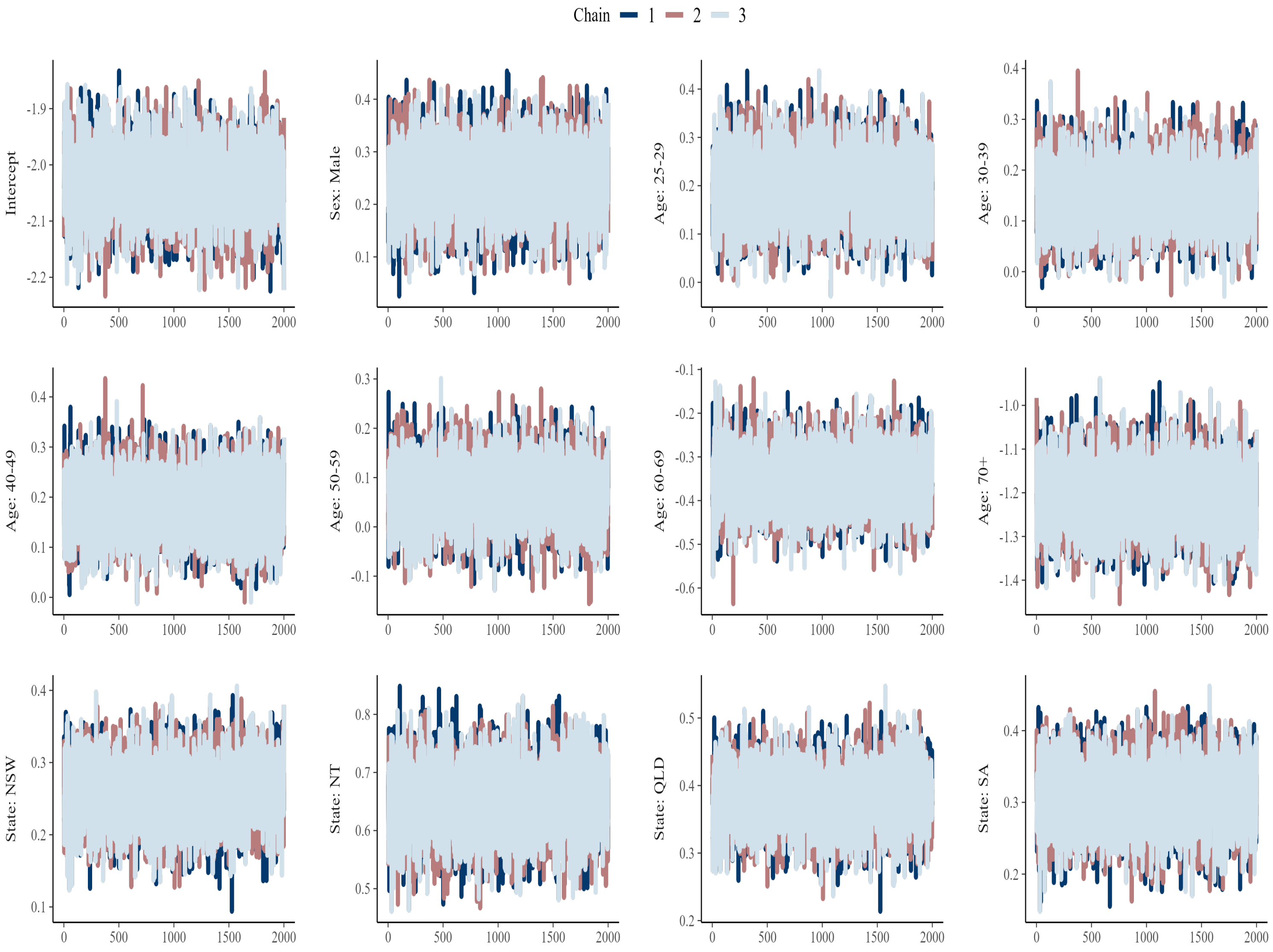

Figure S.2.14: Trace plot of the first 12 regression coefficients under model MTS-1.

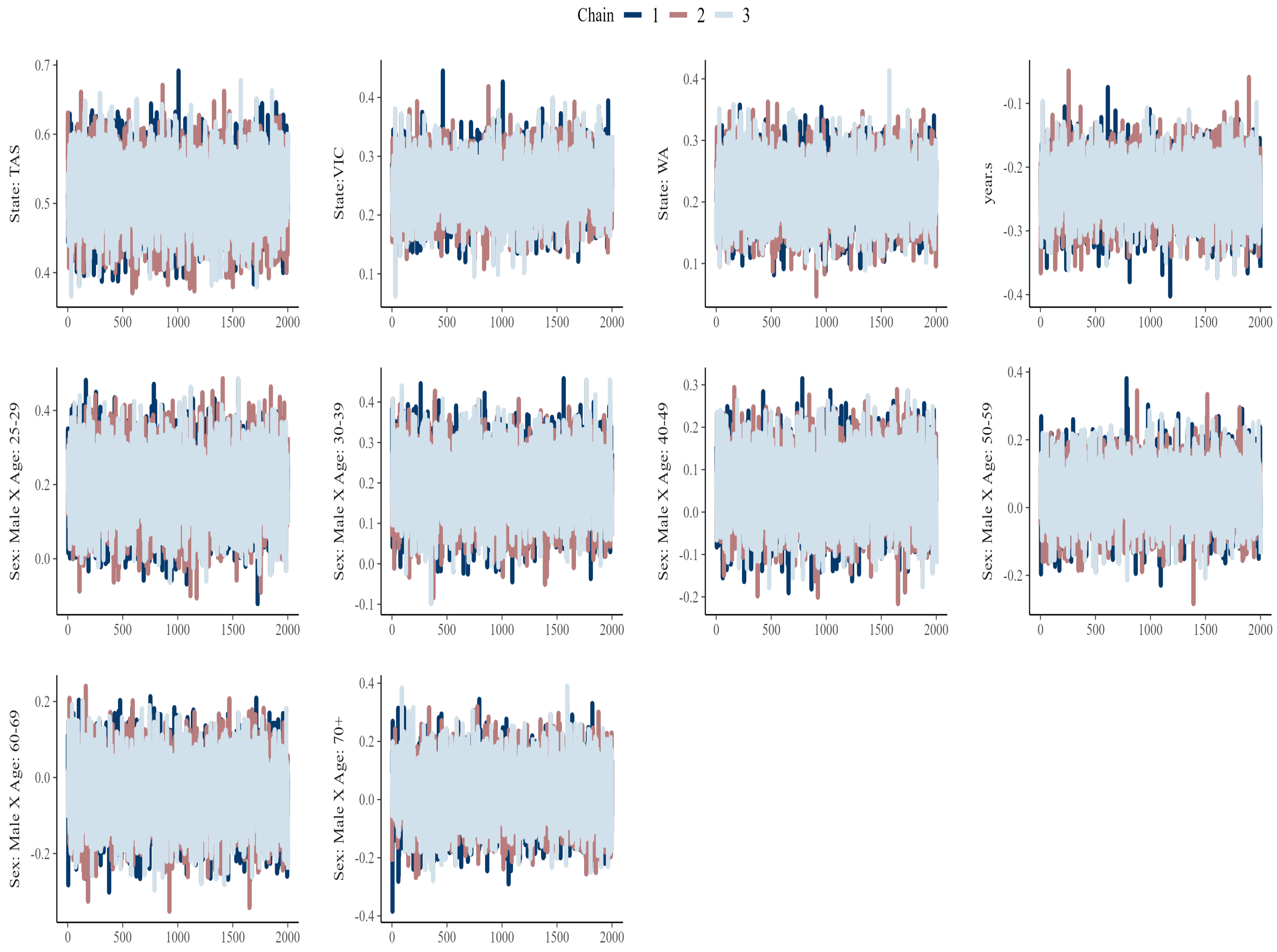

Figure S.2.15: Trace plot of the second 10 regression coefficients under model MTS-1.

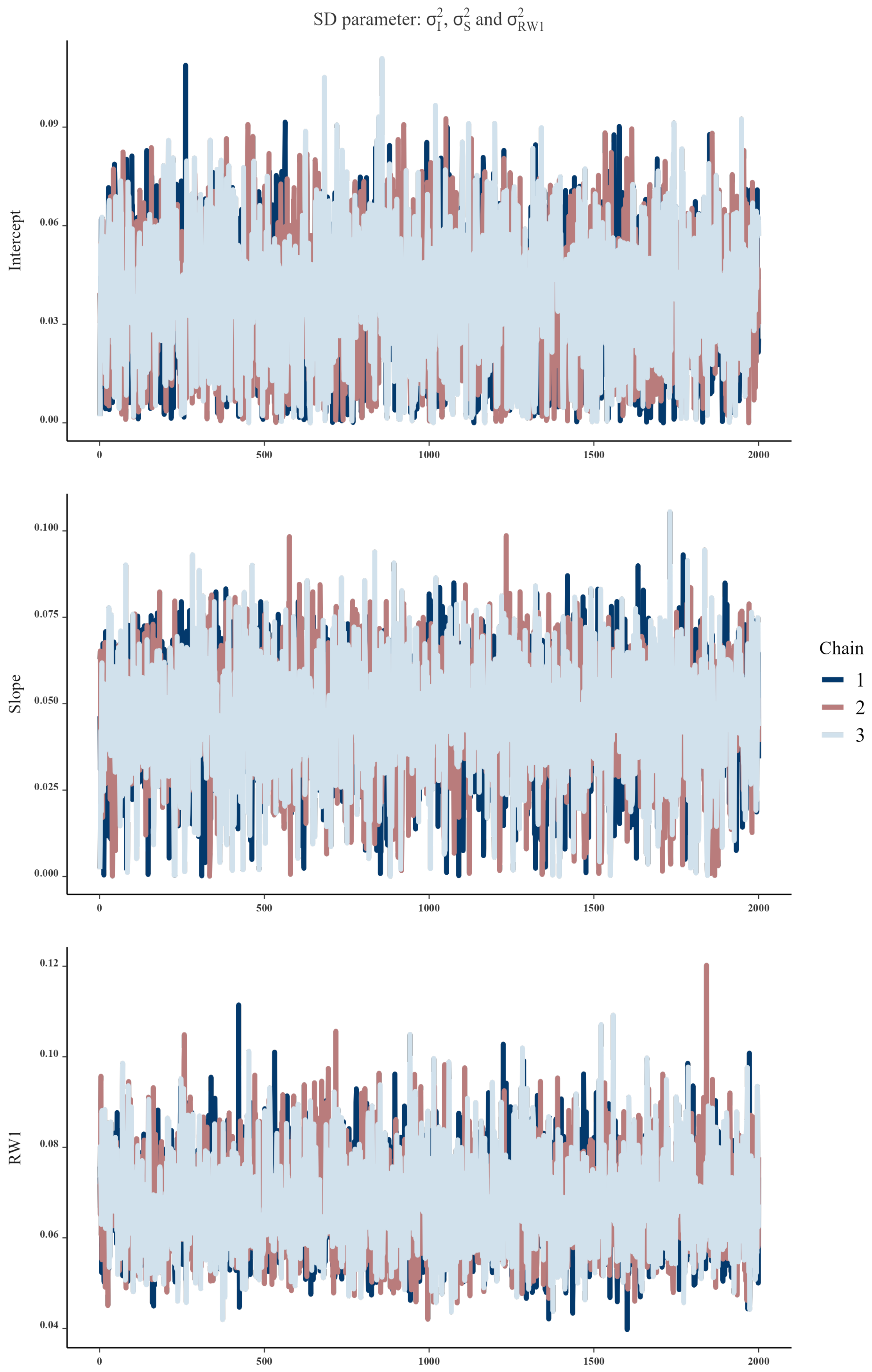

Figure S.2.16: Trace plot of the standard deviation (SD) parameters of *RIS* and *RW1* components in Table S.2.1 for model MTS-1.

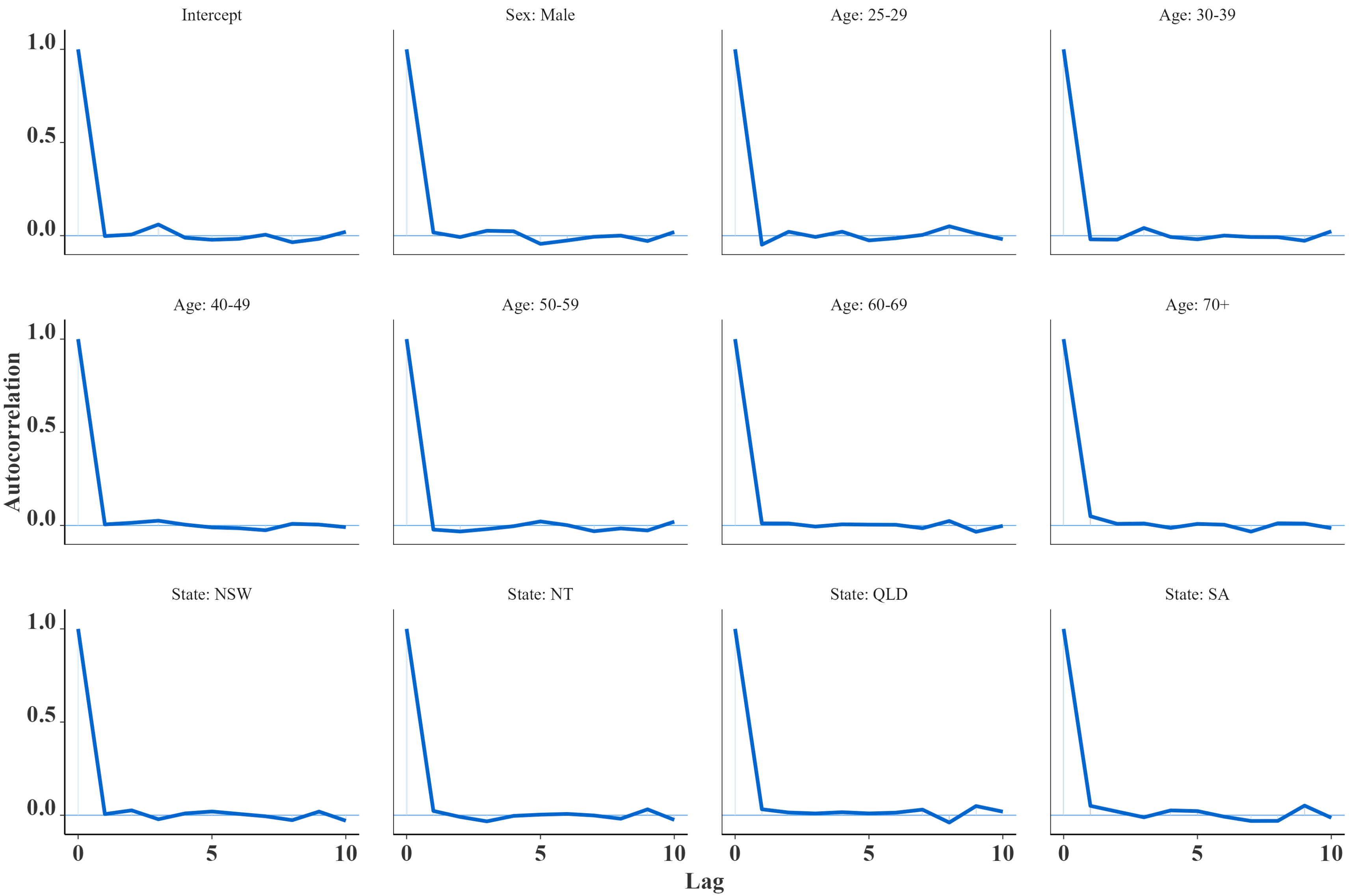

Figure S.2.17: Autocorrelation function (ACF) plot of the first 12 regression coefficients under model MTS-1 based on only one chain of 2000 draws.

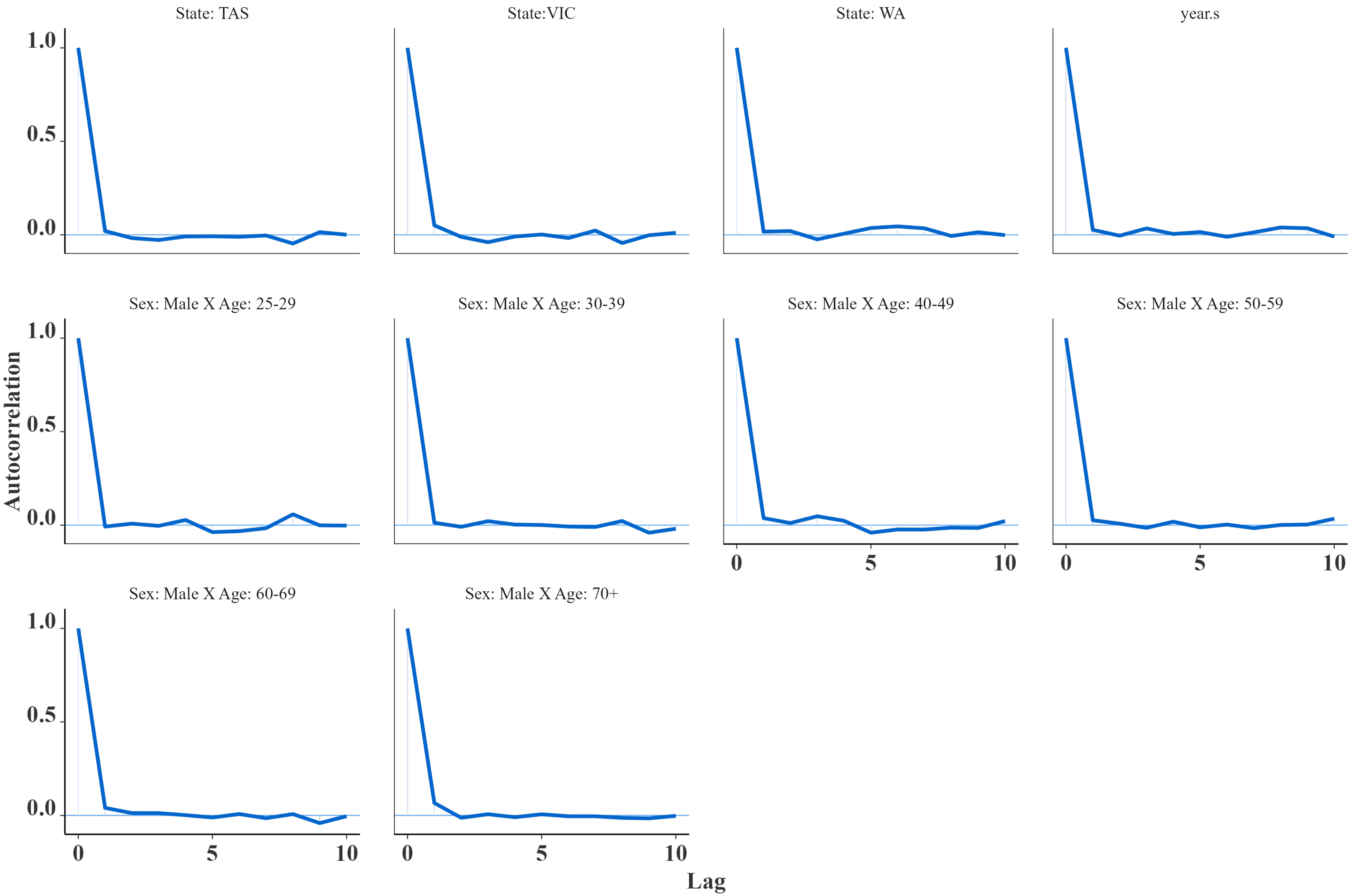

Figure S.2.18: Autocorrelation function (ACF) plot of the second 10 regression coefficients under model MTS-1 based on only one chain of 2000 draws.

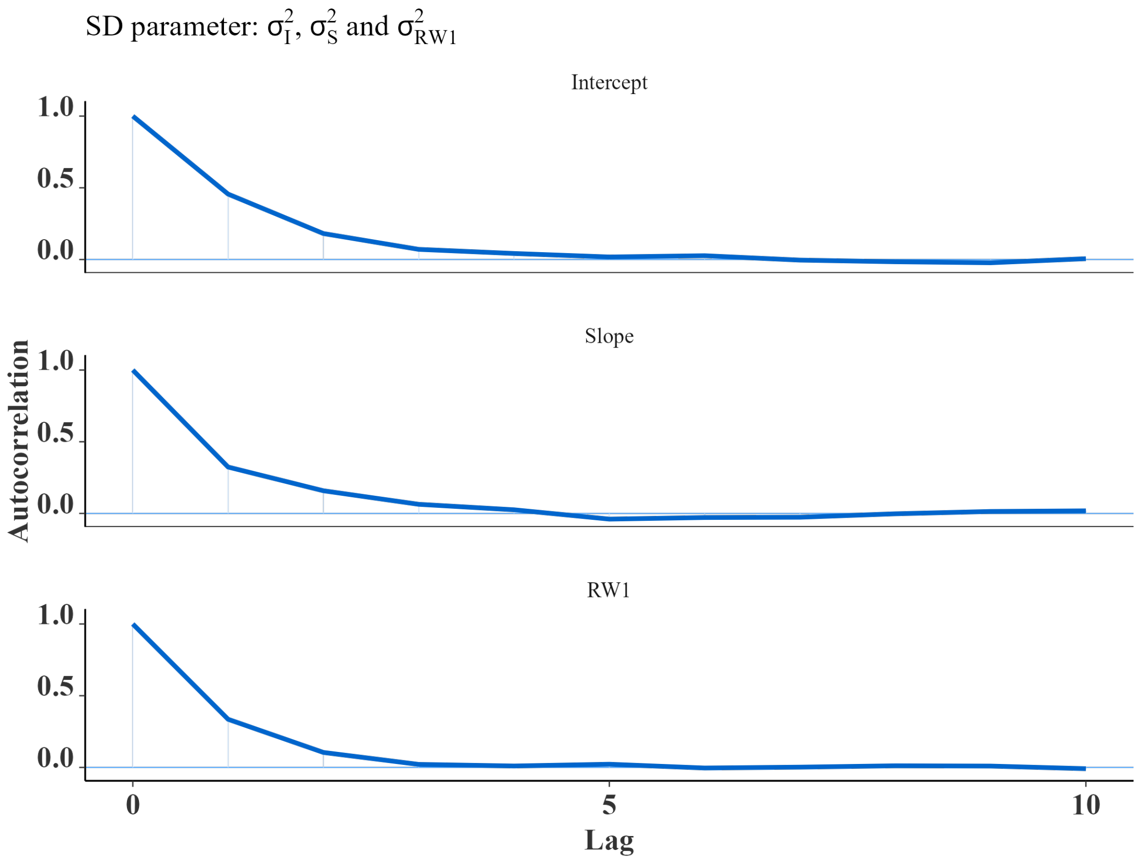

Figure S.2.19: Autocorrelation function (ACF) plot of the standard deviation parameters of *RIS*, and *RW1_1* components under Table S.2.1 for model MTS-1 based on only one chain of 2000 draws.

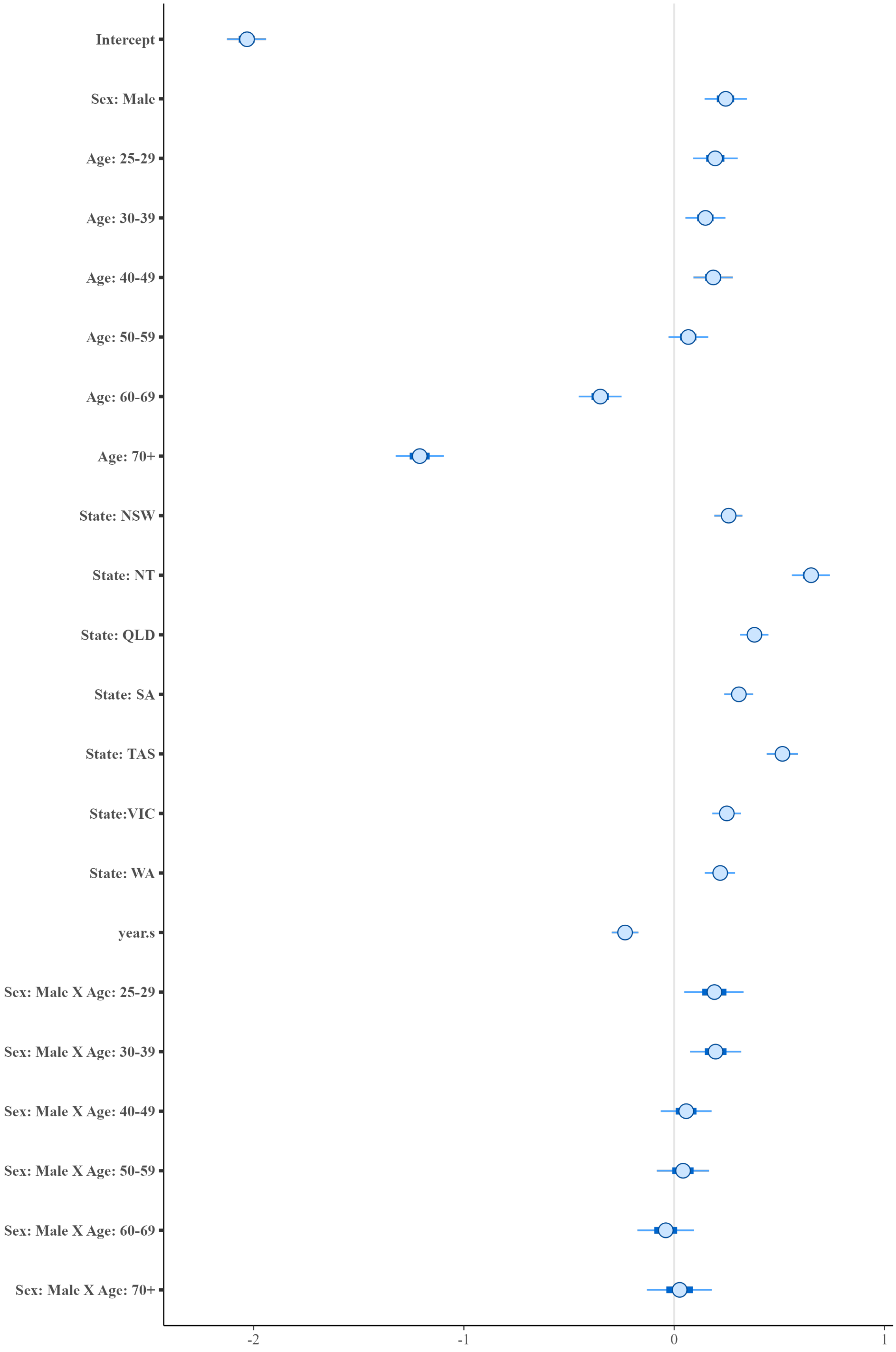

Figure S.2.20: Central posterior uncertainty interval of the regression coefficients under model MTS-1.

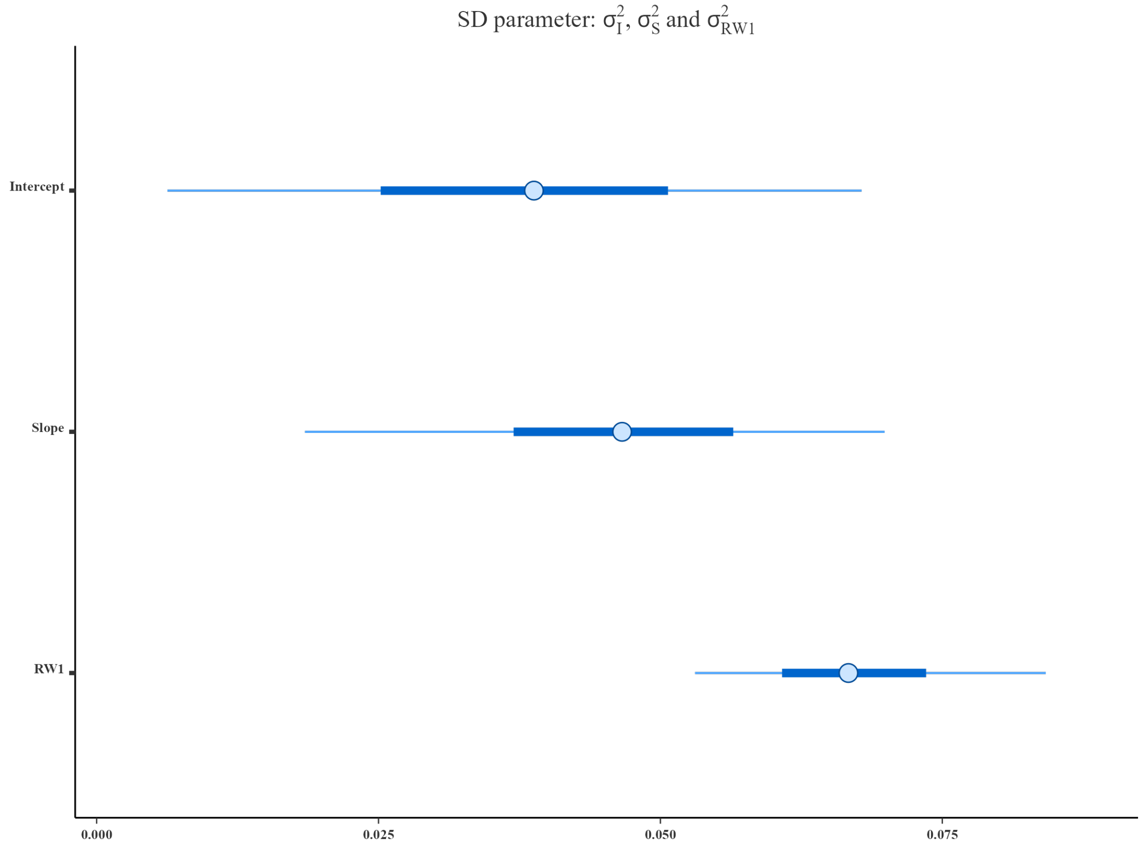

Figure S.2.21: Central posterior uncertainty interval of the standard deviation (SD) parameters for *RIS*, and *RW1_1* components in Table S.2.1 for model MTS-1.

S.2.4 Posterior Predictive Checks

As a final assessment for model validity we undertook posterior predictive checks. The idea behind posterior predictive checking is that a good model fit will enable us to generate data that resemble the observed data. For examining predictive performance of the fitted models, we compared observed data to simulated data from the posterior predictive distribution following [?] through utilization of the `R` package **bayesplot** [?]. It is recommended to compare graphical plots, such as the distribution of observed data and simulated data, looking for any systematic differences which could indicate potential failings of the model [?]. In this regard, Figure S.2.22 shows the kernel density estimate of the observed smoking prevalence (dark curves), with 2000 corresponding density estimates for simulated smoking prevalence (thin, lighter curves) drawn from the posterior predictive distribution (using the values from one chain only) at six levels: (i) national, (ii) Sex, (iii) age, (iv) state (and territory), (v) age-by-sex and (vi) the detailed level (state and territories cross-classified by age and sex groups) at which the model is fitted. The density plots suggest that the simulated values are following the density of observed smoking prevalence for different aggregated and diaggregated levels. The simulated density curves follow exactly at the higher aggregation level as expected. The most important characteristic is that the multimodal densities for the domains cross-classified by age groups (district-by-age, division-by-age and age-level in the Figure) are also mostly captured by the fitted model.

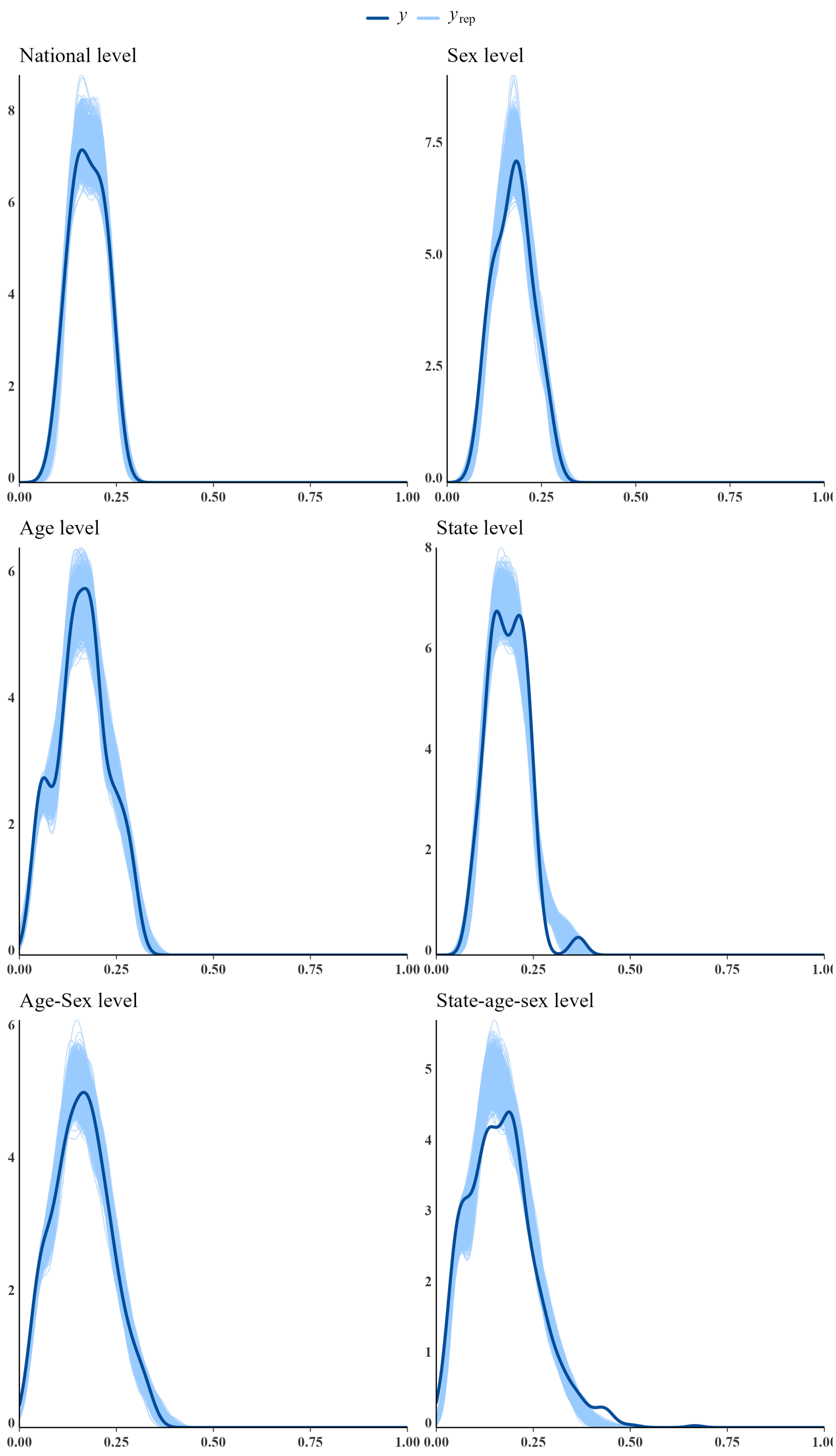

Figure S.2.22: Kernel density estimate of the observed data set (y, dark curves), with density estimates for 2000 simulated data sets y^{rep} drawn from the posterior predictive distribution (thin, lighter curves). The density plots are for the smoking level at (i) national , (ii) sex, (iii) age, (iv) state (and territory), (v) age-sex and and (vi) State-age-sex levels.

S.3 Trends of smoking prevalence at disaggregation levels

This section provides detailed model-based trend estimates for some disaggregated domains particularly be sex. Figure S.3.23 shows trends for the considered 7 age-groups by male and female. Figure S.3.24 show state and territories level trends of smoking prevalence by males and females. Then age-level trends by sex are shown for each of the state and territories are shown in Figures S.3.25-S.3.32 respectively for NSW, VIC, QLD, SA, WA, TAS, NT and ACT.

Figure S.3.23 shows that sex-wise difference in the trends is observed almost parallel over the whole time period for all the age-groups, except only for the 25-29 and 40-49 years age-group during 2018-2021. The magnitude in the sex-wise difference decreases with the time. An explicit pattern is observed for the younger 18-24 and 25-29 years adults. These results related to magnitude in sex-wise difference are consistent with the [9] results. On the other hand, the differences in the trends by sex seem significantly different for all the states and territories, however the sex-difference in smoking prevalence seem lowest in the NT compared to other states and territories (Figure S.3.24). In Figure S.3.31, the sex-differences seem non-significant for all the age-groups within the NT. Since the number of observed adults in NT are always smaller compared to other states, the gain of the model-based estimator is always found highest in the sex-age groups of NT.

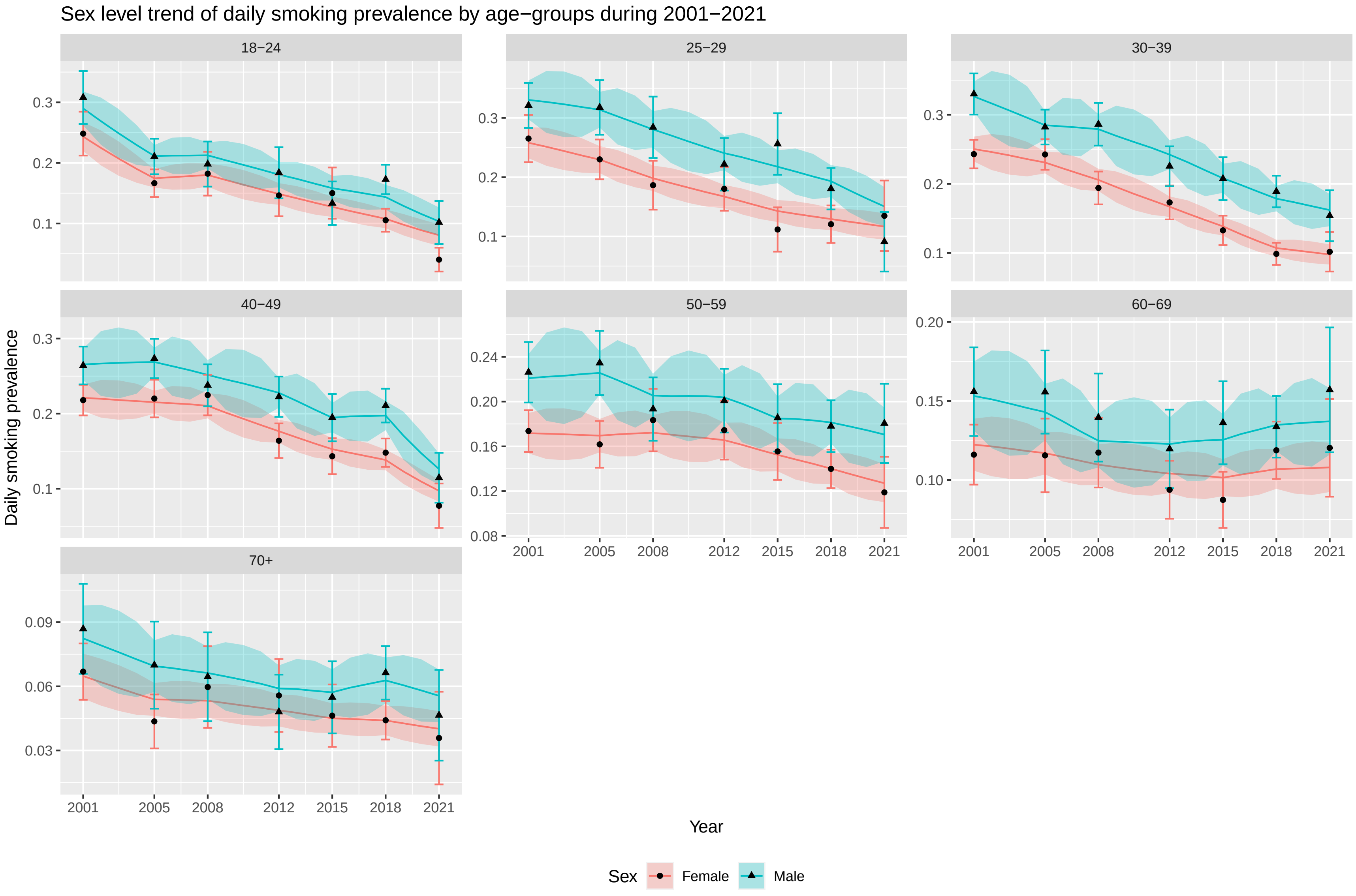

Figure S.3.23: Sex level trends of smoking prevalence in Australia by age-groups during 2001-2021 estimated by the direct (error-bar lines) and model-based (solid lines) estimators with 95% confidence band.

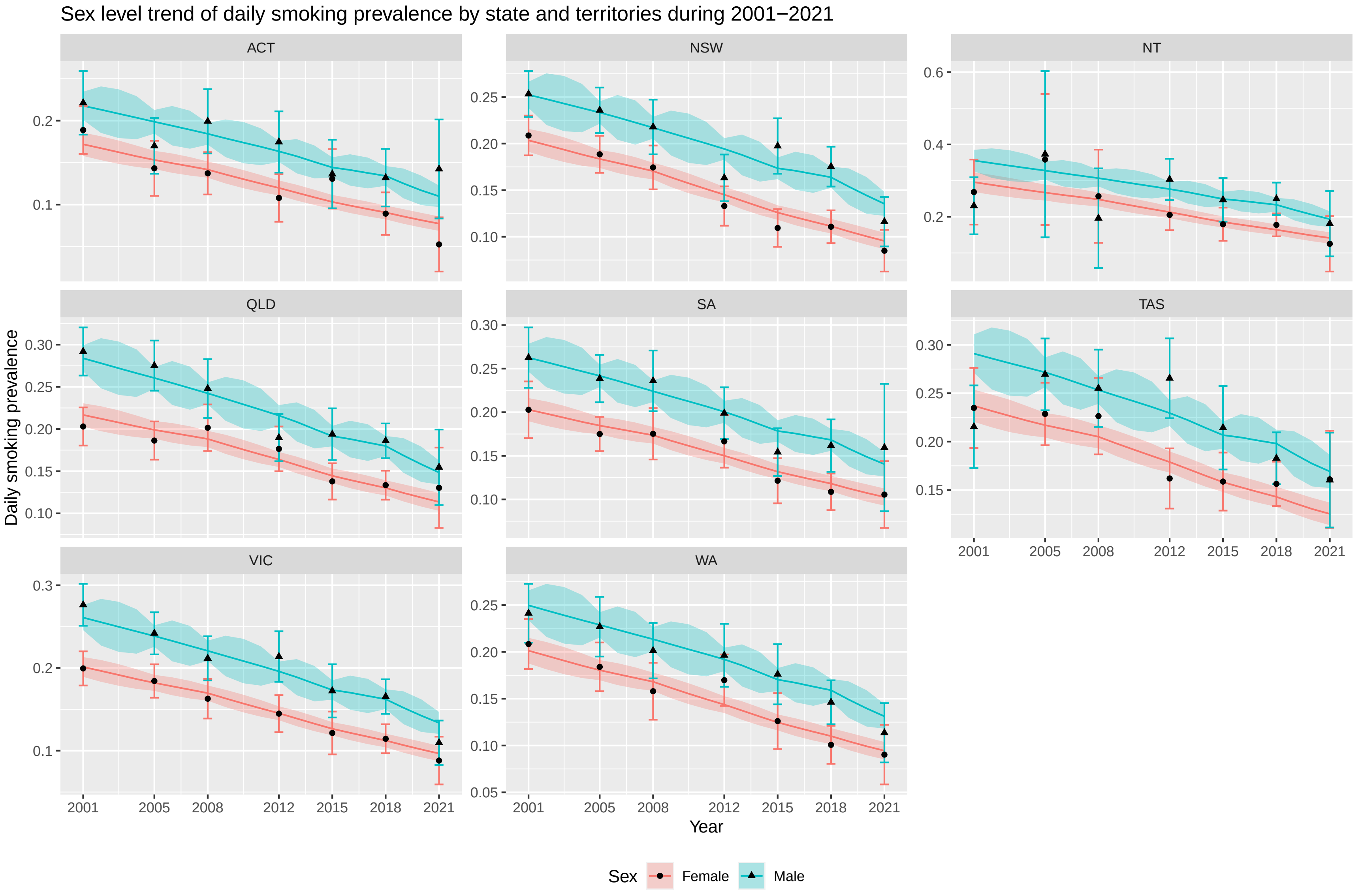

Figure S.3.24: Sex level trends of smoking prevalence in Australia by state and territories during 2001-2021 estimated by by the direct (error-bar lines) and model-based (solid lines) estimators with 95% confidence band.

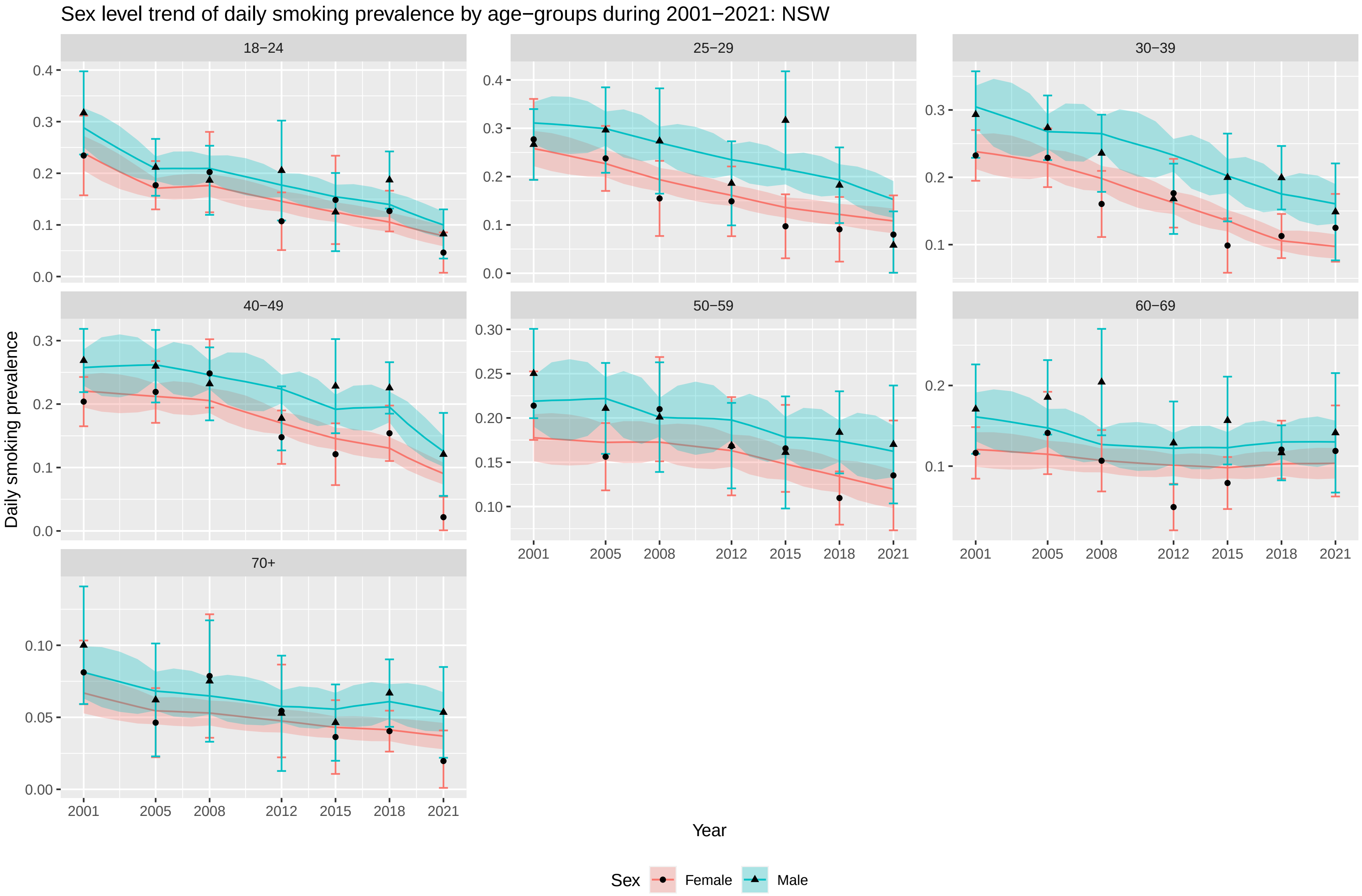

Figure S.3.25: Sex level trends of smoking prevalence in Australia by age-groups in **NSW** during 2001-2021 estimated by the direct (error-bar lines) and model-based (solid lines) estimators with 95% confidence band.

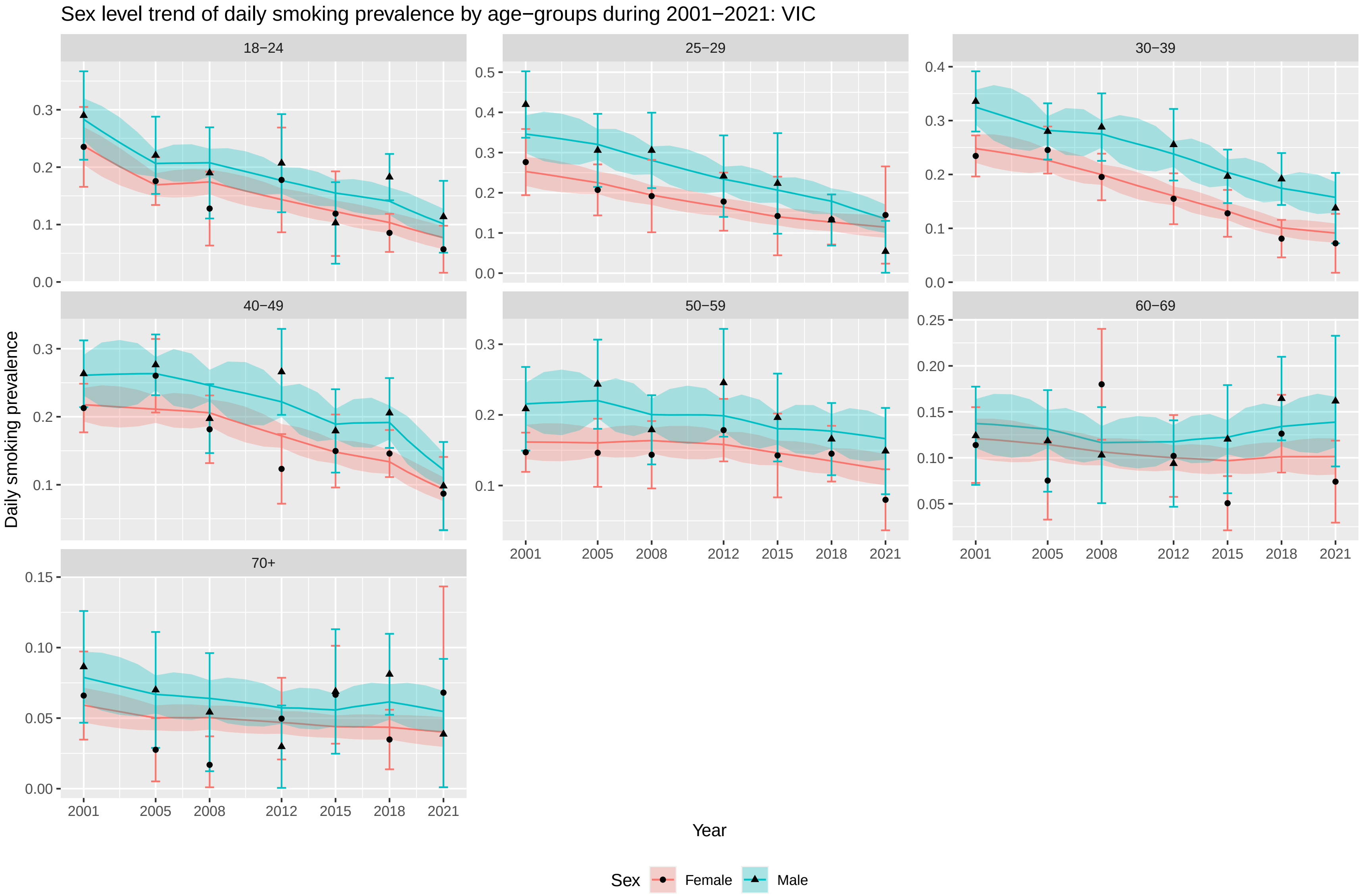

Figure S.3.26: Sex level trends of smoking prevalence in Australia by age-groups in **VIC** during 2001-2021 estimated by the direct (error-bar lines) and model-based (solid lines) estimators with 95% confidence band.

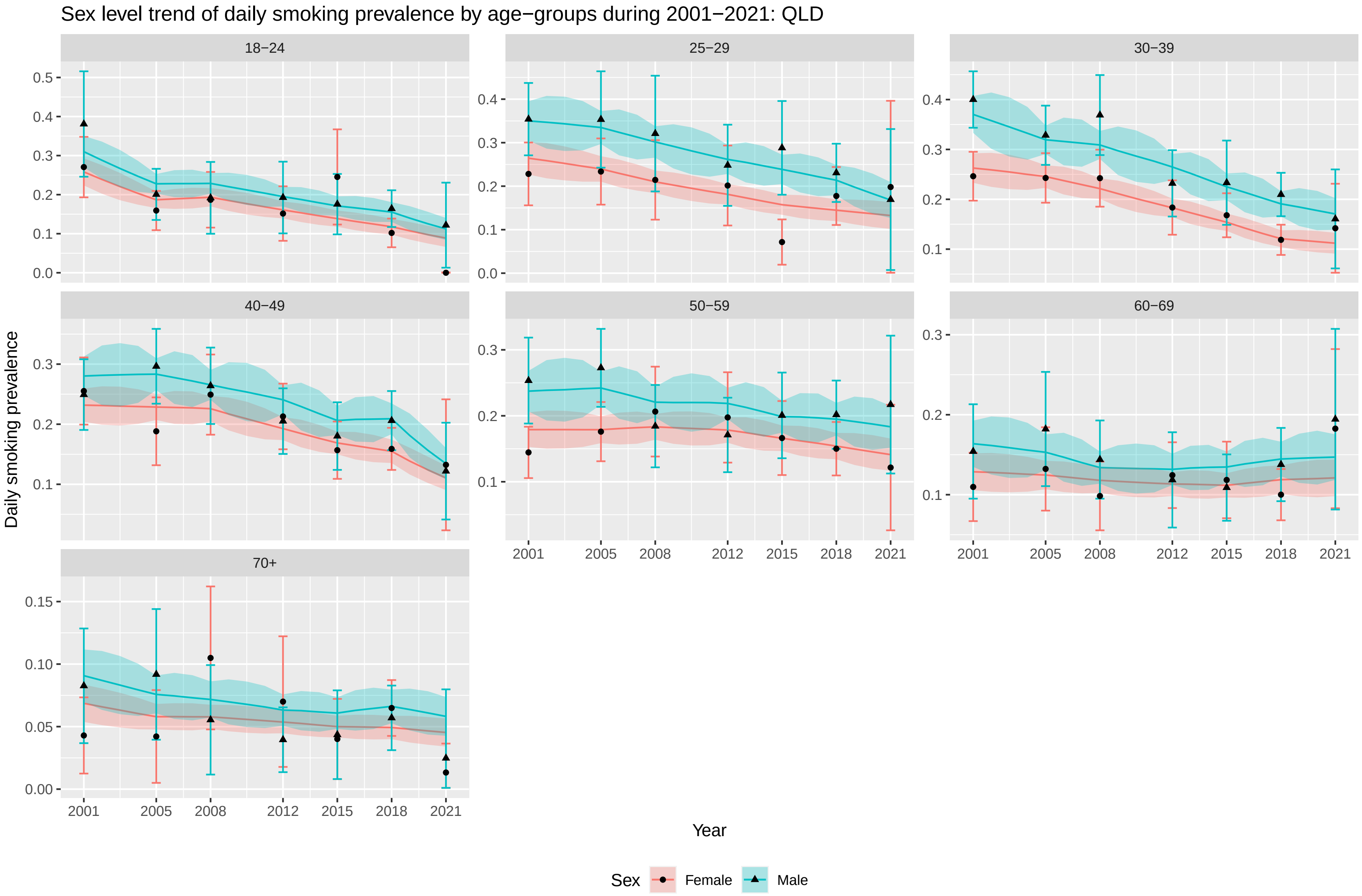

Figure S.3.27: Sex level trends of smoking prevalence in Australia by age-groups in **QLD** during 2001-2021 estimated by the direct (error-bar lines) and model-based (solid lines) estimators with 95% confidence band.

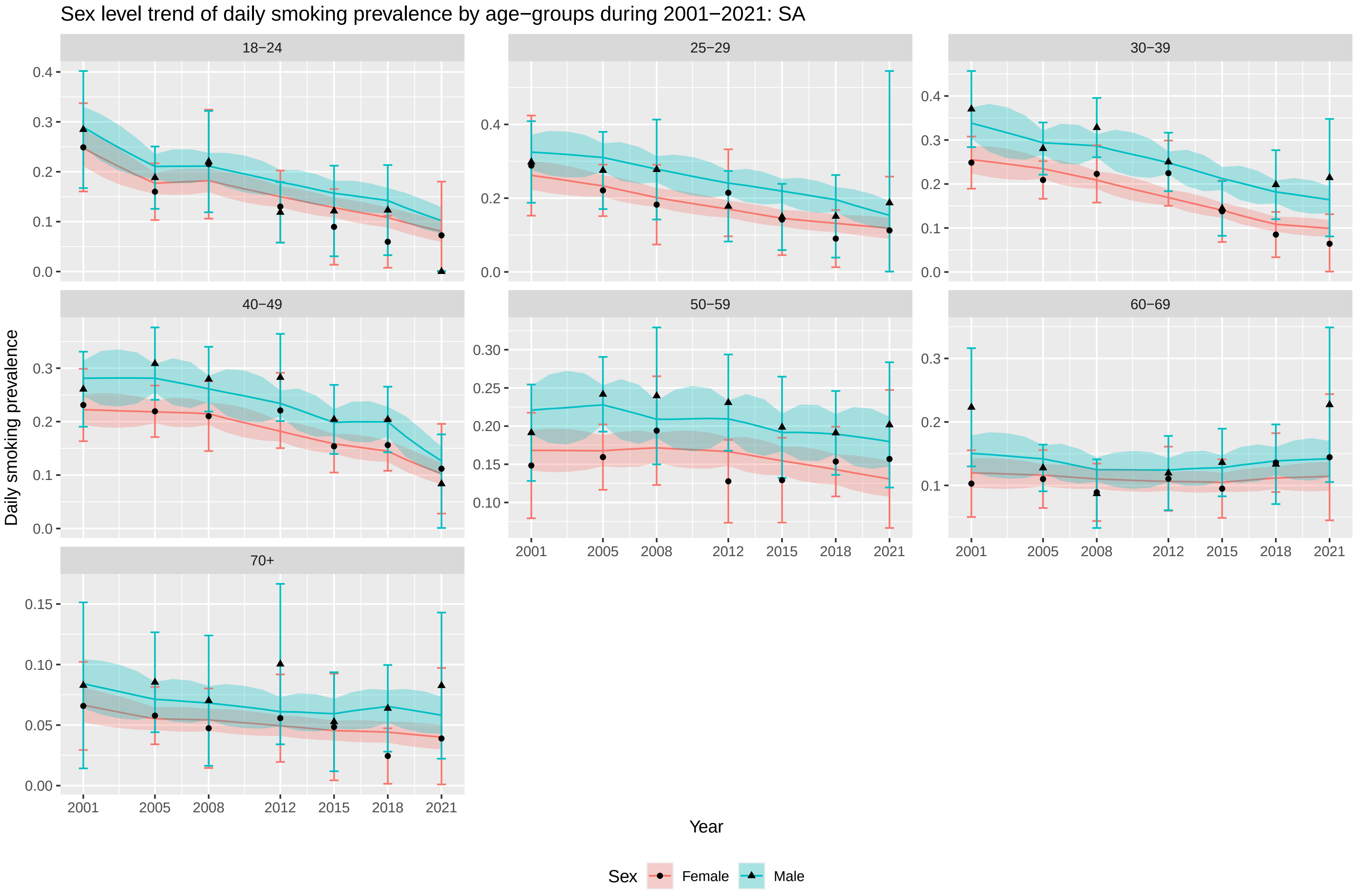

Figure S.3.28: Sex level trends of smoking prevalence in Australia by age-groups in **SA** during 2001-2021 estimated by the direct (error-bar lines) and model-based (solid lines) estimators with 95% confidence band.

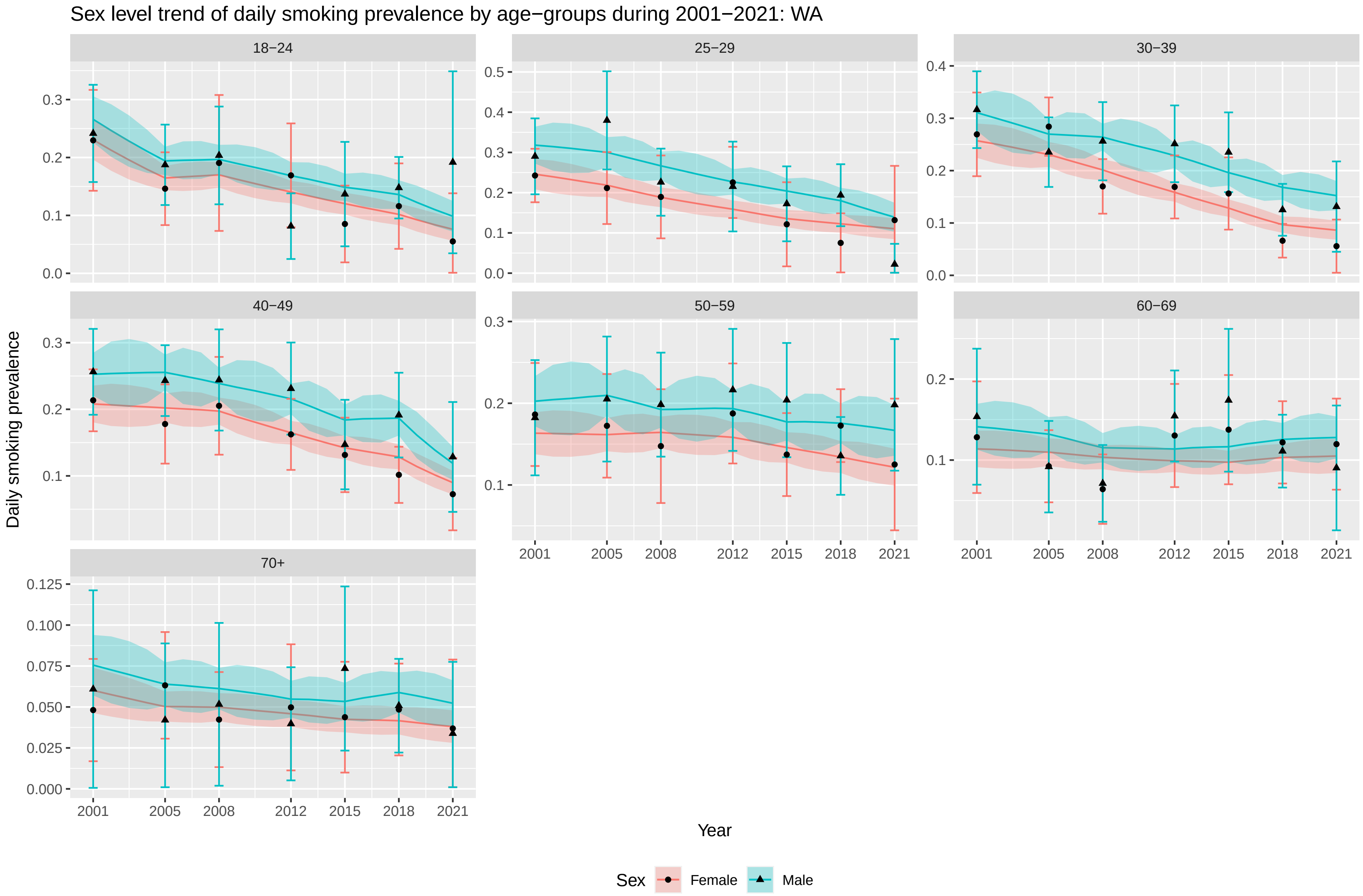

Figure S.3.29: Sex level trends of smoking prevalence in Australia by age-groups in **WA** during 2001-2021 estimated by the direct (error-bar lines) and model-based (solid lines) estimators with 95% confidence band.

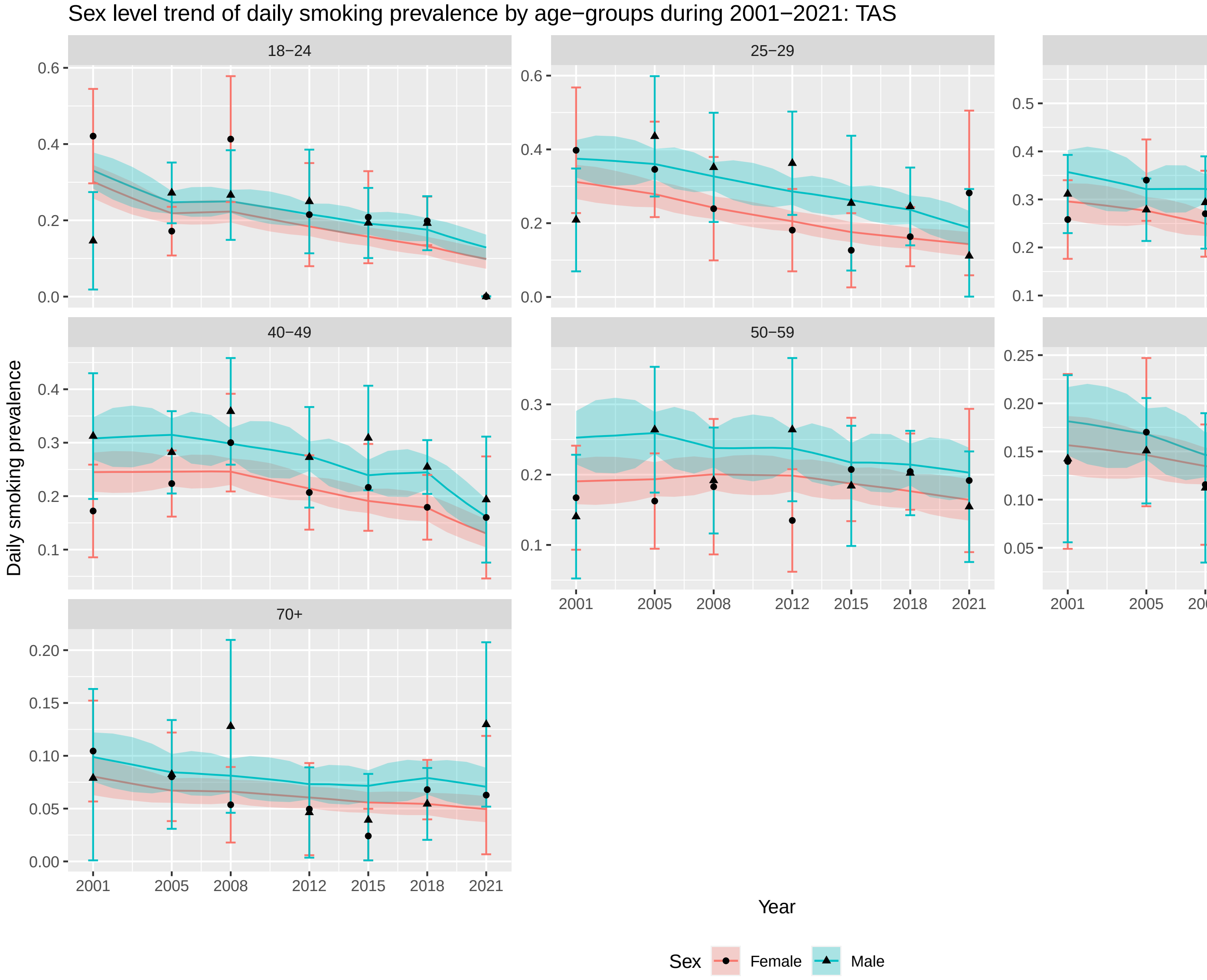

Figure S.3.30: Sex level trends of smoking prevalence in Australia by age-groups in **TAS** during 2001-2021 estimated by the direct (error-bar lines) and model-based (solid lines) estimators with 95% confidence band.

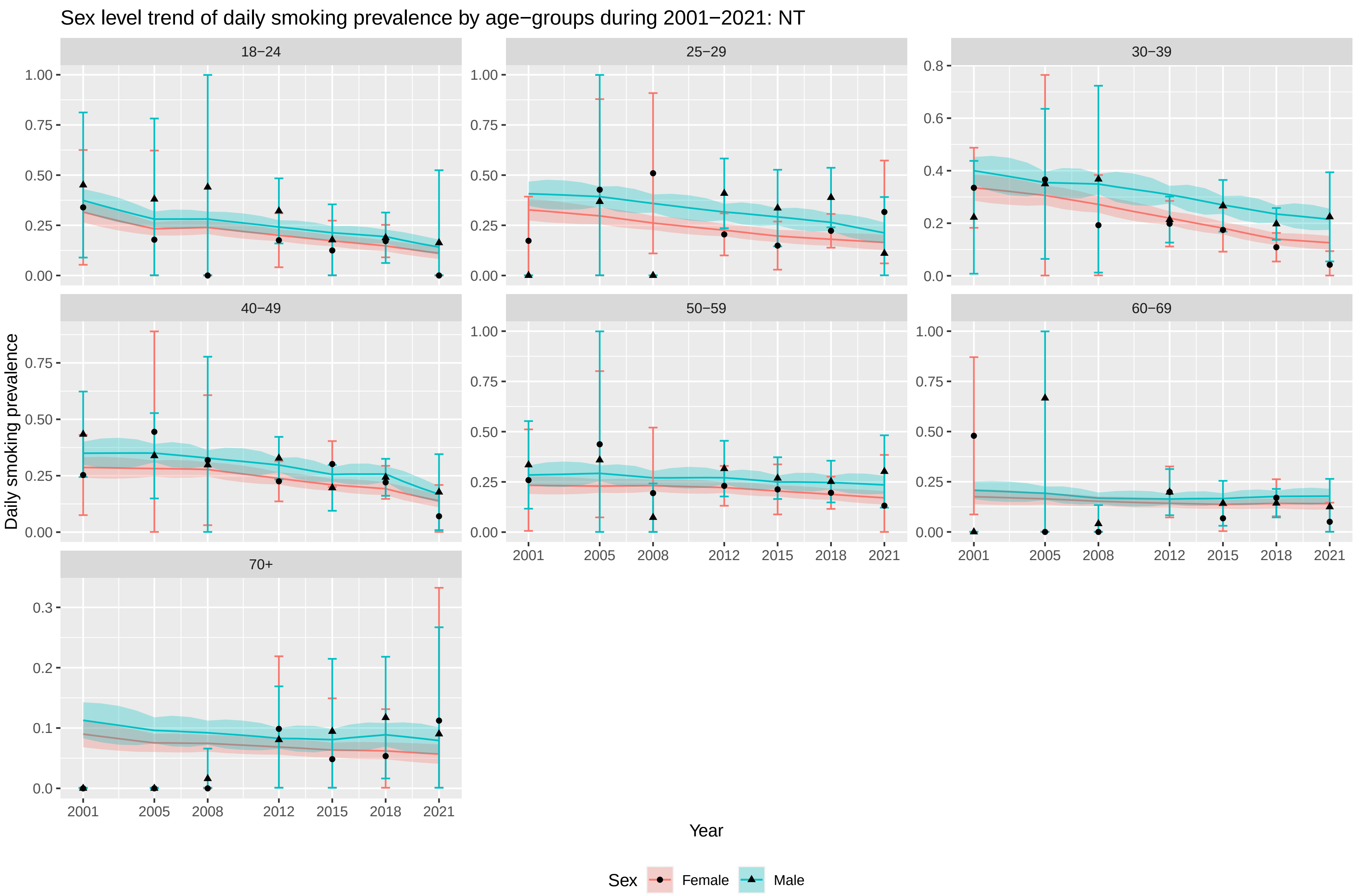

Figure S.3.31: Sex level trends of smoking prevalence in Australia by age-groups in **NT** during 2001-2021 estimated by the direct (error-bar lines) and model-based (solid lines) estimators with 95% confidence band.

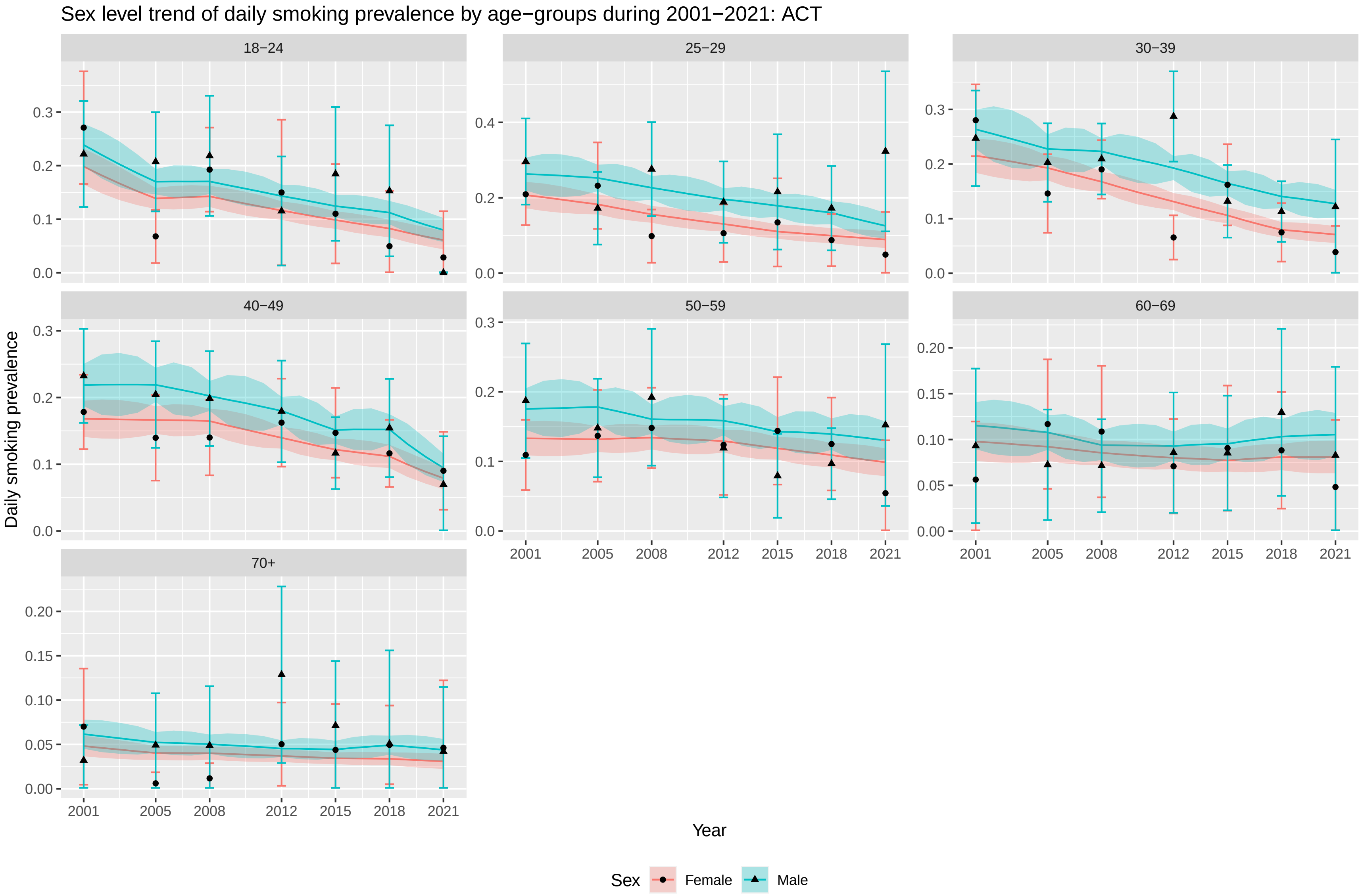

Figure S.3.32: Sex level trends of smoking prevalence in Australia by age-groups in **ACT** during 2001-2021 estimated by the direct (error-bar lines) and model-based (solid lines) estimators with 95% confidence band.

S.4 Joinpoint Trend Analysis

Joinpoint trend analysis has been conducted to explore the changes in smoking level trends through developing joinpoint regression models (which can be thought of as a piecewise regression) at different aggregation levels using the *Joinpoint* regression program, version 4.9.1.0 (Statistical Research and Applications Branch, National Cancer Institute). In the results provided in Figures S.4.33 to S.4.43 we have presented the direct graphical outputs provided by the *Joinpoint* program. However, in the results shown in the main paper, we have edited the figures to super-impose the different segments and calculated percent change for ease of visualisation.

As inputs for the joinpoint trend analysis over the study period 2001-2021, the model-based estimates of annual smoking prevalence along with their standard errors have been utilized. The trend estimates of smoking prevalence at the national, age, division, district and the most detailed levels have been included. The change in a particular segment of the trend is characterized by annual percent change (APC) by assuming (i) the outcome measure (for example, smoking level) changes at a constant percentage of the outcome value of the previous year and (ii) the outcome measure changes every year linearly on the log scale. A weighted average of the APCs, known as average annual percent change (AAPC), from the joinpoint model allows the use of a single number for describing the average annual change over a fixed period of multiple years. The AAPC values are comparable between cohorts even if the joinpoint model indicates that there were changes in trends during those years. The length of the APC interval is used as weight for the corresponding APC.

For selecting the best joinpoint model with appropriate number of segments (and hence number of change-points known as joinpoints), the widely used permutation test procedure illustrated in [**?**] has been used. It is noted that the joinpoint models in this study are developed assuming errors are uncorrelated and heteroskedastic with error variances characterised by estimated model-based standard errors of the smoking prevalence. For estimating confidence interval of APC and AAPC, it is assumed in the *joinpoint* software that the true AAPC follows a normal distribution and the true APC follows a t distribution. The values of APC and AAPC will coincide if the best joinpoint model has zero joinpoints, i.e., only one segment for the whole trend.

The statistical methodology of the joinpoint regression model is available in [**?**] and the detailed calculation procedure in *joinpoint* software is available in [14].

The basic steps for fitting the joinpont regression models in the *joinpoint* software can be briefly illustrated as below:

- Step 1: Creation of input data file. As an example, **smoking** as the dependent variable, **Year** as the independent variable, **se** as the standard error of the dependent variable, and as optional cohort variable denoted as "by variable" (say, **Age**). Since the dependent variable in this study is proportional, the dependent variable type is considered as "Proportion". The interval type is selected as "annual", modelling errors are chosen as "uncorrelated" and transformation of the model is chosen as "log".

- Step 2: Choose the options for "Method and Parameter". The default options are used

in this study. As for example, grid search method to consider minimum number of observations from a joinpoint to either end of the trend and between two joinpoints; maximum number of joinpoints is considered as 4 since the number of time points is 21 in this study; permutation test with 5 per cent overall significance level and 4499 permutations is considered as the model selection method, and the parametric method is considered for estimating confidence interval of APC and AAPC. There are some advanced analysis tools are available, but no advanced tools are used in this study.

- Step 3: Explore the joinpoint models. The software shows models for all possible joinpoints along with the indication of best model.
- Step 4: Export APC and AAPC values. The values of APC and AAPCs along with their significance test, confidence interval, period of segment and the statistical evidence of model selection can be exported from the software. The graphs showing observed values and the corresponding fitted lines with APC values can be exported.

The joinpoint regression model with the best fit is considered for generating the graphs and extracting the values of APC and AAPC. The joinpoint regression model for national level trend shown in Figure S.4.33 indicates significant declining trends in smoking prevalence with four joinpoints (i.e., five segments). The highest annual decrease is found in 2018-2021 (5.6 per cent with 95 per cent CI: 3.9-5.1 per cent), while the longest segment in 2001-2008 with APC -2.25 (95 per cent CI: -3.3, -2.7) (See Figure S.4.33).

The joinpoint fitted models by sex are shown in Figure S.4.34. For both models, the number of joinpoints is observed four. The APCs show that the male has lower percentage change over the whole time period except the last segment 2018-2021 (APC: -6.11 for male and APC: -4.8 for female). Also, female adults show gradual and consistent declining trend over the whole time period with average annual percentage change of about -3.5.

The joinpoint models by age-groups are shown in Figure S.4.35. For most of the age-groups the number of joinpoints is observed four except for the younger age-groups 18-24 and 25-29. The joinpoints of 18-24 years age-group shows a non-significant increasing trend with APC=0.65 during 2005-2008. The joinpoint trend analysis by age level shown in Figure S.4.35 indicates how the progress in declining the smoking level is poorer among older age-groups 50-59, 60-69 and 70^{+} years compared to the young and middle aged groups. Notably, a significant decreasing trend (7.8 per cent per year) was observed during 2001-2005 for 18-24, followed by a slight increase of 0.6 per cent. The highest APC value (-12.7) is observed for the 40-49 years group during 2018-2021, though the APC value was very small at the very beginning time period 2001-2007. The overall pattern is that the annual percentage change is higher for the younger age-groups (18-24 and 25-29) and the lowest among the older age-groups (50-59, 60-69 and 70^{+}).

The joinpoint models by state and territories are shown in Figure S.4.36. For all the states, the number of joinpoints is observed four and the joinpoints are in the same years as 2008, 2012, 2015, and 2018. For all the state, the highest percentage change has been observed in the last time segment 2018-2021. All the states and territories show similar pattern of annual percentage change, however, NT shows lower rate of changes compared to the ACT. The level of smoking prevalence is also found considerably lower in ACT and higher in NT.

The joinpoint models for cross-classified domains of age-groups and sexes are shown in Figures S.4.37 to S.4.43. The same graphs are also shown in the main paper but in different form for easy presentation. For the young and middle age-groups, the male adults have higher rate of decline than the female adults. These pattern is more obvious for the 25-29 and 40-49 year age-groups. The direct estimates obtained from the 2021 NHS confirms that this pattern for the model-based estimates are reasonable. For the three older age-groups, annual percentage changes are very low and even for the 60-69 year some time segments have positive APC values (see Figure S.4.42).

The estimated values of average annual percentage change (AAPC) in the smoking prevalence at state-by-age obtained from the joinpoint regression analysis are shown in Table S.4.2. The AAPC values decrease with the age-groups and the pattern seem very similar. As expected, the AAPC values are found lowest for the NT among the younger age-groups. For the older age-groups, the change rates are lower and no considerable difference is observed by state and territories.

The values of AAPCs for the cross-classified domains of state and sex are shown in Table S.4.1. For most of the states and territories, the female adults have higher AAPC values, however for QLD the values are found same. The lowest improvement in the decline of smoking prevalence is observed in TAS, while highest in ACT for male and female adults.

The average annual percentage change in the trends of smoking level at the most detailed level domains constructed by 8 state and territories, 2 sexes and 7 age-groups are shown in Tables S.4.3 to S.4.10 by state and territories respectively. The lowest difference in the AAPC values are found among age-sex domains in QLD. The equal values of AAPC by sex in QLD are mainly due to lower difference in the AAPC values for the cross-classified domains of state and sex (see Table S.4.5. Also, the AAPC value for 25-29 years old male (AAPC: -3.6) is observed slightly higher than their female counterparts (AAPC: -3.4).

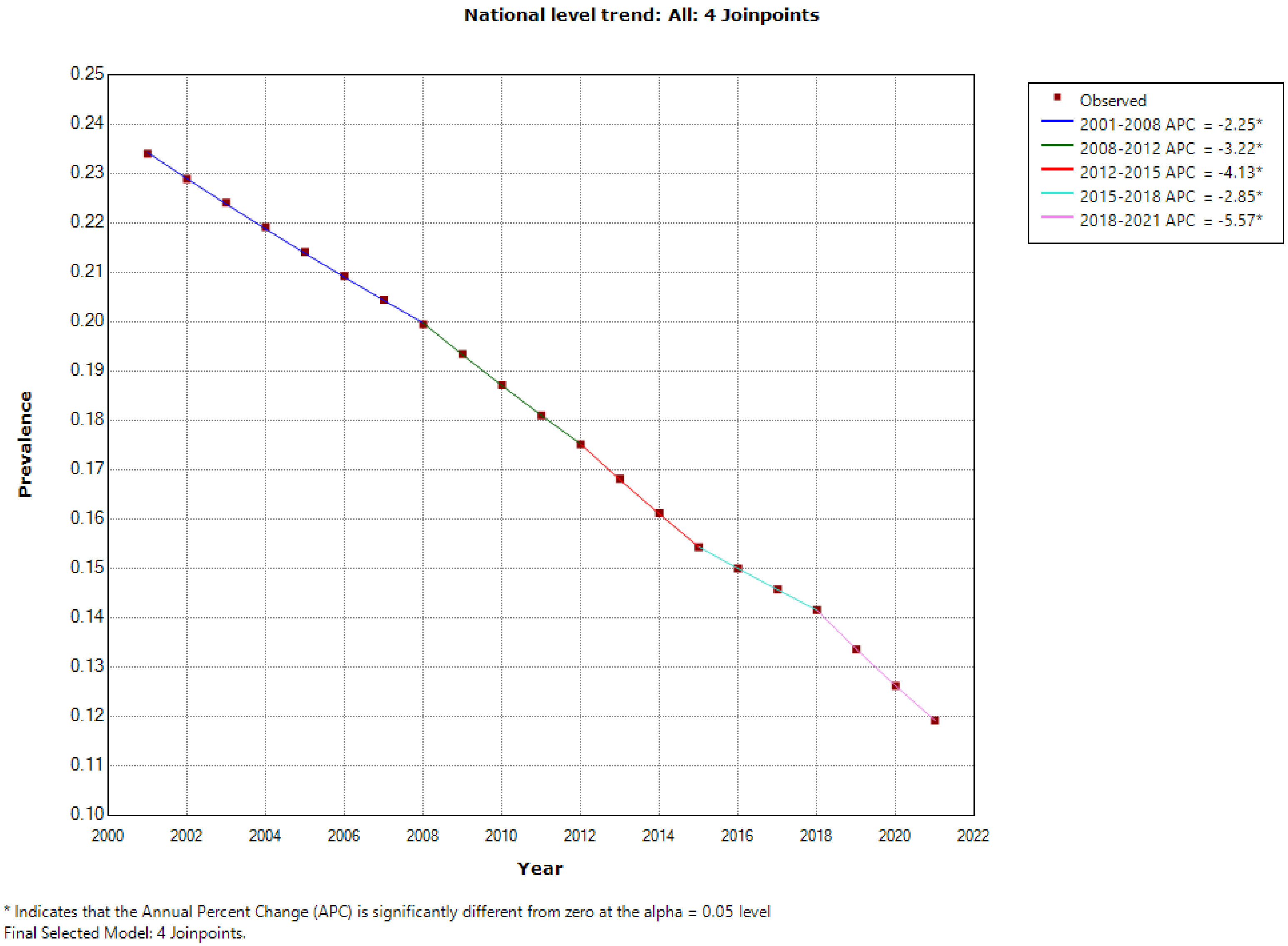

Figure S.4.33: Joinpoint regression models for the national level trend of smoking prevalence during 2001-2021. ⋆ indicates that the APC is significantly different from zero at 5 per cent level.

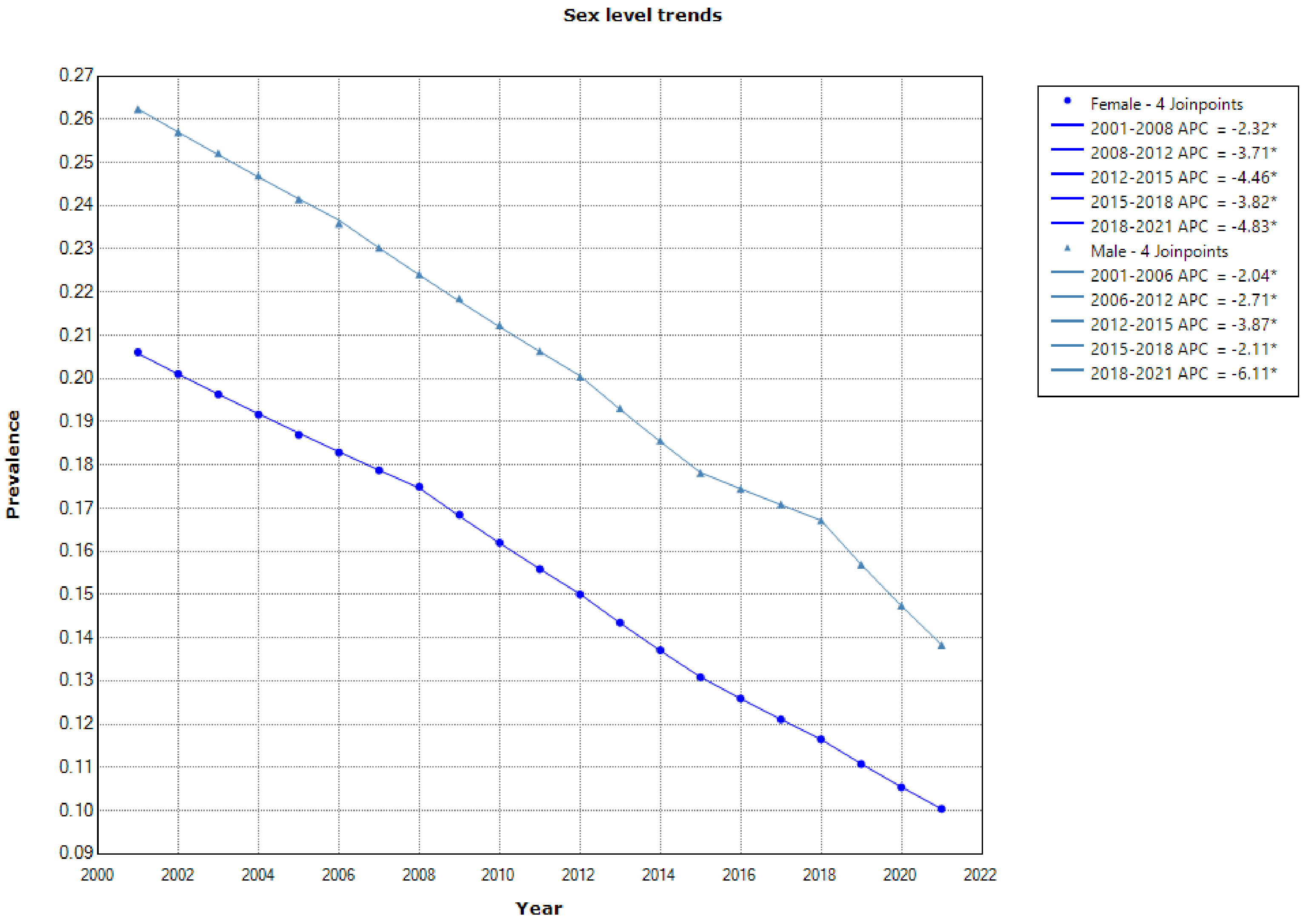

Figure S.4.34: Joinpoint regression models for the sex level trend of smoking prevalence during 2001-2021. ⋆ indicates that the APC is significantly different from zero at 5 per cent level.

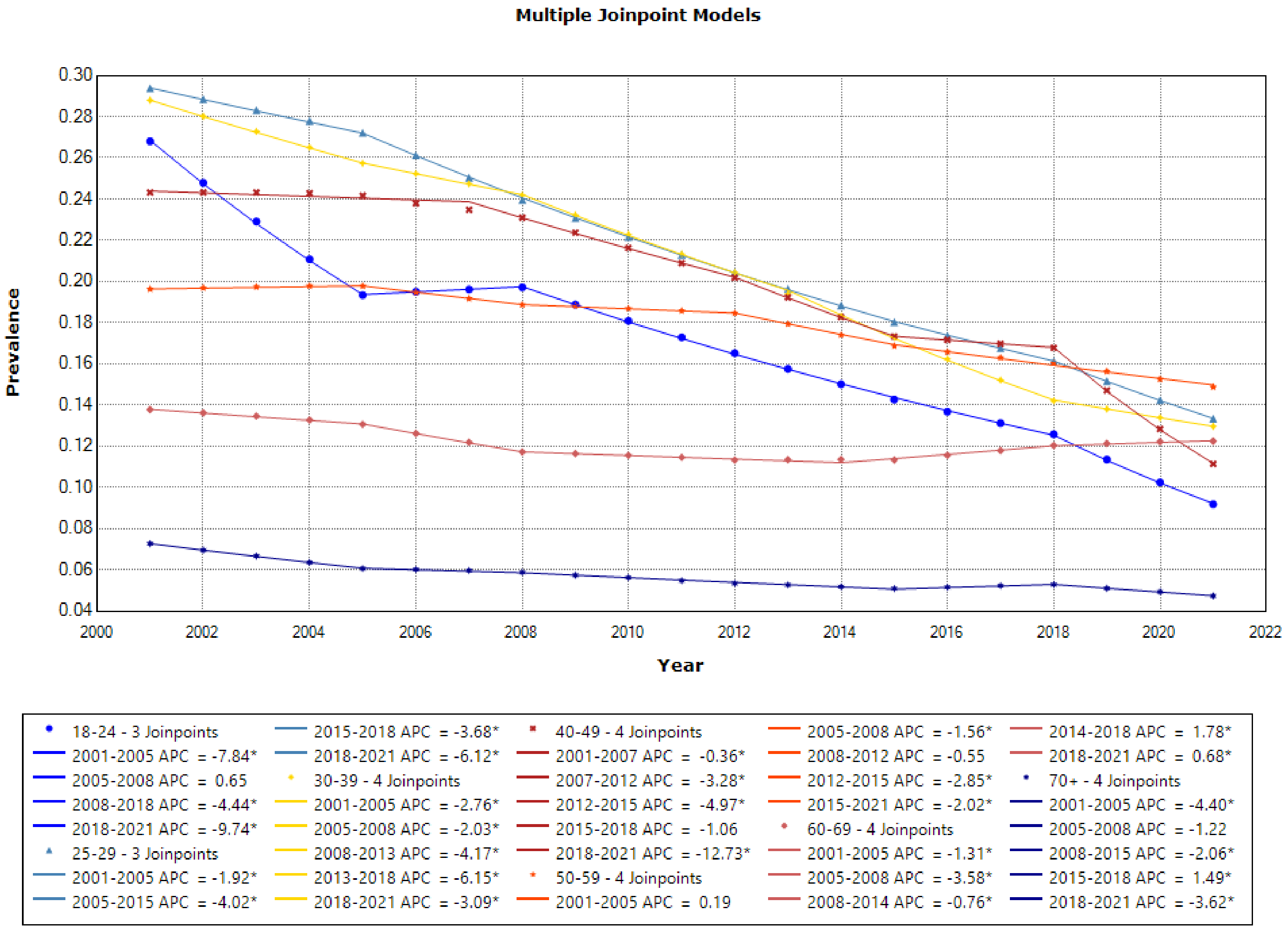

Figure S.4.35: Joinpoint regression models for the age level trend of smoking during 2001-2021. ⋆ indicates that the APC is significantly different from zero at 5 per cent level.

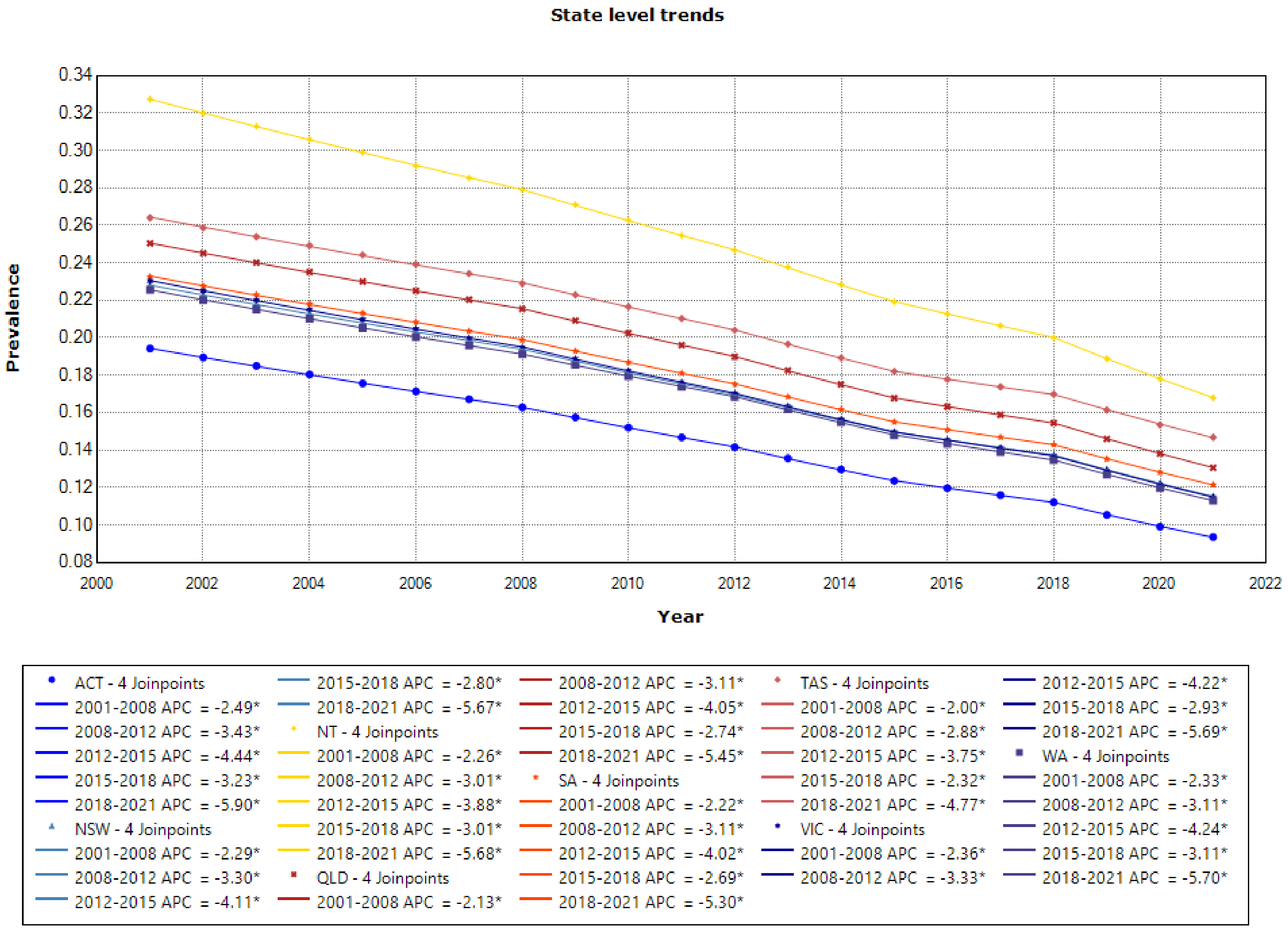

Figure S.4.36: Joinpoint regression models for the state and territory level trends of smoking prevalence during 2001-2021. ⋆ indicates that the APC is significantly different from zero at 5 per cent level.

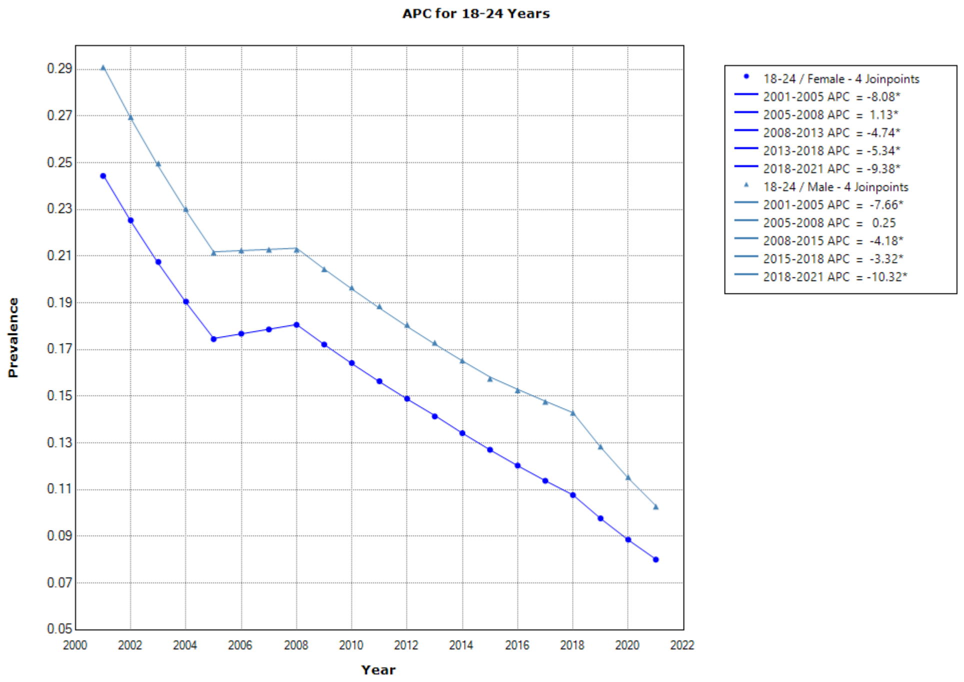

Figure S.4.37: Joinpoint regression models for the sex level trends of smoking prevalence during 2001-2021 for **18-24 years** group. ⋆ indicates that the APC is significantly different from zero at 5 per cent level.

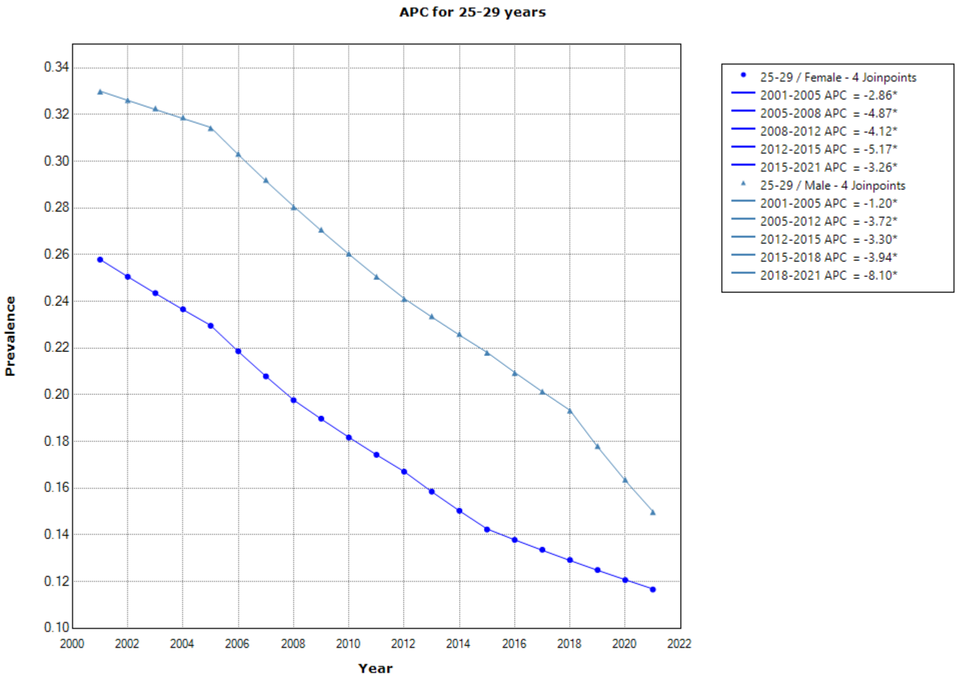

Figure S.4.38: Joinpoint regression models for the sex level trends of smoking prevalence during 2001-2021 for **25-29 years** group. ⋆ indicates that the APC is significantly different from zero at 5 per cent level.

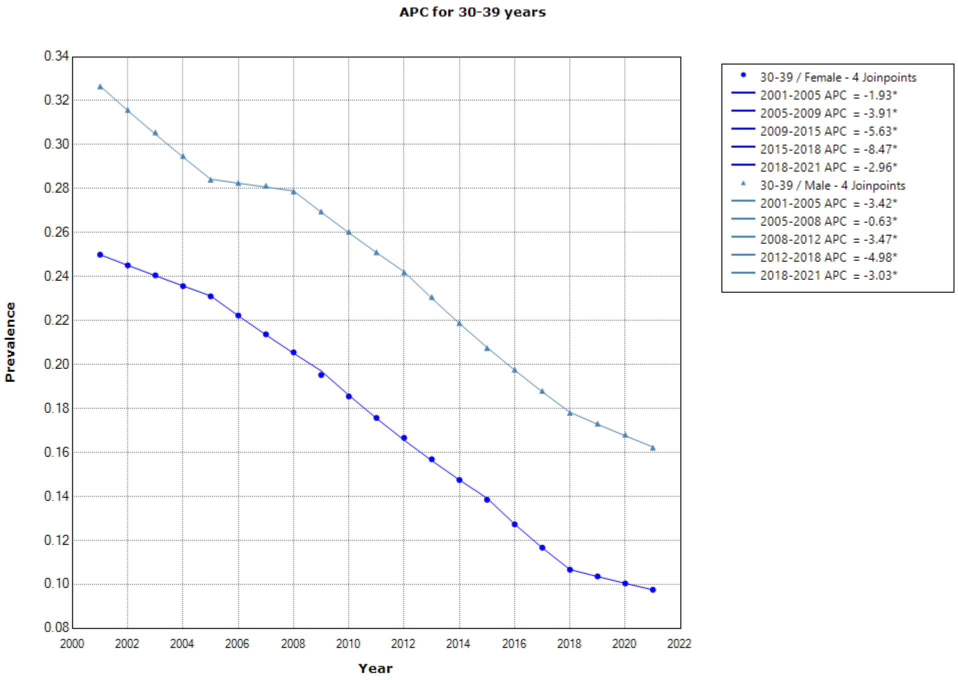

Figure S.4.39: Joinpoint regression models for the sex level trends of smoking prevalence during 2001-2021 for **30-39 years** group. ⋆ indicates that the APC is significantly different from zero at 5 per cent level.

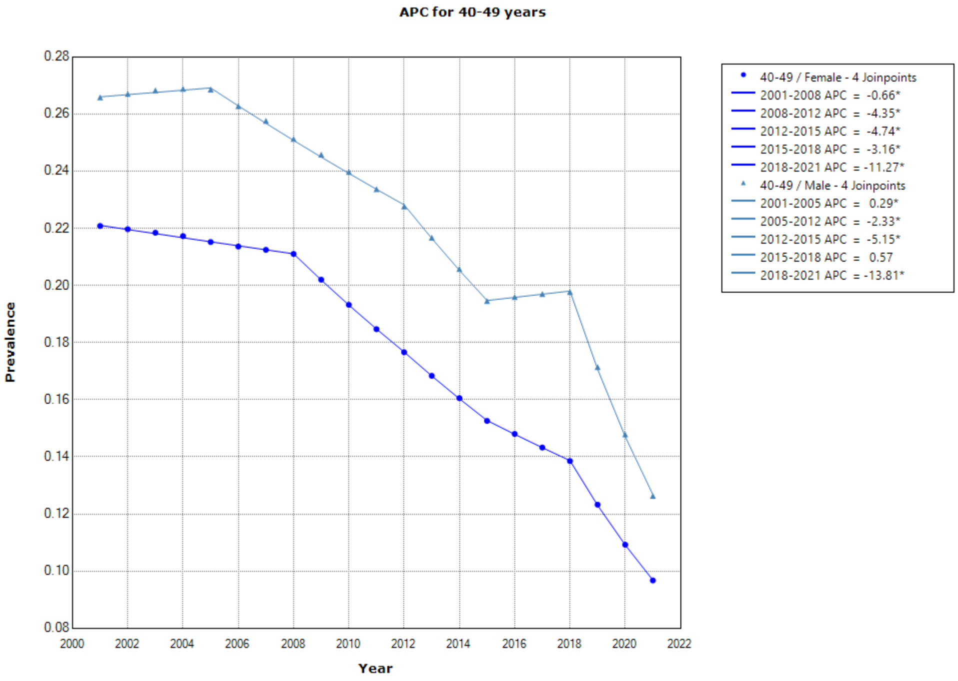

Figure S.4.40: Joinpoint regression models for the sex level trends of smoking prevalence during 2001-2021 for **40-49 years** group. ⋆ indicates that the APC is significantly different from zero at 5 per cent level.

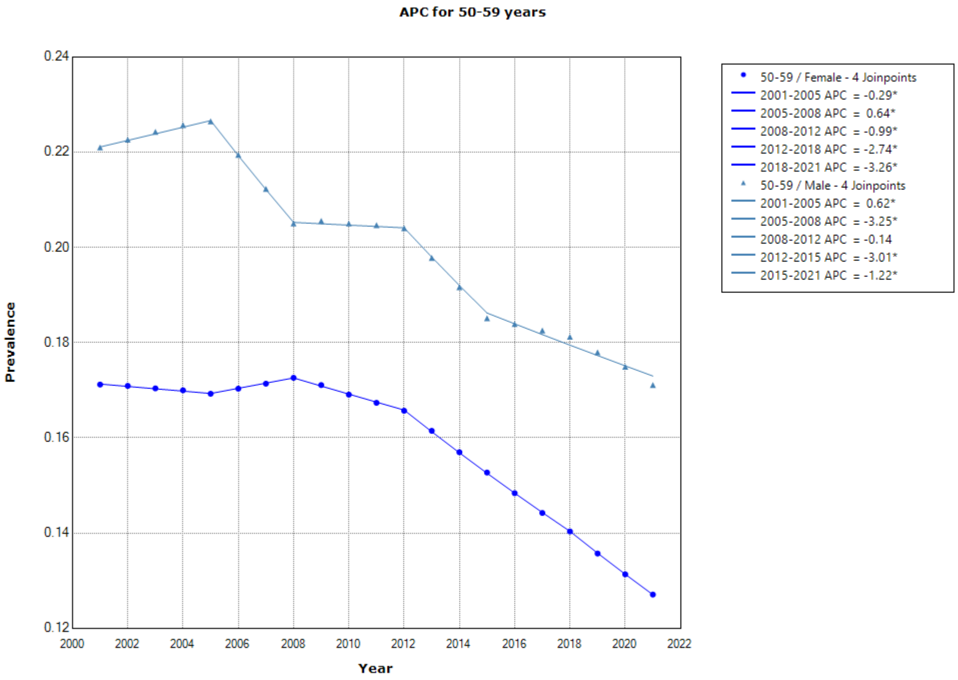

Figure S.4.41: Joinpoint regression models for the sex level trends of smoking prevalence during 2001-2021 for **50-59 years** group. ⋆ indicates that the APC is significantly different from zero at 5 per cent level.

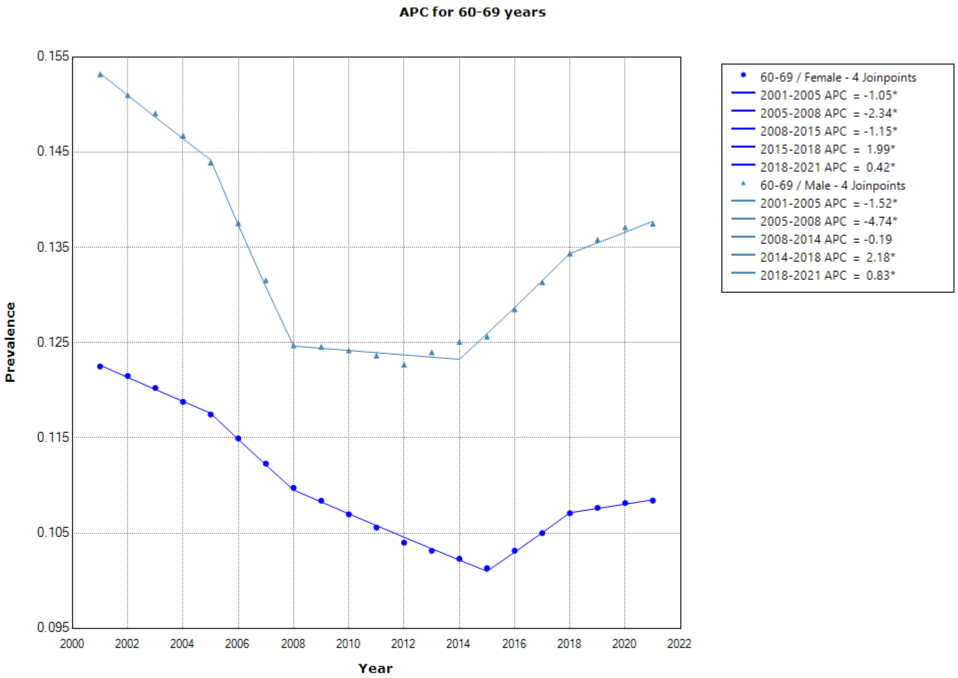

Figure S.4.42: Joinpoint regression models for the sex level trends of smoking prevalence during 2001-2021 for **60-69 years** group. ⋆ indicates that the APC is significantly different from zero at 5 per cent level.

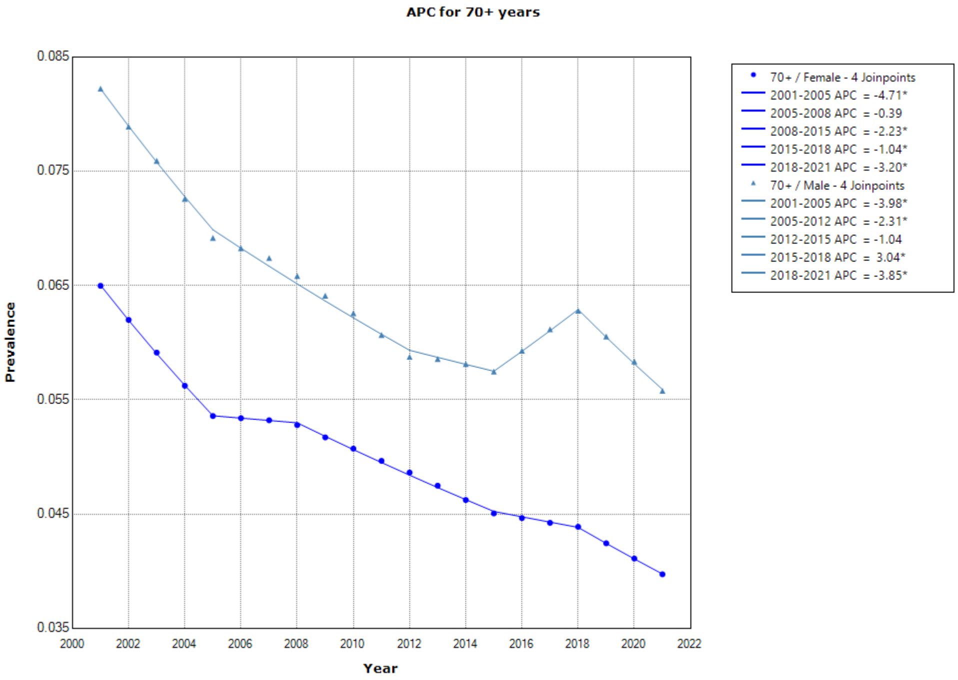

Figure S.4.43: Joinpoint regression models for the sex level trends of smoking prevalence during 2001-2021 for **70^+ years** group. ⋆ indicates that the APC is significantly different from zero at 5 per cent level.

State	Sex	Joinpoint	AAPC	AAPC LL	AAPC UL	Test Statistic	P-Value
NSW	Female	4.0	-3.7	-3.7	-3.7	-158.1	0.0
NSW	Male	4.0	-3.1	-3.2	-3.0	-65.2	0.0
VIC	Female	4.0	-3.6	-3.6	-3.5	-133.5	0.0
VIC	Male	4.0	-3.3	-3.4	-3.2	-73.5	0.0
QLD	Female	4.0	-3.2	-3.3	-3.1	-67.7	0.0
QLD	Male	4.0	-3.2	-3.3	-3.1	-64.3	0.0
SA	Female	4.0	-3.4	-3.4	-3.3	-66.7	0.0
SA	Male	4.0	-3.1	-3.2	-3.0	-71.6	0.0
WA	Female	4.0	-3.7	-3.8	-3.6	-59.8	0.0
WA	Male	4.0	-3.2	-3.2	-3.1	-82.9	0.0
TAS	Female	4.0	-3.1	-3.3	-3.0	-52.2	0.0
TAS	Male	4.0	-2.7	-2.8	-2.6	-60.3	0.0
NT	Female	4.0	-3.6	-3.7	-3.6	-150.2	0.0
NT	Male	4.0	-3.0	-3.1	-2.9	-71.6	0.0
ACT	Female	4.0	-3.9	-4.0	-3.8	-84.3	0.0
ACT	Male	4.0	-3.4	-3.5	-3.3	-66.9	0.0

Table S.4.1: Average annual percentage change (AAPC) in the smoking level at the state level by sex during 2001-2021. The lower (LL) and upper (UL) limits of 95 per cent confidence interval, test statistic value and the p-value are reported as well.

State	Age	Joinpoint	AAPC	AAPC LL	AAPC UL	Test Statistic	P-Value
NSW	18-24	3.0	-5.3	-5.4	-5.1	-71.4	< 0.001
NSW	25-29	4.0	-3.8	-3.9	-3.8	-203.0	< 0.001
NSW	30-39	4.0	-3.7	-3.8	-3.6	-76.9	< 0.001
NSW	40-49	4.0	-3.9	-4.3	-3.6	-21.7	< 0.001
NSW	50-59	4.0	-1.6	-1.9	-1.4	-12.7	< 0.001
NSW	60-69	4.0	-0.9	-1.1	-0.7	-10.2	< 0.001
NSW	70+	4.0	-2.4	-2.7	-2.1	-16.0	< 0.001
VIC	18-24	4.0	-5.2	-5.4	-5.1	-77.5	< 0.001
VIC	25-29	3.0	-4.2	-4.3	-4.1	-83.2	< 0.001
VIC	30-39	4.0	-4.1	-4.2	-4.0	-86.3	< 0.001
VIC	40-49	4.0	-3.9	-4.3	-3.6	-21.7	< 0.001
VIC	50-59	4.0	-1.3	-1.5	-1.0	-9.4	< 0.001
VIC	60-69	4.0	-0.4	-0.6	-0.2	-4.4	< 0.001
VIC	70+	4.0	-1.9	-2.1	-1.6	-12.6	< 0.001
QLD	18-24	3.0	-5.1	-5.3	-5.0	-69.6	< 0.001
QLD	25-29	3.0	-3.5	-3.6	-3.4	-76.0	< 0.001
QLD	30-39	4.0	-3.9	-4.0	-3.8	-79.8	< 0.001
QLD	40-49	4.0	-3.7	-4.0	-3.3	-21.5	< 0.001
QLD	50-59	4.0	-1.2	-1.5	-1.0	-9.2	< 0.001
QLD	60-69	4.0	-0.5	-0.6	-0.3	-5.1	< 0.001
QLD	70+	4.0	-2.1	-2.4	-1.8	-14.1	< 0.001
SA	18-24	3.0	-5.3	-5.4	-5.1	-73.2	< 0.001
SA	25-29	4.0	-3.8	-3.8	-3.8	-225.4	< 0.001
SA	30-39	4.0	-4.0	-4.1	-3.9	-82.8	< 0.001
SA	40-49	4.0	-3.9	-4.2	-3.5	-21.5	< 0.001
SA	50-59	4.0	-1.1	-1.3	-0.8	-8.4	< 0.001
SA	60-69	4.0	-0.3	-0.5	-0.1	-3.1	< 0.001
SA	70+	4.0	-2.1	-2.4	-1.8	-14.3	< 0.001
WA	18-24	3.0	-5.1	-5.3	-5.0	-70.2	< 0.001
WA	25-29	2.0	-4.0	-4.1	-3.9	-98.1	< 0.001
WA	30-39	4.0	-4.2	-4.3	-4.1	-90.2	< 0.001
WA	40-49	4.0	-3.9	-4.2	-3.5	-21.6	< 0.001
WA	50-59	4.0	-1.2	-1.4	-0.9	-9.1	< 0.001
WA	60-69	4.0	-0.5	-0.6	-0.3	-5.1	< 0.001
WA	70+	4.0	-2.0	-2.3	-1.7	-13.0	< 0.001
TAS	18-24	3.0	-5.0	-5.1	-4.9	-66.1	< 0.001
TAS	25-29	4.0	-3.6	-3.6	-3.5	-131.8	< 0.001
TAS	30-39	4.0	-3.2	-3.3	-3.1	-67.9	< 0.001
TAS	40-49	4.0	-3.2	-3.5	-2.9	-19.5	< 0.001
TAS	50-59	4.0	-1.0	-1.2	-0.7	-7.7	< 0.001
TAS	60-69	4.0	-0.9	-1.2	-0.7	-6.3	< 0.001
TAS	70+	4.0	-2.0	-2.3	-1.7	-13.3	< 0.001
NT	18-24	3.0	-4.9	-5.1	-4.8	-61.1	< 0.001
NT	25-29	4.0	-3.3	-3.4	-3.2	-62.0	< 0.001
NT	30-39	4.0	-3.8	-3.9	-3.7	-69.1	< 0.001
NT	40-49	4.0	-3.6	-3.9	-3.3	-24.5	< 0.001
NT	50-59	4.0	-1.2	-1.5	-0.9	-8.7	< 0.001
NT	60-69	4.0	-0.9	-1.1	-0.7	-10.5	< 0.001
NT	70+	4.0	-2.0	-2.3	-1.7	-12.1	< 0.001
ACT	18-24	3.0	-5.5	-5.7	-5.4	-75.8	< 0.001
ACT	25-29	3.0	-3.8	-3.9	-3.7	-65.1	< 0.001
ACT	30-39	4.0	-4.3	-4.4	-4.2	-94.9	< 0.001
ACT	40-49	4.0	-3.9	-4.3	-3.6	-22.8	< 0.001
ACT	50-59	4.0	-1.4	-1.7	-1.2	-10.5	< 0.001
ACT	60-69	4.0	-0.7	-0.9	-0.5	-7.2	< 0.001
ACT	70+	4.0	-1.9	-2.2	-1.6	-12.5	< 0.001

Table S.4.2: Average annual percentage change (AAPC) in the smoking level at the state level by age-groups during 2001-2021. The lower (LL) and upper (UL) limits of 95 per cent confidence interval, test statistic value and the p-value are reported as well.

State	Age	Sex	Joinpoint Model	AAPC	AAPC LL	AAPC UL	Test Statistic	P-Value
NSW	18-24	Female	4.0	-5.4	-5.5	-5.4	-147.5	< 0.001
NSW	18-24	Male	4.0	-5.2	-5.3	-5.0	-65.4	< 0.001
NSW	25-29	Female	4.0	-4.2	-4.3	-4.1	-112.9	< 0.001
NSW	25-29	Male	4.0	-3.5	-3.6	-3.4	-64.1	< 0.001
NSW	30-39	Female	4.0	-4.4	-4.5	-4.2	-52.2	< 0.001
NSW	30-39	Male	4.0	-3.2	-3.3	-3.1	-62.4	< 0.001
NSW	40-49	Female	4.0	-4.4	-4.5	-4.3	-85.8	< 0.001
NSW	40-49	Male	4.0	-3.5	-3.7	-3.4	-55.1	< 0.001
NSW	50-59	Female	4.0	-1.9	-1.9	-1.9	-142.0	< 0.001
NSW	50-59	Male	4.0	-1.4	-1.8	-1.0	-7.4	< 0.001
NSW	60-69	Female	4.0	-0.7	-0.9	-0.6	-8.7	< 0.001
NSW	60-69	Male	4.0	-1.0	-1.3	-0.7	-6.9	< 0.001
NSW	70+	Female	4.0	-3.0	-3.1	-2.8	-36.2	< 0.001
NSW	70+	Male	4.0	-2.0	-2.3	-1.7	-12.4	< 0.001

Table S.4.3: Average annual percentage change (AAPC) in the smoking level at the age-sex level for **New South Wales (NSW)** during 2001-2021. The lower (LL) and upper (UL) limits of 95 per cent confidence interval, test statistic value and the p-value are reported as well.

State	Age	Sex	Joinpoint Model	AAPC	AAPC LL	AAPC UL	Test Statistic	P-Value
VIC	18-24	Female	4.0	-5.5	-5.6	-5.4	-150.0	< 0.001
VIC	18-24	Male	4.0	-5.0	-5.2	-4.9	-65.1	< 0.001
VIC	25-29	Female	4.0	-3.9	-3.9	-3.8	-104.9	< 0.001
VIC	25-29	Male	4.0	-4.5	-4.6	-4.5	-89.9	< 0.001
VIC	30-39	Female	4.0	-4.9	-5.1	-4.7	-56.2	< 0.001
VIC	30-39	Male	4.0	-3.5	-3.6	-3.4	-72.7	< 0.001
VIC	40-49	Female	4.0	-4.1	-4.2	-4.0	-81.5	< 0.001
VIC	40-49	Male	4.0	-3.7	-3.8	-3.6	-57.8	< 0.001
VIC	50-59	Female	4.0	-1.3	-1.4	-1.3	-101.8	< 0.001
VIC	50-59	Male	4.0	-1.2	-1.6	-0.9	-6.4	< 0.001
VIC	60-69	Female	4.0	-0.8	-1.0	-0.7	-9.9	< 0.001
VIC	60-69	Male	4.0	0.0	-0.3	0.3	0.1	0.9
VIC	70+	Female	4.0	-2.0	-2.2	-1.8	-24.5	< 0.001
VIC	70+	Male	4.0	-1.8	-2.1	-1.5	-11.4	< 0.001

Table S.4.4: Average annual percentage change (AAPC) in the smoking level at the age-sex level for **Victoria (VIC)** during 2001-2021. The lower (LL) and upper (UL) limits of 95 per cent confidence interval, test statistic value and the p-value are reported as well.

State	Age	Sex	Joinpoint Model	AAPC	AAPC LL	AAPC UL	Test Statistic	P-Value
QLD	18-24	Female	4.0	-5.3	-5.3	-5.2	-139.0	< 0.001
QLD	18-24	Male	4.0	-5.0	-5.2	-4.9	-63.6	< 0.001
QLD	25-29	Female	4.0	-3.4	-3.5	-3.3	-98.9	< 0.001
QLD	25-29	Male	4.0	-3.6	-3.7	-3.5	-72.6	< 0.001
QLD	30-39	Female	4.0	-4.2	-4.4	-4.0	-49.8	< 0.001
QLD	30-39	Male	4.0	-3.8	-3.8	-3.7	-88.0	< 0.001
QLD	40-49	Female	4.0	-3.7	-3.8	-3.6	-77.9	< 0.001
QLD	40-49	Male	4.0	-3.6	-3.7	-3.5	-55.5	< 0.001
QLD	50-59	Female	4.0	-1.2	-1.2	-1.2	-91.1	< 0.001
QLD	50-59	Male	4.0	-1.2	-1.6	-0.9	-6.5	< 0.001
QLD	60-69	Female	4.0	-0.3	-0.5	-0.2	-3.9	< 0.001
QLD	60-69	Male	4.0	-0.5	-0.8	-0.2	-3.6	< 0.001
QLD	70+	Female	4.0	-2.1	-2.3	-2.0	-26.6	< 0.001
QLD	70+	Male	4.0	-2.1	-2.4	-1.8	-13.6	< 0.001

Table S.4.5: Average annual percentage change (AAPC) in the smoking level at the age-sex level for **Queensland (QLD)** during 2001-2021. The lower (LL) and upper (UL) limits of 95 per cent confidence interval, test statistic value and the p-value are reported as well.

State	Age	Sex	Joinpoint Model	AAPC	AAPC LL	AAPC UL	Test Statistic	P-Value
SA	18-24	Female	4.0	-5.5	-5.6	-5.4	-147.2	< 0.001
SA	18-24	Male	4.0	-5.1	-5.2	-4.9	-65.6	< 0.001
SA	25-29	Female	4.0	-3.9	-4.0	-3.8	-109.2	< 0.001
SA	25-29	Male	4.0	-3.7	-3.8	-3.6	-69.3	< 0.001
SA	30-39	Female	4.0	-4.6	-4.8	-4.5	-53.2	< 0.001
SA	30-39	Male	4.0	-3.5	-3.6	-3.4	-76.5	< 0.001
SA	40-49	Female	4.0	-3.8	-3.9	-3.7	-80.2	< 0.001
SA	40-49	Male	4.0	-3.9	-4.0	-3.7	-58.4	< 0.001
SA	50-59	Female	4.0	-1.2	-1.3	-1.2	-96.4	< 0.001
SA	50-59	Male	4.0	-1.0	-1.3	-0.6	-5.2	< 0.001
SA	60-69	Female	4.0	-0.2	-0.4	-0.1	-2.7	< 0.001
SA	60-69	Male	4.0	-0.3	-0.6	0.0	-2.0	< 0.001
SA	70+	Female	4.0	-2.6	-2.7	-2.4	-31.4	< 0.001
SA	70+	Male	4.0	-1.8	-2.1	-1.5	-11.2	< 0.001

Table S.4.6: Average annual percentage change (AAPC) in the smoking level at the age-sex level for **South Australia (SA)** during 2001-2021. The lower (LL) and upper (UL) limits of 95 per cent confidence interval, test statistic value and the p-value are reported as well.

State	Age	Sex	Joinpoint Model	AAPC	AAPC LL	AAPC UL	Test Statistic	P-Value
WA	18-24	Female	4.0	-5.4	-5.5	-5.4	-149.2	< 0.001
WA	18-24	Male	4.0	-4.9	-5.0	-4.7	-65.8	< 0.001
WA	25-29	Female	4.0	-3.9	-4.0	-3.8	-106.5	< 0.001
WA	25-29	Male	4.0	-4.1	-4.2	-3.9	-75.1	< 0.001
WA	30-39	Female	4.0	-5.3	-5.4	-5.1	-57.8	< 0.001
WA	30-39	Male	4.0	-3.5	-3.6	-3.4	-69.7	< 0.001
WA	40-49	Female	4.0	-4.1	-4.2	-4.0	-84.0	< 0.001
WA	40-49	Male	4.0	-3.7	-3.8	-3.5	-56.7	< 0.001
WA	50-59	Female	4.0	-1.5	-1.5	-1.4	-117.6	< 0.001
WA	50-59	Male	4.0	-0.9	-1.3	-0.5	-4.8	< 0.001
WA	60-69	Female	4.0	-0.4	-0.6	-0.2	-4.5	< 0.001
WA	60-69	Male	4.0	-0.5	-0.8	-0.2	-3.2	< 0.001
WA	70+	Female	4.0	-2.3	-2.5	-2.2	-28.7	< 0.001
WA	70+	Male	4.0	-1.8	-2.1	-1.5	-11.5	< 0.001

Table S.4.7: Average annual percentage change (AAPC) in the smoking level at the age-sex level for **Western Australia (WA)** during 2001-2021. The lower (LL) and upper (UL) limits of 95 per cent confidence interval, test statistic value and the p-value are reported as well.

State	Age	Sex	Joinpoint Model	AAPC	AAPC LL	AAPC UL	Test Statistic	P-Value
TAS	18-24	Female	4.0	-5.5	-5.6	-5.4	-130.5	< 0.001
TAS	18-24	Male	4.0	-4.6	-4.8	-4.5	-60.1	< 0.001
TAS	25-29	Female	4.0	-3.8	-3.9	-3.7	-101.7	< 0.001
TAS	25-29	Male	4.0	-3.4	-3.5	-3.3	-77.8	< 0.001
TAS	30-39	Female	4.0	-4.1	-4.3	-4.0	-48.3	< 0.001
TAS	30-39	Male	4.0	-2.6	-2.6	-2.5	-61.1	< 0.001
TAS	40-49	Female	4.0	-3.1	-3.2	-3.1	-72.9	< 0.001
TAS	40-49	Male	4.0	-3.2	-3.3	-3.0	-50.3	< 0.001
TAS	50-59	Female	4.0	-0.8	-0.8	-0.8	-62.1	< 0.001
TAS	50-59	Male	4.0	-1.0	-1.4	-0.7	-5.8	< 0.001
TAS	60-69	Female	4.0	-1.2	-1.3	-1.0	-14.3	< 0.001
TAS	60-69	Male	4.0	-0.7	-1.0	-0.5	-5.2	< 0.001
TAS	70+	Female	4.0	-2.4	-2.6	-2.3	-29.6	< 0.001
TAS	70+	Male	4.0	-1.6	-1.9	-1.3	-10.5	< 0.001

Table S.4.8: Average annual percentage change (AAPC) in the smoking level at the age-sex level for **Tasmania (TAS)** during 2001-2021. The lower (LL) and upper (UL) limits of 95 per cent confidence interval, test statistic value and the p-value are reported as well.

State	Age	Sex	Joinpoint Model	AAPC	AAPC LL	AAPC UL	Test Statistic	P-Value
NT	18-24	Female	4.0	-5.1	-5.2	-5.0	-126.2	< 0.001
NT	18-24	Male	4.0	-4.8	-4.9	-4.6	-58.4	< 0.001
NT	25-29	Female	4.0	-3.3	-3.4	-3.3	-93.1	< 0.001
NT	25-29	Male	4.0	-3.2	-3.3	-3.2	-84.8	< 0.001
NT	30-39	Female	4.0	-4.8	-5.0	-4.6	-52.7	< 0.001
NT	30-39	Male	4.0	-3.0	-3.1	-2.9	-79.0	< 0.001
NT	40-49	Female	4.0	-3.6	-3.6	-3.5	-92.6	< 0.001
NT	40-49	Male	4.0	-3.6	-3.7	-3.4	-55.2	< 0.001
NT	50-59	Female	4.0	-1.5	-1.5	-1.5	-111.5	< 0.001
NT	50-59	Male	4.0	-0.9	-1.3	-0.6	-5.3	< 0.001
NT	60-69	Female	4.0	-1.0	-1.2	-0.9	-12.8	< 0.001
NT	60-69	Male	4.0	-0.7	-1.0	-0.4	-5.1	< 0.001
NT	70+	Female	4.0	-2.3	-2.5	-2.2	-29.5	< 0.001
NT	70+	Male	4.0	-1.7	-2.0	-1.4	-11.3	< 0.001

Table S.4.9: Average annual percentage change (AAPC) in the smoking level at the age-sex level for **Northern Territory (NT)** during 2001-2021. The lower (LL) and upper (UL) limits of 95 per cent confidence interval, test statistic value and the p-value are reported as well.

State	Age	Sex	Joinpoint Model	AAPC	AAPC LL	AAPC UL	Test Statistic	P-Value
ACT	18-24	Female	4.0	-5.8	-5.8	-5.7	-168.1	< 0.001
ACT	18-24	Male	4.0	-5.3	-5.5	-5.2	-70.0	< 0.001
ACT	25-29	Female	4.0	-4.1	-4.2	-4.0	-113.3	< 0.001
ACT	25-29	Male	4.0	-3.6	-3.8	-3.5	-57.5	< 0.001
ACT	30-39	Female	4.0	-5.4	-5.5	-5.2	-60.6	< 0.001
ACT	30-39	Male	4.0	-3.5	-3.7	-3.4	-60.7	< 0.001
ACT	40-49	Female	4.0	-3.7	-3.8	-3.7	-78.3	< 0.001
ACT	40-49	Male	4.0	-4.1	-4.2	-4.0	-62.3	< 0.001
ACT	50-59	Female	4.0	-1.4	-1.5	-1.4	-112.3	< 0.001
ACT	50-59	Male	4.0	-1.4	-1.8	-1.0	-7.3	< 0.001
ACT	60-69	Female	4.0	-0.9	-1.1	-0.7	-10.2	< 0.001
ACT	60-69	Male	4.0	-0.4	-0.7	-0.1	-2.7	< 0.001
ACT	70+	Female	4.0	-2.3	-2.4	-2.1	-27.8	< 0.001
ACT	70+	Male	4.0	-1.6	-2.0	-1.3	-10.3	< 0.001

Table S.4.10: Average annual percentage change (AAPC) in the smoking level at the age-sex level for **Australian Capital Territory (ACT)** during 2001-2021. The lower (LL) and upper (UL) limits of 95 per cent confidence interval, test statistic value and the p-value are reported as well.

S.5 Software used for statistical computation and visualization

In this study, R [27] software has mainly been used for statistical computation and visualization of the study findings. The **joinpoint** software [14] has been utilized for conducting joinpoint trend analysis of smoking prevalence at various aggregation level. The R packages used in this study for data creation, manipulation, analysis and visualization are listed here with references.

bayesplot [?], **data.table** [?], **dplyr** [?], **foreign** [?], **ggplot2** [?], **GIGrvg** [?], **ggpubr** [?], **haven** [?], **loo** [29], **Matrix** [?], **mcmcsae** [28], **readxl** [?], **reshape2** [?], **rmarkdown** [?], **rgdal** [?], **scoringRules** [?], **sf** [?], **sp** [?], **spdep** [?], **survey** [?], **tidyverse** [?], **xtable** [?].

References

1. Banks E, Joshy G, Weber MF, Liu B, Grenfell R, Egger S, Paige E, Lopez AD, Sitas F, Beral V. Tobacco smoking and all-cause mortality in a large Australian cohort study: findings from a mature epidemic with current low smoking prevalence. *BMC Medicine* 2015;13(1):1–10. doi:https://doi.org/10.1186/s12916-015-0281-z.

2. ABS . National Health Survey: First Results methodology 2020-21. Canberra, ACT: Australian Bureau of Statistics; 2022. URL: https://www.abs.gov.au/methodologies/national-health-survey-methodology/2022.

3. Roche A, McEntee A, Kim S, Chapman J. Changing patterns and prevalence of daily tobacco smoking among Australian workers: 2007–2016. *Australian and New Zealand Journal of Public Health* 2021;doi:10.1111/1753-6405.13126.

4. McEntee A, Kim S, Harrison N, Chapman J, Roche A. Patterns and prevalence of daily tobacco smoking in Australia by industry and occupation: 2007-2016. *Nicotine & Tobacco Research* 2021;doi:10.1093/ntr/ntab126.

5. Hay D. The rise and fall of smoking in New Zealand. *Journal of the Royal College of Physicians of London* 1993;27(3):315.

6. Santiago-Pérez MI, López-Vizcaíno E, Pérez-Ríos M, Guerra-Tort C, Rey-Brandariz J, Varela-Lema L, Martín-Gisbert L, Ruano-Ravina A, Schiaffino A, Galán I, et al. Small-area models to assess the geographical distribution of tobacco consumption by sex and age in Spain. *Tobacco Induced Diseases* 2023;21. doi:10.18332/tid/162379.

7. Thun MJ, Carter BD, Feskanich D, Freedman ND, Prentice R, Lopez AD, Hartge P, Gapstur SM. 50-year trends in smoking-related mortality in the United States. *Nwe England Journal of Medicine* 2013;368:351–64. doi:10.1056/NEJMsa1211127.

8. ABS . National Health Survey 2017-18, First Results. Canberra, ACT: Australian Bureau of Statistics; 2019. URL: https://www.abs.gov.au/statistics/health/health-conditions-and-risks/national-health-survey/2017-18.

9. AIHW . National Drug Strategy Household Survey 2019. Drug Statistics series no. 32. PHE 270. Australian Institute of Health & Welfare (AIHW); 2020. doi:10.25816/e42p-a447.

10. Rao J, Molina I. Small Area Estimation, 2nd Edition. Wiley-Interscience; 2015.

11. Boonstra HJ, van den Brakel J, Das S. Multilevel time series modelling of mobility trends in the netherlands for small domains. *Journal of the Royal Statistical Society Series A: Statistics in Society* 2021;184(3):985–1007. URL: https://doi.org/10.1111/rssa.12700.

12. Das S, van den Brakel J, Boonstra HJ, Haslett S. Multilevel time series modelling of antenatal care coverage in Bangladesh at disaggregated administrative levels. *Survey Methodology* 2022;48(2):401–37. URL: http://www.statcan.gc.ca/pub/12-001-x/2022002/article/00010-eng.htm.

13. Clegg LX, Hankey BF, Tiwari R, Feuer EJ, Edwards BK. Estimating average annual per cent change in trend analysis. *Statistics in Medicine* 2009;28(29):3670–82. doi:https://doi.org/10.1002/sim.3733.

14. National Cancer Institute . Joinpoint Regression Software. Version 4.9.1.0. Statistical Methodology and Applications Branch, Surveillance Research Program, National Cancer Institute.; Maryland, USA; 2022. URL: https://surveillance.cancer.gov/joinpoint/.

15. Rahib L, Wehner MR, Matrisian LM, Nead KT. Estimated projection of US cancer incidence and death to 2040. *JAMA Network Open* 2021;4(4):e214708. doi:10.1001/jamanetworkopen.2021.4708.

16. Dyvesether SM, Nordentoft M, Forman JL, Erlangsen A. Joinpoint regression analysis of suicides in Denmark during 1980-2015. *Danish Medical Journal* 2018;65(4):A5477. doi:https://content.ugeskriftet.dk/sites/default/files/scientific_article_files/2018-08/a5477_0.pdf.

17. Das S, Baffour B, Richardson A. Trends in chronic childhood undernutrition in Bangladesh for small domains. *Population Studies* 2023;:1–19doi:https://doi.org/10.1080/00324728.2023.2239772.

18. ABS . 4364.0 - National Health Survey: Summary of Results, 2001. Canberra, ACT: Australian Bureau of Statistics; 2002. URL: https://www.ausstats.abs.gov.au/ausstats/subscriber.nsf/0/90A3222FAD5E3563CA256C5D0001FD9D/$File/43640_2001.pdf.

19. ABS . 4364.0 - National Health Survey: Summary of Results, 2004-05. Canberra, ACT: Australian Bureau of Statistics; 2006. URL: https://www.ausstats.abs.gov.au/ausstats/subscriber.nsf/0/3B1917236618A042CA25711F00185526/$File/43640_2004-05.pdf.

20. ABS . 4364.0 - National Health Survey: Summary of Results, 2007-2008. Canberra, ACT: Australian Bureau of Statistics; 2009. URL: https://www.abs.gov.au/ausstats/abs@.nsf/Latestproducts/4364.0Main%20Features42007-2008%20(Reissue).

21. ABS . 4364.0.55.001 - Australian Health Survey: First Results, 2011-12. Canberra, ACT: Australian Bureau of Statistics; 2012. URL: https://www.ausstats.abs.gov.au/ausstats/subscriber.nsf/0/1680ECA402368CCFCA257AC90015AA4E/$File/4364.0.55.001.pdf.

22. ABS . 4364.0.55.001 - National Health Survey: First Results, 2014-15. Canberra, ACT: Australian Bureau of Statistics; 2015. URL: https://www.ausstats.abs.gov.au/ausstats/subscriber.nsf/0/CDA852A349B4CEE6CA257F150009FC53/$File/national%20health%20survey%20first%20results,%202014-15.pdf.

23. ABS . National Health Survey: Users' Guide Electronic, 2007-08. 4363.0.55.001. Canberra, ACT: Australian Bureau of Statistics; 2008. URL: https://www.ausstats.abs.

gov.au/ausstats/subscriber.nsf/0/CC0FB5A08570984ECA25762E0017CF2B/$File/4363055001_2007-08.pdf.

24. Liu B, Lahiri P, Kalton G. Hierarchical Bayes modeling of survey-weighted small area proportions. *Survey Methodology* 2014;40(1):1–13. URL: https://www150.statcan.gc.ca/n1/en/pub/12-001-x/2014001/article/14030-eng.pdf?st=rBrvhU1Y.

25. Chandra H, Chambers R, Salvati N. Small area estimation of survey weighted counts under aggregated level spatial model. *Survey Methodology* 2019;45(1):31–59. URL: https://www150.statcan.gc.ca/n1/pub/12-001-x/2019001/article/00006-eng.htm.

26. Ghosh M. Small area estimation: Its evolution in five decades. *Statistics in Transition New Series* 2020;21(4):1–22. doi:https://doi.org/10.21307/stattrans-2020-022.

27. R Core Team . R: A Language and Environment for Statistical Computing. R Foundation for Statistical Computing; Vienna, Austria; 2015. URL: http://www.R-project.org.

28. Boonstra HJ. mcmcsae: MCMC Small Area Estimation; 2023. URL: https://CRAN.R-project.org/package=mcmcsae; R package version 0.7.6.

29. Vehtari A, Gabry J, Magnusson M, Yao Y, Bürkner PC, Paananen T, Gelman A, Goodrich B, Piironen J, Nicenboim B. loo: Efficient Leave-One-Out Cross-Validation and WAIC for Bayesian Models; 2022. URL: https://CRAN.R-project.org/package=loo; R package version 2.5.1.

30. Vehtari A, Gelman A, Gabry J. Practical Bayesian model evaluation using leave-one-out cross-validation and WAIC. *Statistics and Computing* 2017;27(5):1413–32. doi:https://doi.org/10.1007/s11222-016-9696-4.

31. Gabry J, Simpson D, Vehtari A, Betancourt M, Gelman A. Visualization in Bayesian workflow. *Journal of the Royal Statistical Society Series A: Statistics in Society* 2019;182(2):389–402. doi:https://doi.org/10.1111/rssa.12378.

32. ABS . National, state and territory population. Australian Bureau of Statistics (ABS); 2022. URL: https://www.abs.gov.au/statistics/people/population/national-state-and-territory-population/latest-release.

33. Guerra-Tort C, López-Vizcaíno E, Santiago-Pérez MI, Rey-Brandariz J, Candal-Pedreira C, Varela-Lema L, Schiaffino A, Ruano-Ravina A, Perez-Rios M. Validation of a small-area model for estimation of smoking prevalence at a subnational level. *Tobacco Induced Diseases* 2023;21. doi:10.18332/tid/169683.

34. Smith DR, Leggat PA. The historical decline of tobacco smoking among Australian physicians: 1964–1997. *Tobacco induced diseases* 2008;4(1):1–9. doi:10.1186/1617-9625-4-13.

35. Gartner C, Perusco A, Puljević C, Morphett K, Hefler M. International progress toward a commercial tobacco endgame is an opportunity for advancing tobacco control in Australia. *Australian and New Zealand Journal of Public Health* 2023;47(2):100029. doi:10.1016/j.anzjph.2023.100029.

36. ABS . 2018/19 National Aboriginal and Torres Strait Islander Health Survey. Expanded Confidentialised Unit Record File (CURF), DataLab. Australian Bureau of Statistics (ABS); 2021. URL: https://www.abs.gov.au/statistics/people/aboriginal-and-torres-strait-islander-peoples/national-aboriginal-and-torres-strait-islander-health-survey/latest-release.

Appendix Figures

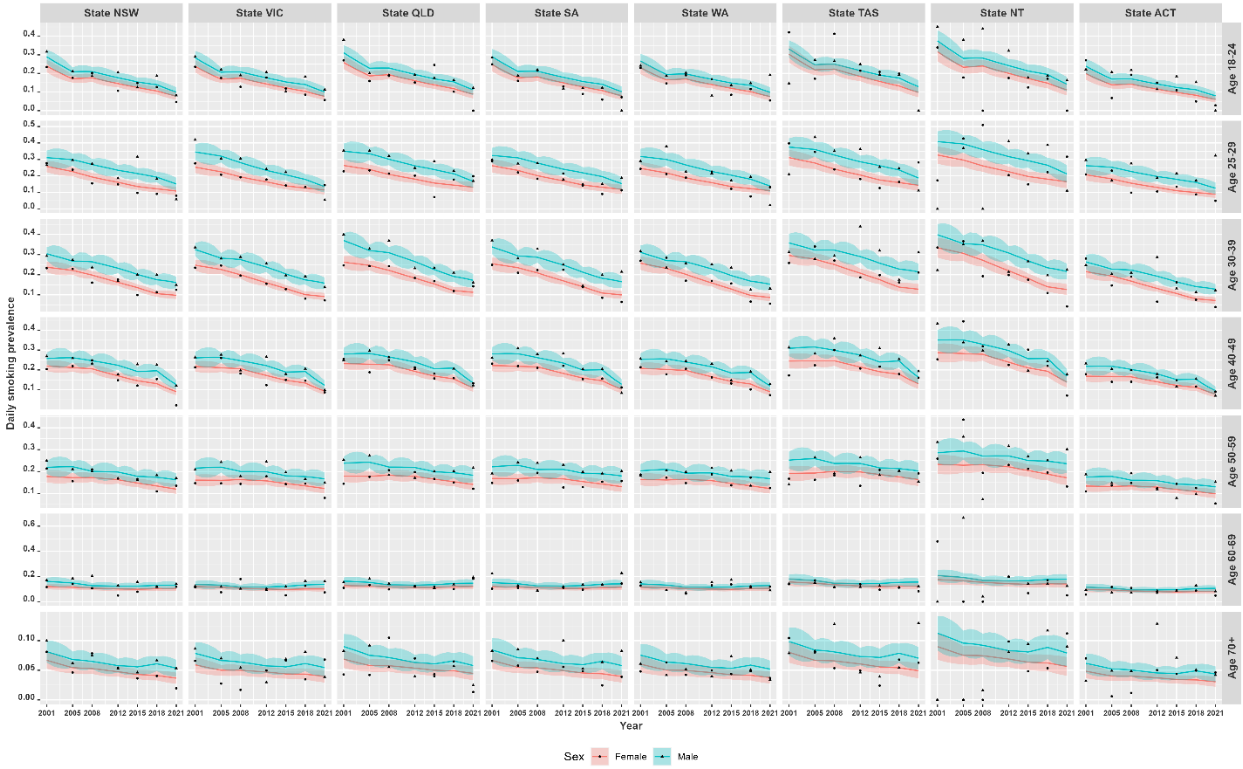

Figure S.1: Trends of daily smoking prevalence in Australia during 2001-2021 for the detailed cross-classified domains of seven age groups (18-24, 25-29, 30-39, 40-49, 50-59, 60-69, and 70^{+} years), two sex (female and male) groups, six states (NSW, VIC, QLD, SA, and WA) and two territories (NT and ACT) estimated by the direct estimator (black dots) and model-based estimator (solid lines with 95% confidence band). Direct estimates of smoker numbers obtained from the Australian Nation Health Surveys (2001, 2005, 2008, 2012, 2015, 2018, and 2021) are used in the time-series model development to obtain model-based estimates.

National level trend: All: 4 Joinpoints

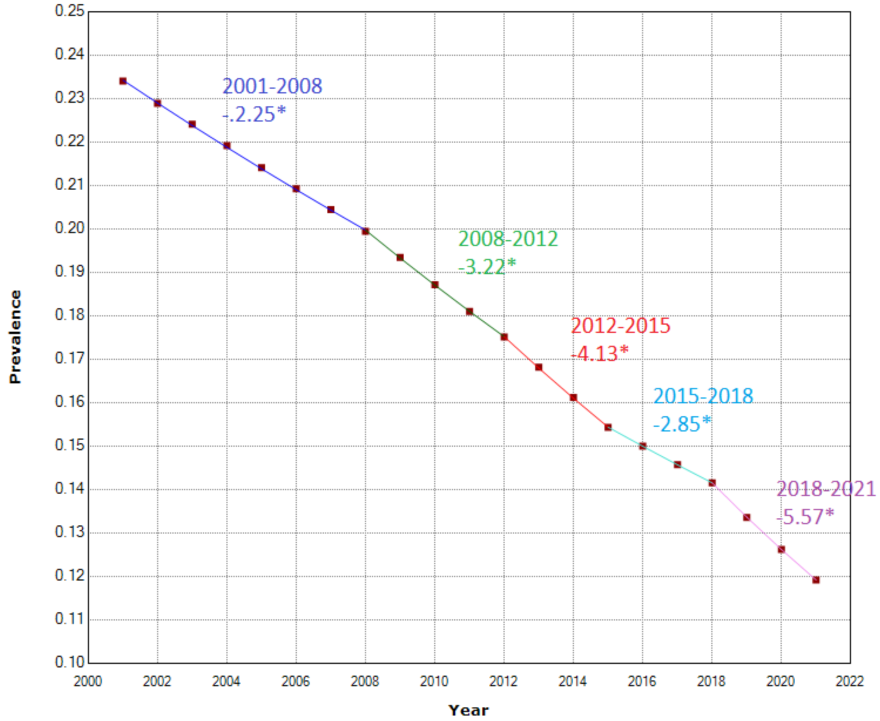

* Indicates that the Annual Percent Change (APC) is significantly different from zero at the alpha = 0.05 level
Final Selected Model: 4 Joinpoints.

Sex level trends

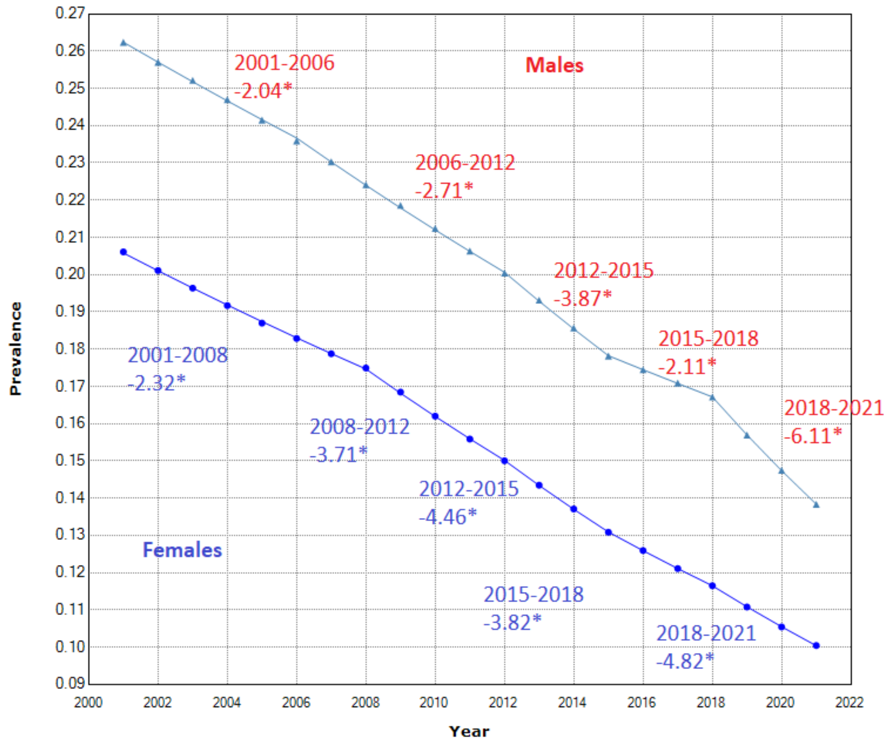

Figure S.2: Annual percentage change (APC) in the national and sex level daily smoking prevalence during 2001-2021 in Australia. The model-based estimates of daily smoking prevalence for the considered domains during the reference period are used to develop the joinpoint regression models. The APC value obtained from the joinpoint regression model is reported in each segment. Statistically significant changes are denoted by ‘*’.

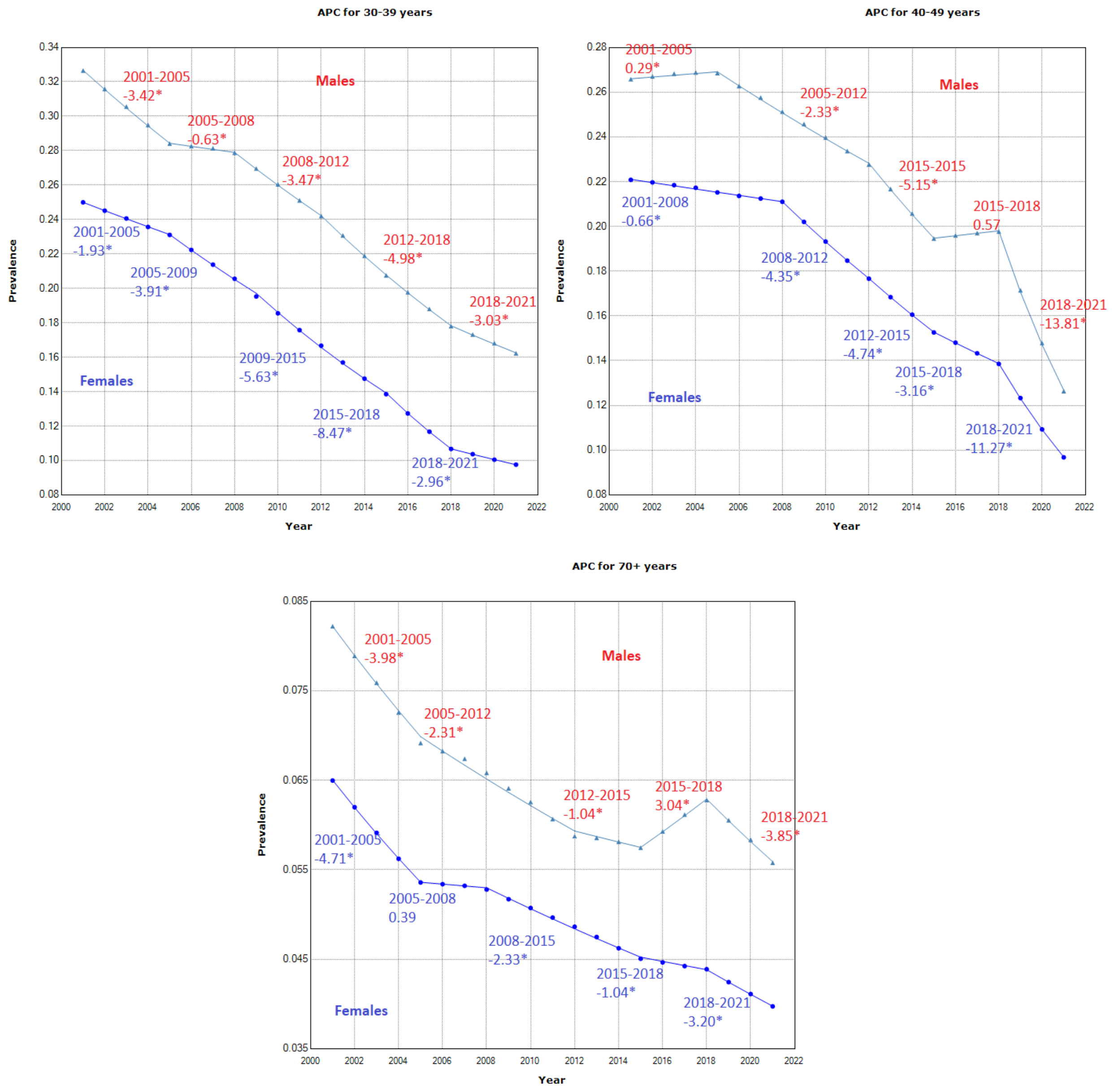

Figure S.3: Annual percentage change (APC) in daily smoking prevalence by sex for 30-39, 40-49, and 70^{+} years age groups in Australia during 2001-2021. The model-based estimates of daily smoking prevalence for the considered domains during the reference period are used to develop the joinpoint regression models. The APC value obtained from the joinpoint regression model is reported in each segment. Statistically significant changes are denoted by ‘*’.

© 2024 Das S. et al.
